# Hyaluronan Hybrid Cooperative Complexes as a Novel Frontier for Cellular Bioprocesses Re-Activation

**DOI:** 10.1371/journal.pone.0163510

**Published:** 2016-10-10

**Authors:** Antonietta Stellavato, Luisana Corsuto, Antonella D’Agostino, Annalisa La Gatta, Paola Diana, Patrizia Bernini, Mario De Rosa, Chiara Schiraldi

**Affiliations:** 1 Department of Experimental Medicine, Section of Biotechnology, Medical Histology and Molecular Biology, Second University of Naples, Bioteknet, Naples, Italy; 2 IBSA Farmaceutici Italia Srl, Lodi, Italy; University of Patras, GREECE

## Abstract

Hyaluronic Acid (HA)-based dermal formulations have rapidly gained a large consensus in aesthetic medicine and dermatology. HA, highly expressed in the Extracellular Matrix (ECM), acts as an activator of biological cascades, stimulating cell migration and proliferation, and operating as a regulator of the skin immune surveillance, through specific interactions with its receptors. HA may be used in topical formulations, as dermal inducer, for wound healing. Moreover, intradermal HA formulations (injectable HA) provide an attractive tool to counteract skin aging (e.g., facial wrinkles, dryness, and loss of elasticity) and restore normal dermal functions, through simple and minimally invasive procedures. Biological activity of a commercially available hyaluronic acid, Profhilo®, based on NAHYCO™ technology, was compared to H-HA or L-HA alone. The formation of hybrid cooperative complexes was confirmed by the sudden drop in η_0_ values in the rheological measurements. Besides, hybrid cooperative complexes proved stable to hyaluronidase (BTH) digestion. Using *in vitro* assays, based on keratinocytes, fibroblasts cells and on the Phenion^®^ Full Thickness Skin Model 3D, hybrid cooperative complexes were compared to H-HA, widely used in biorevitalization procedures, and to L-HA, recently proposed as the most active fraction modulating the inflammatory response. Quantitative real-time PCR analyses were accomplished for the transcript quantification of collagens and elastin. Finally immunofluorescence staining permitted to evaluate the complete biosynthesis of all the molecules investigated. An increase in the expression levels of type I and type III collagen in fibroblasts and type IV and VII collagen in keratinocytes were found with the hybrid cooperative complexes, compared to untreated cells (CTR) and to the H-HA and L-HA treatments. The increase in elastin expression found in both cellular model and in the Phenion^®^ Full Thickness Skin Model 3D also at longer time (up to 7 days), supports the clinically observed improvement of skin elasticity. The biomarkers analyzed suggest an increase of tissue remodeling in the presence of Profhilo^®^, probably due to the long lasting release and the concurrent action of the two HA components.

## Introduction

As a result of its physico-chemical and rheological properties such as viscoelasticity and capacity to retain water, Hyaluronic Acid (HA) plays a pivotal role in the control of tissue hydration and permeability to small or large molecules, and these properties contribute to its excellent biocompatibility and immune barrier function. In the human dermis, the high percentage of HA allows hydration, maintaining at the same time a proper tissue volume which buffers skin cells from mechanical damage. Alone or in combination with other molecules, HA accelerates *in vitro* processes related to wound healing [1] and *in vivo* tissue regeneration (e.g., burns, ulcers) [2]. Furthermore, HA can provide both anti-inflammatory and bio-stimulating effect, and also activate other signaling pathways through the interaction with cell membrane receptors such as CD44, TLR-4, and RHAMM [3]. Besides, hyaluronans recently proved the ability to prevent ROS damage [4]. Moreover, HA is a molecule critically involved in skin aging. All the above mentioned properties make HA an excellent dermal agent, able to correct soft tissue defects of the face and prompt collagen biosynthesis, leading to rejuvenation effects [5]. The aging process is affected by environmental factors (as free radicals formation) and individual genetic background. In particular, skin aging process could be viewed as an alteration occurring in collagen, elastin and, mainly, in HA content [6]. The skin matrix is indeed responsible for structural integrity, mechanical elasticity, stability and many other functions. HA degradation, together with the bone resorption, plays a major role in the formation of wrinkles and other signs of aging, and structural proteins are essential to skin health and youthfulness [7, 8]. Skin aging can also be related to various tissue changes in different skin layers, such as destruction of the epidermal–dermal interface, dermal atrophy, and loss of elastic tissue in the sub-epidermal elastin fibers network [9, 10]. The ratio between different collagen types may also change, affecting several skin functions. With aging, type I and type III collagen production and epidermal matrix turnover rate are reduced [9]. Other scientists found the ratio of type III to type I collagen increased, principally due to a drop of collagen I biosynthesis in elderly women [11, 12]. Type IV and VII collagen are implicated in skin homeostasis [13]: type IV collagen is found primarily within the basement membrane zone and plays a key role in maintaining mechanical firmness while type VII collagen is critical for anchoring fibrils of the basement membrane to the underlying dermal tissue. It was hypothesized that wrinkles formation is also driven by the reduction in the aged skin of these abovementioned types of collagen [14]. As for skin homeostasis, the reduction of HA may lead to physiological alterations of keratinocytes and fibroblasts [15, 16]. The use of HA moderately improved epidermal homeostasis barrier and tissue thickness in murine models [17]. Since the early 1980s, all these data has led to the extensive use of HA injections for correction of wrinkles and rejuvenation procedures [12]. Currently, a lot of dermal hyaluronic acid-based medical devices are used in the aesthetic medicine, most of which are based on chemical cross-linking; this chemical modification improves stability, rigidity, and elasticity (elastic modulus G’ and viscous G”), but substantially modifies the natural molecule structure. Topical or injected H-HA products have had variable effects in restoring a physiological and hydrated microenvironment typical of youthful skin [18, 19]. In this study, we evaluated the *in vitro* cellular and molecular changes both in dermal keratinocytes and fibroblasts and in following the use of a new hyaluronic acid-based formulation named Profhilo^®^, in comparison to High (H-HA) and Low Molecular Weight HA (L-HA) gels. Type I, III, IV and VII collagen and elastin gene expression were evaluated by quantitative real-time PCR in keratinocytes and fibroblasts monocultures. Besides, we used immunofluorescence staining to assess the expression and localization of ECM proteins, such as type I and III collagen and elastin, in keratinocytes and fibroblast co-cultures treated with hybrid cooperative complexes, compared to H-HA, L-HA and to untreated cells. In addition we run a full set of experiments using the Phenion^®^ Full Thickness Skin Model 3D for gene expression on all markers at 24h and 7 days and immunofluorescence staining (for Elastin) already performed on co-cultures cell monolayer.

## Materials and Methods

### Materials

High- (H-HA; MW 1200 ±100 kDa) and low-molecular weight hyaluronic acid (L-HA: Mw = 100 ± 10) were produced by Altergon s.r.l., Italy, from *Streptococcus equi* ssp. *equi* as a fermentative ultrapure pharmaceutical grade HA (i.e. purity > 95%, water content < 10%, EU/mg < 0.05, extremely low heavy metals content). The product under investigation has a patented technology (WO/2012/032, 151), named NAHYCO^™^ technology [20], based on thermal procedures for the formation of hybrid cooperative complexes of hyaluronic acid, starting from an initial mixture of an equal amount (ratio 1:1) of H-HA (Mw = 1200±100 kDa Mw/Mn = 1.4) and L-HA (M_w_ = 100±10kDa, Mw/Mn = 1.4). The final concentration used in these experiments was 32g/L: 32 mg H-HA + 32 mg L-HA in 2 mL volume, provided in prefilled syringes.

### Rheology measurements

Dynamic viscosity measurements were carried out using a Physica MCR301 oscillatory rheometer (Anton Paar, Germany) equipped with a coaxial cylinders geometry and a Peltier temperature control. The flow curves were built with fifty measurements at 25°C, in a range of shear rate 0.001-300s^-1^. From each flow curve, the zero-shear viscosity (η_0_) was the viscosity value in the range of Newtonian plateau. Flow curves were registered for different sample types:

1. H-HA (16 g/L, pre and post thermal treatment): high molecular weight hyaluronic acid, solubilized at room temperature, subjected or not to thermal treatment.
2. H-HA+L-HA without thermal treatment (16 g/L + 16g/L): high and low molecular weight hyaluronic acid solubilized at room temperature (32 g/L final concentration).
3. H-HA/L-HA hybrid cooperative complexes: high and low molecular weight hyaluronic acid solubilized at room temperature and submitted to thermal treatment (32 g/L final concentration, initial H/L ratio 1:1).
4. H-HA+L-HA mixed after separate thermal treatment: high and low molecular weight hyaluronic acid solubilized in different vials (each containing 32 g/L either of H-HA or of L-HA), subjected to thermal treatments separately and then, after cooling to room temperature, mixed 1:1 (v/v) (final concentration 32 g/L).

### Stability to hyaluronidase

Samples of the H-HA/L-HA hybrid cooperative complexes were diluted to 4 mg/mL in phosphate buffered solution (PBS) and incubated in the presence of BTH (1 U/mL) at 37°C under stirring conditions (1000 rpm). At different incubation times, ranging from 0.5h to 10 days, the samples were withdrawn, boiled for 10 min to inactivate the enzyme, and then appropriately diluted for chromatographic analysis. In particular, a Size Exclusion Chromatography-Triple Detector Array (SEC-TDA) equipment (Viscotek, Lab Service Analytica S.R.L., Italy) was used. A detailed description of the SEC-TDA system and analysis conditions are reported elsewhere [21, 22]. Sample molecular weight (M_w_, M_n_, M_w_/M_n_), molecular size (hydrodynamic radius-Rh) and intrinsic viscosity ([η]) distributions were derived. The degradation was monitored by following the decrease in sample fraction (w/w %) having M_w_ higher than 1MDa. A linear H-HA (M_w_ = 1120 ± 100kDa; M_w_/M_n_ = 1.5±0.1) was used as control.

### Cell cultures

To evaluate the biological properties of HA hybrid complexes, human dermal keratinocytes (HaCat) and fibroblasts (HDF) cultures, were used as cellular models of human skin. HaCaT, a spontaneously transformed non-tumorigenic human keratinocytes cell line was provided by Istituto Zooprofilattico, Brescia, Italy and the cells were cultured in Dulbecco’s Modified Eagle Medium (DMEM), supplemented with 10% (v/v) heat inactivated Fetal Bovine Serum (FBS), penicillin 100 U/ml and streptomycin 100 μg/ml. DMEM, FBS, Pen-Strep PBS and Trypsin were provided by Gibco Invitrogen (Milan, Italy). A human dermal fibroblasts cell line immortalized with hTERT (HDF cells, BJ-5ta, ATCC CRL-4001), was cultured in a 4:1 mixture of DMEM and Medium199 supplemented with 0.01mg/ml hygromycin B and 10% (v/v) FBS. All materials for HDF culture were purchased from ATCC (USA). The cells were grown on tissue culture plates (BD Falcon, Italy), using an incubator with a humidified atmosphere (95% air/5%CO_2_ v/v) at 37°C. For the gene expression analyses, human keratinocytes and fibroblasts were grown in different cell cultures. More in detail, 3.75x10^4^cells/cm^2^ in a standard 24-well culture plate were seeded. For the immunofluorescence staining, HaCat/HDF co-cultures were used. In this model, human keratinocytes and fibroblasts were seeded, cultured together and then analyzed on a glass microscope slide. μ-dish (35 mm, high) culture-insert (Ibidi, Integrated BioDiagnostics, Munich, Germany) were used to separate the two cell populations and to allow cell interaction. Cells were seeded directly on chamber affixed to a specially glass microscope slide (BD Falcon™ BD Biosciences) at a ratio of 1:1.25 HaCat:HDF (1x10^3^ /cm^2^ and 1.25x10^3^ /cm^2^ respectively). In both models, cells were treated with H-HA 1400kDa (0.16% w/w), L-HA 100kDa (0.16% w/w) and hybrid cooperative complexes (H-HA/L-HA complexes 0.16% w/w). Treatments lasted 4 and 24h for the gene expression analyses and 24h for the fluorescence staining.

### 3D Skin Model

The Phenion^®^ Full Thickness Skin Model, produced by Henkel (Düsseldorf, Germany, diameter 1.3 cm) was used for the study. In this model, epidermal keratinocytes and dermal fibroblasts (derived from biopsy material from healthy donors) form a multilayered skin equivalent under culture conditions that resembles human skin multilayered structure and tissue functionality [23]. Upon arrival, the FT models were removed immediately from the semi-solid transport medium. Three lower halves of small size Petri dishes filled with 2 sterile metal supports were placed in a large Petri dish (Φ = 100mm). The small Petri dishes were filled with 5 ml of ALI^®^ medium provided with the skin models were immediately transferred in an incubator (37°C, 5% CO2). 6 small injections of 50 μl of Hybrid complexes (Profhilo^®^ gel) and H-HA or L-HA in six different points of the model just below the epidermal surface in the dermal compartment were performed. The samples were then incubated for 24h and 7 days. Control samples, were FT-SKIN injected with PBS solution (6 injections of 50 μl). The effect of Profhilo^®^ formulation, H-HA or L-HA alone, were analyzed for their effect on production of type I collagen (COLI), type III collagen (COLIII), type IV collagen (COLIV), type VII collagen (COLVII) and Elastin (ELS).

At 24h and 7 days the FT models were opportunely cut with a sterile blade in different sections and immersed in appropriately buffer for successive experiments, in particular real time PCR and immunofluorescence staining.

### RNA extraction and quantitative real-time PCR on cell monolayer

The expression of each mRNA encoding ECM proteins such as type I (COLIA1), III (COLIIIA1), IV (COLIVA1) and VII (COLVIIA1) collagens and elastin (ELS) was assayed by quantitative real-time PCR (qRT-PCR). Total RNA was extracted from human dermal keratinocytes and fibroblasts using TRIzol^®^ (Invitrogen, Milan, Italy), according to the manufacturer’s procedures fully described in D’Agostino et al. (2015). In brief, one μg of DNase-digested total RNA (DNA-free kit; Ambion-Applied Biosystems) was converted to cDNA using Reverse Transcription System Kit (Promega, Milan, Italy). PCR was then performed using iQ™ SYBR^®^Green Supermix (Bio-Rad Laboratories s.r.l., Milan, Italy) to analyze, with the appropriate primer pairs, the expression levels of collagen and elastin. The primer sequences and amplification protocol are reported in Table 1. All reactions were performed in triplicate, and the relative expression of specific mRNA was determined by normalizing to hypoxanthine guanine phosphoribosyl transferase (HPRT) housekeeping gene. The fold-change of genes expression was calculated by using the comparative threshold method (ΔΔCt = difference of ΔCt between HA-treated cells and control) and the results were expressed as normalized fold expression compared to controls, calculated by using the Bio-Rad iQ^™^5 software (Bio-Rad Laboratories Srl), as the ratio of crossing points of amplification curves of several genes and internal standard [24].

**Table 1:** Primer sequences for specific biomarkers employed for the qRT-PCR.

Gene	Forward Primer	Reverse Primer	AT PCR
HPRT	5'-TGACCTTGATTTATTTTGCATACC-3'	5'-CGAGCAAGACGTTCAGTCCT-3'	55°C
COLIA1	5'-CCAGAAGAACTGGTACATCA-3'	5'-CCGCCATACTCGAACTGGAA-3'	55°C
COLIIIA1	5'-TGGTCCCCAAGGTGTCAAAG-3'	5'-GGGGGTCCTGGGTTACCATTA-3'	55°C
COLIVA1	5’-GGATCGGCTACTCTTTTGTGATG-3'	5'-AAGCGTTTGCGTAGTAATTGCA-3'	55°C
COLVIIA1	5'-CGGAACTGACCATCCAGAAT-3'	5'-AATAGGGTGCTCACGGTCAC-3'	55°C
Elastin	5'-AGGTGTATACCCAGGTGGCGTGCT-3'	5'-CAACCCCTGTCCCTGTTGGGTAAC-3'	60°C

### Western blotting analysis on cell monolayer

Western blot analyses were performed after seven days of treatment with H-HA, L-HA and H/L-HA hybrid complexes. Cells were lysed in RIPA buffer (Cell SignalingTechnology). Protein concentration was determined using the Bradford method [25] and 80 μg intracellular proteins were loaded and resolved using 10% SDS–PAGE. The separated proteins were then transferred to nitrocellulose membrane (Amersham). The membrane was blocked in 5% milk dissolved in Tris-buffered saline and 0.05% Tween-20. Elastin (60kDa) primary antibody was used at 1:250 dilutions. Immunoreactive bands were detected by chemiluminescence using corresponding horseradish peroxidase-conjugated secondary antibody (Santacruz Biotechnology; 1:5000 dilutions) and reacted withan ECL system (Chemicon-Millipore). Protein levels were normalized respect to the signal of anti actin polyclonal antibody using as housekeeping protein (Santacruz Biotechnology; 1:1000 dilutions). The semi-quantitative analysis of protein levels was carried out by the Gel Doc 2000 UV System and the Gel Doc EZ Imager (Quantity one software, Bio-Rad Laboratories).

### Immunofluorescence staining on cell monolayer

The expression of three ECM proteins in the skin, collagen type I, collagen type III and elastin, was evaluated on fixed HaCat/HDF co-cultures. After 24h and 7 days of incubation only for elastin antibody, cell co-cultures were stained simultaneously to ensure uniform conditions for fluorescence analyses, fixed in 4% paraformaldehyde for 15 min, rinsed in phosphate-buffered saline (PBS) and blocked by using blocking solution (1X PBS/5% normal serum/0.3% triton X-100) for 60 min. Cell co-cultures were then incubated overnight (4°C) with primary antibodies: type I collagen (monoclonal rabbit antibody, 1:100; Abcam, Cambridge, MA, USA), elastin (monoclonal mouse antibody, 1:50; Santa Cruz biotechnology, USA) and type III collagen (polyclonal mouse antibody, 1:100; Novus Biologicals, Cambridge UK). After three washings, glass microscope slides were incubated with Goat anti-Rabbit IgG (H+L) Secondary Antibody, Alexa Fluor^®^ 488 conjugate (1:100; Life Technologies Italy), goat anti-Mouse IgG (H+L) Secondary Antibody, Alexa Fluor^®^ 568 conjugate (1:100; Life Technologies Italy). To visualize actin filaments cells were stained with a 50 mg/ml fluorescent phalloidin TRITC conjugate (Sigma-Aldrich, Italy) solution in PBS for 40 minutes at room temperature. Nuclei were counterstained by Hoechst for 10 min (0.5μg/mL Sigma-Aldrich, Italy). Coverslip slides were obtained by using ProLong™ antifade mountant (Life Technologies Italy).

### Quantitative Real Time PCR (qRT-PCR) on FT-skin model

Total RNA was extracted from the FT-skin models using TRIzol^®^ (Invitrogen, Milan, Italy) and TissueRuptor homogenizer (Qiagen, Hilden Germany) according to the manufacturer’s procedures and c-DNA retrotrascription was performed using Reverse Transcription System Kit (Promega, Milan, Italy). PCR was then performed as described in the previous paragraph. In particular, the samples analysed were the 3D full Skin models injected: a) with PBS solution (CTR), b) with H/L-HA hybrid complexes, c) with H-HA and d) L-HA. The fold-change of genes expression was calculated by using the comparative threshold method (ΔΔCt = difference of ΔCt between HA-treated cells and negative control FT model injected with PBS solution) and the results were expressed as normalized fold expression with respect to the housekeeping gene, calculated by using the Bio-Rad iQ™5 software (Bio-Rad Laboratories Srl).

### Immunofluorescence staining of elastin on FT-skin model

After the incubation period (24h and 7 days), the FT-Skin models were immersed in formalin 4% v/v in PBS overnight.

The FT sections were appropriately dehydratated with increasing ethanol concentration (until 95%v/v), followed by isopropyl alcohol, then treated with xylene solvent overnight to be finally suitable for paraffin inclusion; the tissue *sections* were then cut with a microtome (10–20μm thick) in slices for the elastin antibody incubation.

For immunefluorescence steps, deparaffination and rehydration were performed, followed by 8 min at 99°C incubation in citrate buffer as antigen retrieval, then blocking with 5% BSA in PBS for 60 minutes followed by incubation with primary primary antibody: elastin (monoclonal mouse antibody, 1:50; Santa Cruz biotechnology, USA). After which, the sections were incubated with appropriate secondary antibody, Alexa Fluor 488-conjugated secondary antibody (Invitrogen) for 1h. To visualize actin filaments cells were stained with a 50 mg/ml fluorescent phalloidin TRITC conjugate (Sigma-Aldrich, Italy) solution in PBS for 40 minutes at room temperature. Nuclei were stained with Hoechst for 10 min (0.5μg/mL Sigma-Aldrich, Italy). Samples were then examined under the Nikon fluorescence microscope and analyzed by Nikon (Multicolor Package, Leica).

## Statistical Analysis

All experiments were performed in triplicate. Student’s t-test was used for statistical evaluation. The level of significance was set at p<0.01.

## Results

### Dynamic viscosity measurements

**Fig 1:**
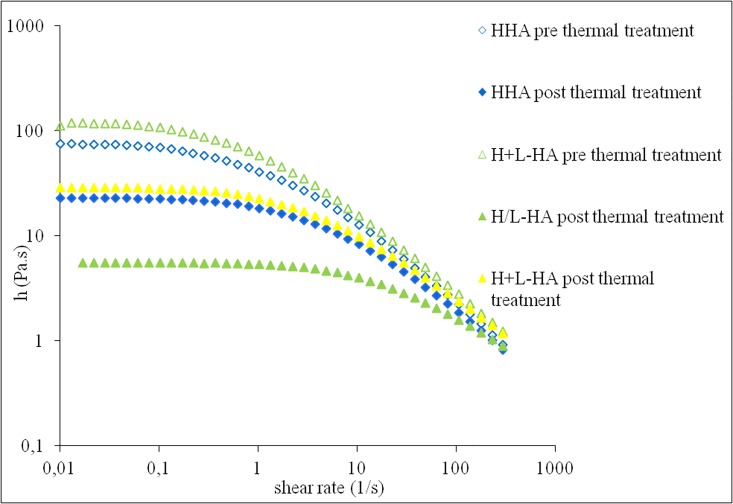
Flow curves (η vs shear rate) for H-HA pre and post thermal treatment (blue symbols void and solid, respectively), H+L-HA pre-thermal treatment (void green triangle), H/L-HA COMPLEXES hybrid cooperative complexes (green solid triangles), H+L-HA MIX: H-HA and L-HA separately thermally treated and then mixed after cooling (solid yellow symbols).

Rheology flow curves are displayed in Fig 1. In particular, the flow curves for H-HA (point 1 in the “rheology measurements” paragraph), H-HA+L-HA mixed pre or post thermal treatment (points 2 and 4, as above) and for H-HA/L-HA hybrid cooperative complexes (point 3, as above) are plotted.

The zero-shear viscosity (η_0_) values for the tested samples are reported in Table 2.

**Table 2:** Rheological values (η_0_) of H-HA (raw materials of PROFHILO devices); H+L-HA MIX and H/L-HA (COMPLEXES), pre- and post-thermal treatment (12 minutes at 120°C) and the corresponding fold decrease values.

η	η0 (Pa × sec) Pre thermal treatment	η0 (Pa × sec) Post thermal treatment	Fold decrease
**HHA (16mg/L)**	**74.4 ± 0.3**	**23.0 ± 0.2**	**3.2**
**H+L-HA MIX (16+16mg/L)**	**120.0 ± 0.5**	**28.3 ± 0.4**	**4.7**
**H/L-HA COMPLEX(16+16g/L)**	**120.0 ± 0.5**	**5.5 ± 0.0**	**21.9**

All samples showed a shear thinning behaviour, but with a constant η_0_ in the shear rate of 0,01 to 0,5 s^-1^. There was a marked decrease of dynamic viscosity, higher than 20 fold, related to the hybrid cooperative complexes formation (Table 2). Indeed, when the η_0_ of the sample 2 (mix of the H-HA chains and the low HA chains at room temperature), was compared to the η_0_ of the sample 4 (the two same solutions undergoing the thermal treatment, and mixed after), only a 4.7 fold viscosity decrease was observed while a 3.2-fold decrease in viscosity was observed with the sample 1 (H-HA).

The low η_0_ value for hybrid cooperative complexes give (confer) the product easier injectability (30 gauge needles are suggested), and expected better diffusibility within the tissue.

### Enzymatic stability

**Fig 2:**
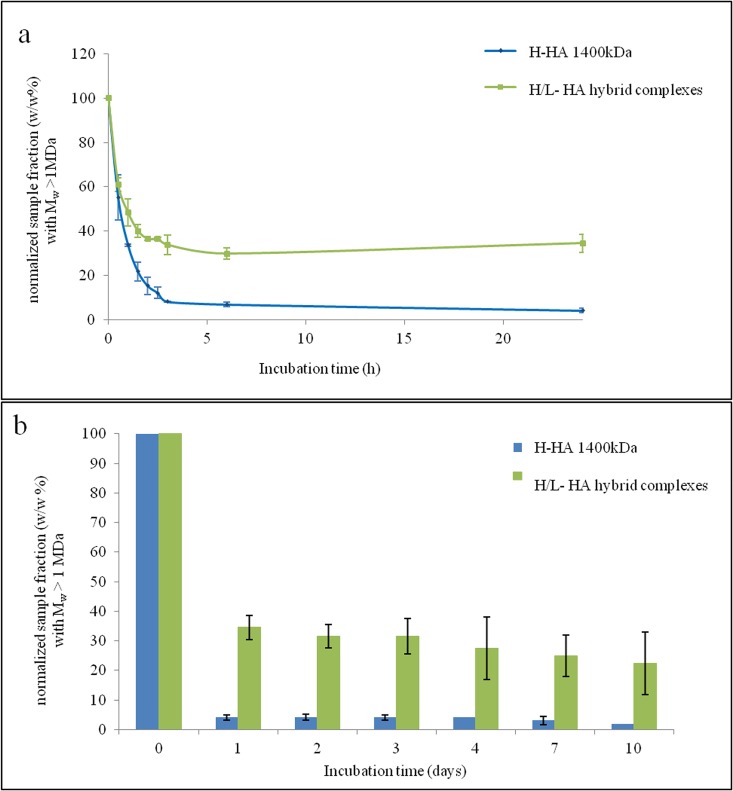
Results of sensitivity to enzymatic degradation for the hybrid cooperative complex and HHA: normalized sample fraction (w/w%) showing Mw higher than 1MDa during BTH 1U/mL action up to 24h (a) and 10days (b).

Results of enzymatic degradation studies are reported in Fig 2. The residual fraction (w/w %) with molecular weight higher than 1MDa, after incubation with BTH 1U/mL, was evaluated. In details, HA hybrid cooperative complexes and the linear H-HA were studied after 0.5h to 10 days of incubation with the enzyme (Fig 2A and 2B). Values were normalized for the initial composition at t = 0 (before the incubation). HA hybrid cooperative complexes showed a significantly higher resistance to BTH action than H-HA. After one day of incubation, BTH action reduced the residual fraction of the H-HA fraction (the HA hybrid cooperative complexes) to about 35% of the initial concentration and less than 10% of the H-HA. No further significant degradation could be detected in both samples when incubation lasted up to ten days.

### Analysis of collagens by qRT-PCR on cell monolayer

**Fig 3:**
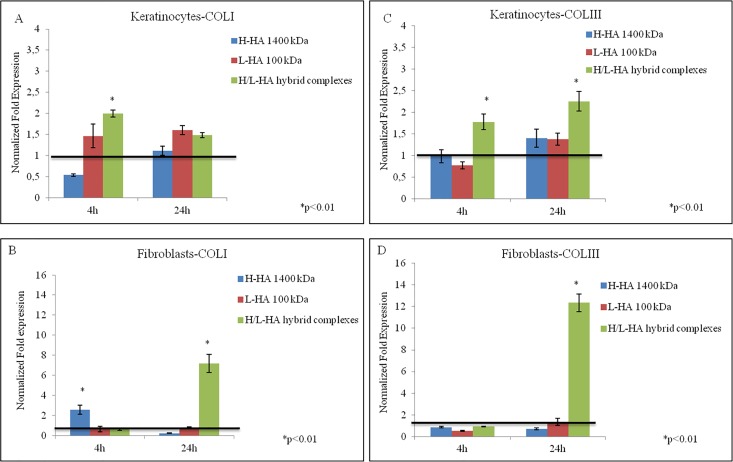
qRT-PCR relative to COLI and COLIII expression. Transcriptional analysis relative to COLIA1 and COLIIIA1 genes in (A-B) keratinocytes and (C-D) fibroblasts, in presence of H-HA, L-HA and H-HA/L-HA complexes 0.16% (w/w) after 4 and 24h. Values are calculated as mean ± SD of three different experiments and are expressed as mRNA normalized fold increase respect to CTR (1). Student’s t-test (*p<0.01).

Gene expression analyses about collagens (type I, III, IV, VII) and elastin were performed by quantitative real-time PCR (qRT-PCR), and the results were presented as normalized fold expression increase compared to control untreated cells and hypoxanthine guanine phosphoribosyl transferase (HPRT) housekeeping gene. Gene expression levels of type I and III collagen in both keratinocytes and fibroblasts cell cultures are shown in Fig 3. The expression levels of collagen I were two folds higher for the keratinocytes after 4 hours of incubation in presence of HA hybrid cooperative complexes compared to control cells and H- and L- HA-treated cells, which values were substantially similar to the control cells (half and one-and-half folds respectively) (Fig 3A). In fibroblasts, H-HA treatment prompted gene expression for collagen I (> two fold increase) at 4 hours, and, after 24 hours in presence of HA hybrid cooperative complexes, a seven folds higher gene expression for collagen-I was found compared to H-HA and L-HA treatments (Fig 3B). As for collagen III gene expression, in keratinocytes, after 24 hours in presence of HA hybrid cooperative complexes, an increase of two folds respect to H- and L- HA-treated cells was found (Fig 3C), while in fibroblasts this increase was twelve folds (Fig 3D).

**Fig 4:**
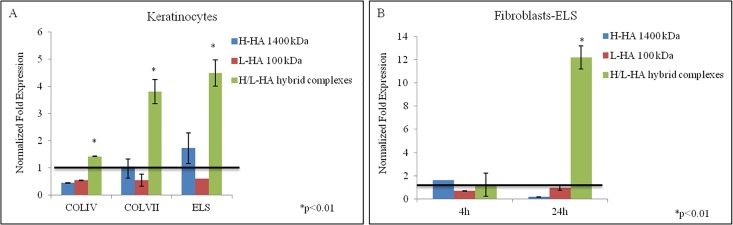
qRT-PCR relative to COLIV, COLVII and ELS expression. Transcriptional analysis relative to COLIVA1 and COLVIIA1 and ELS genes in (A) keratinocytes at 4h and (B-C) fibroblasts, in presence of H-HA, L-HA and H-HA/L-HA complexes 0.16% (w/w) at 4 and 24h. Values are the mean ± SD of three different experiments and are expressed as mRNA normalized fold increase respect to CTR (1). The samples (H-HA/L-HA complexes respect to H-HA) are significantly different in according to Student’s t-test (*p<0.01).

In Fig 4 the normalized mRNA expression levels of COLIV, COLVII, and ELS in keratinocytes and ELS in fibroblasts were reported. After 4 hours incubation, H/L-HA hybrid cooperative complexes significantly increased COLIV, COLVII, and ELS expression in keratinocytes (Fig 4A), while in fibroblasts COLIV and COLVII mRNA levels were slightly less or similar to control cells. After 24 hours in presence of HA hybrid cooperative complexes, ELS was significantly increased (twelve folds) (Fig 4B).

### Elastin Western blotting analysis

**Fig 5:**
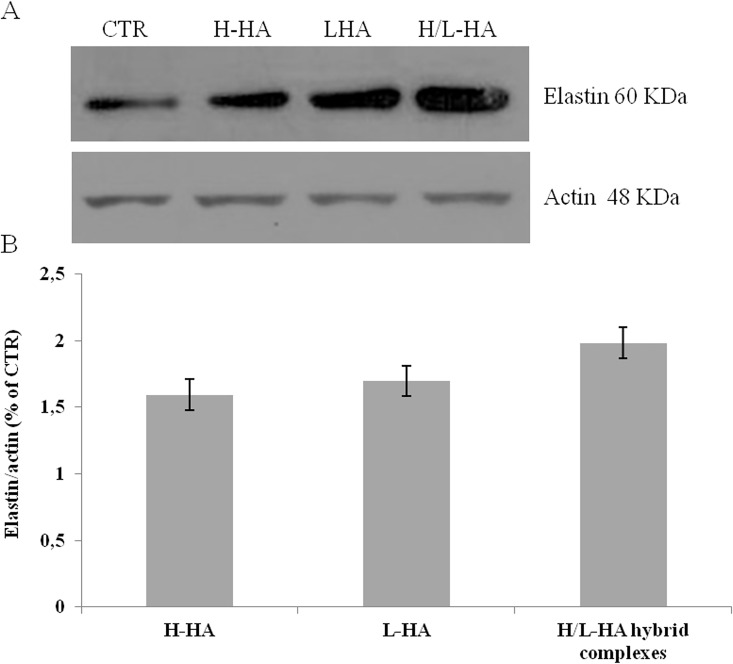
Western blot analysis of Elastin and Actin housekeeping protein levels in H-HA, L-HA and H/L-HA hybrid complexes treated samples, after 7 days of treatment, compared to the control. (A) Specific bands, corresponding to the proteins of interest are measured using commercially available software (Image J software). (B) Densitometry was reported as mean of two different experiments performed in duplicate.

As shown in Fig 5, western blotting analyses showed a significant elastin protein increase particularly in presence of H/L-HA hybrid complexes respect to H-HA and L-HA and respect to CTR.

### Immunofluorescence staining of collagens and elastin proteins on cell monolayer

**Fig 6:**
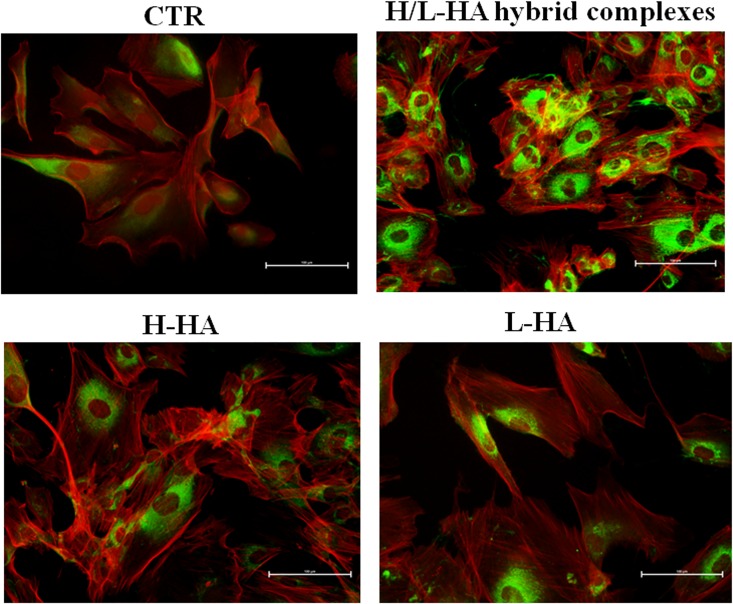
Keratinocytes-fibroblasts immunofluorescence pictures relative to COLIA1 expression in presence of H-HA, L-HA and H/L-HA complexes 0.16% (w/w) at 24h. Green: type I collagen, red: Cytoskeleton (Phalloidin).

**Fig 7:**
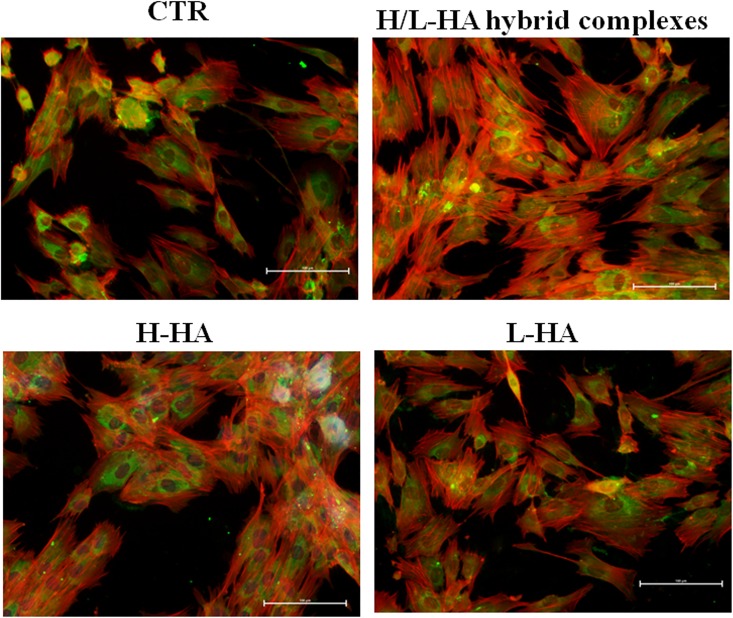
Keratinocytes-fibroblasts immunofluorescence pictures relative to COLIIIA1 expression in presence of H-HA, L-HA and H/L-HA complexes 0.16% (w/w) at 24h. Green: type III collagen, red: Cytoskeleton (Phalloidin).

**Fig 8:**
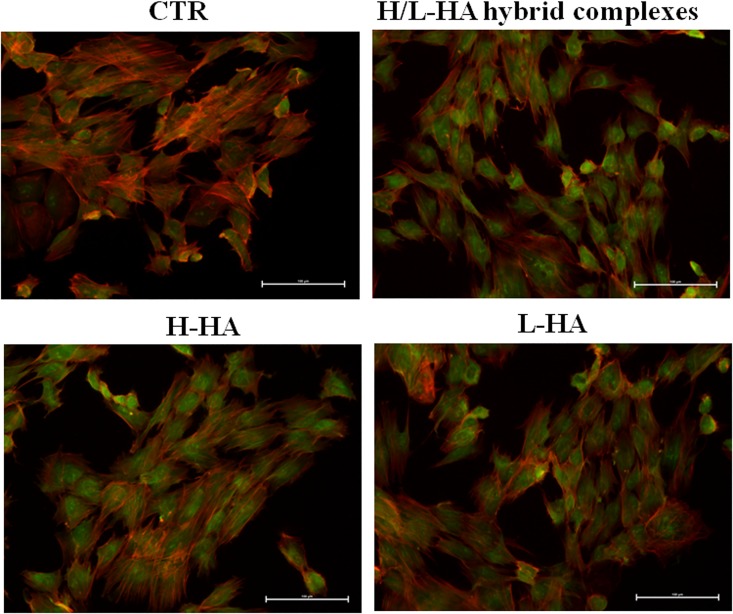
Keratinocytes-fibroblasts immunofluorescence pictures relative to ELS expression in presence of H-HA, L-HA and H/L-HA complexes 0.16% (w/w) at 24h. Green: ELS, red: Cytoskeleton (Phalloidin).

**Fig 9:**
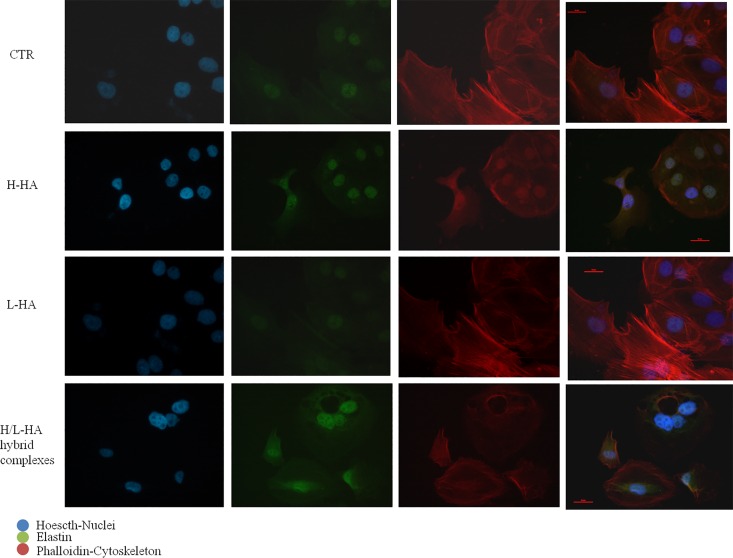
Keratinocytes-fibroblasts immunofluorescence pictures relative to ELS expression in presence of H-HA, L-HA and H/L-HA complexes 0.16% (w/w) at 7 days. Blue: Nuclei (Hoescth), Green: ELS, red: Cytoskeleton (Phalloidin).

At protein level, collagen (COLI and COLIII) and elastin (ELS) production were highlighted by immunofluorescence staining of keratinocytes and fibroblasts co-cultures. Microscope fluorescence observations showed that type I collagen protein synthesis was higher in fibroblasts than in keratinocytes (Fig 6). Also, the results suggested a different expression and localization of COLI when the cells were treated with HA hybrid cooperative complexes compared to H-HA and L-HA treatments. After 24h of incubation, the fluorescence intensity of COLIII was higher in presence of HA hybrid complexes, compared to control cells and H- and L- HA-treated cells (Fig 7). The images in Fig 8 displayed a higher elastin expression when keratinocyte-fibroblast co-cultures were treated with hyaluronan hybrid cooperative complexes than high and low molecular weight HA, respectively. Besides, in the Fig 9 the immunofluorescence staining of elastin after 7 days of incubation was reported. This data confirmed that HA hybrid complexes enhance elastin protein expression respect to CTR and H-HA and L-HA single treatment also at longer time.

### Analysis of collagens by qRT-PCR on FT-skin model

**Fig 10:**
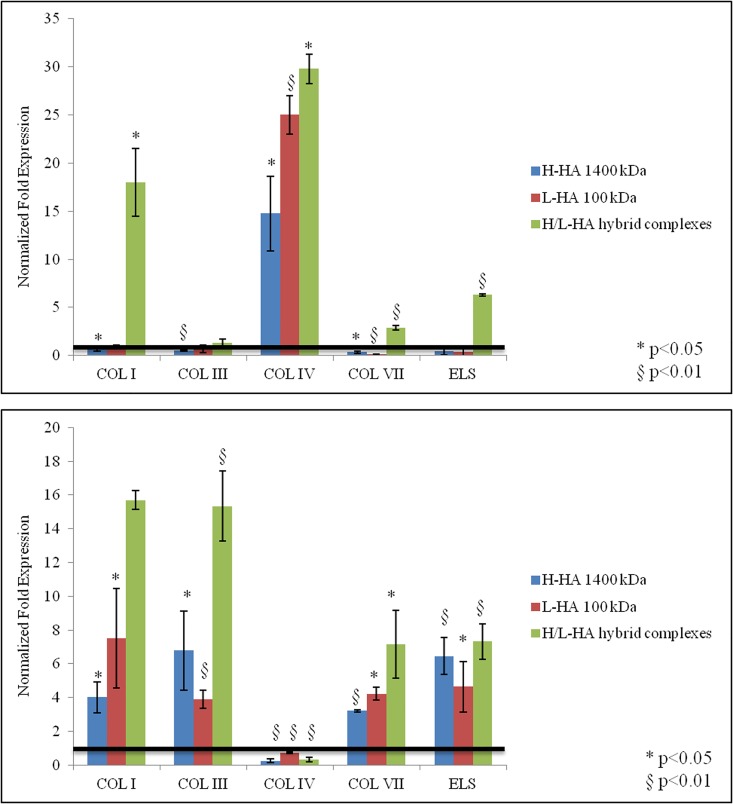
qRT-PCR analysis of COL I, COL III, COL IV, COL VII and elastin mRNA expression on FT skin model. Gene expression analysis in FT skin model was expressed as normalized fold expression respect to housekeeping gene and FT untreated model PBS injected (used as negative control), at 24h (A) and 7 days (B).

In the 3D skin model all the biomarkers already measured in the 2D *in vitro* experiments on HaCat and Fibroblasts, were evaluated after 24 h and 7 days of culture. As shown in Fig 10A and 10B, qRT-PCR proved a significantly higher expression of COLI, COL VII and elastin for the full thickness skin model samples treated with Hybrid complexes. The higher expression level was not only recorded respect to the control (injected PBS) but also respect to the samples treated with L-HA and H-HA. However for COL III and COL IV this expression was only slightly superior, even if still significant. Overall these data seem to be in agreement with our findings on the monolayer based human dermal cell model. In the 3D skin model at longer incubation time (7 days) COL I, COL III and COL VII expression was still slightly but significantly superior for hybrid complexes respect to H-HA and L-HA.

### Immunofluorescence staining of elastin on FT-skin model

**Fig 11:**
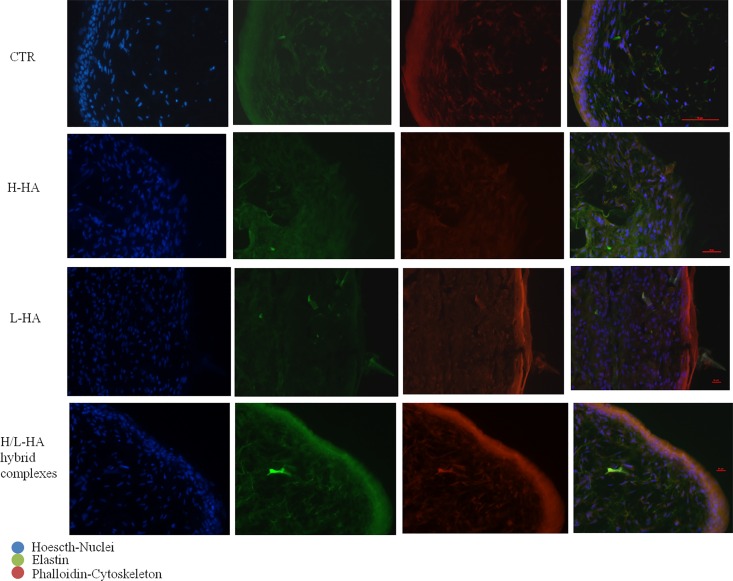
Immunofluorescence pictures relative to ELS expression on FT skin model in presence of H-HA, L-HA and H/L-HA complexes 0.16% (w/w) at 7 days. Blue: Nuclei (Hoescth), Green: ELS, red: Cytoskeleton (Phalloidin).

Immunofluorescence results for elastin on the 3D model for all the treatments and the control obtained are reported in Fig 11. As expected, elastin was expressed in all the FT-SKIN samples (CTR tissue injected with PBS and H-HA, L-HA and hybrid complexes). However, hybrid cooperative complexes treated samples showed a higher protein expression as evidenced by the higher intensity of the signal, respect to L-HA and H-HA treated samples respectively. In addition, for the 3D samples treated with hybrid complexes at 7 days a thicker epidermal layer was evident respect to the other sample images.

## Discussion

Because of HA pleiotropic effect, presence in various tissues, biocompatibility, and biodegradability, researchers worldwide have been studying HA-based formulations in the tissue engineering and regenerative medicine fields for years. Actually, due to its hydrophilicity and viscoelastic behaviour, HA is mainly used in cosmetic dermatology and skin care, and also in aesthetic medicine as dermal fillers and for biorevitalization procedures (intra/subdermal injections). Currently, several HA derived medical products have been developed and commercialized. A wide range of HA-based hydrogels is manufactured to provide minimally invasive aesthetic treatments [26]. Intradermal HA formulations are classified on the basis of their composition such as bio-fermentative or animal, cross-linked or not cross-linked, and even for the additional presence of other compounds (amino acids, vitamins, antioxidant compounds). Since 2006, HA dermal injections have been the most accepted noninvasive medical procedures to improve aged skin and for wrinkles correction and filling. As for this purpose, HA acts as a dermal expander filling lines, restoring volume, correcting other facial defects, and improving skin hydration. The major drawback of linear HA has been the *in vivo* short half-life, due to the rapid degradation by hyaluronidases enzymatic attack and also by free-radicals and other mechanisms (dilution, compression, etc…). For these reasons, HA derivates have been developed becoming widely used as fillers, their efficacy depending on some characteristics: concentration and size of the molecule, type, cross-linking degree, chemical and physical stability of the final product [27], [28, 29, 30]. However, undesired side effects were reported for products with high cross-linking degree, and/or due to residues of other chemical agents [9, 31]. The scientific community then has been designing novel HA-based formulations, some of these without chemical modification, to improve tolerability, permanence *in situ* and long-term effect. The development of a more stable slowly degraded product, without chemical modification, available as intradermal injectable formulations with enhanced injectability, longer duration, and high biocompatibility, has been accomplished with the patented (WO/2012/032151) NAHYCO^™^ technology [20]. This “more natural” HA product, commercially available as ProfHilo^®^, has been employed in this work and disclosed in the public and patent literature. The hybrid cooperative complexes deliver a higher HA amount than the unmodified HA products. Indeed, the hybrid cooperative complexes formation is characterized by a drop in dynamic viscosity that, in clinical practice, allows to deliver and inject very high concentrations of HA. As for the mechanism of the product development, the energy given through the thermal treatment is supposed to break the intra H-HA chains hydrogen bonds, leading to an entanglement of the H-HA and L-HA chains, having preferential cooperative bonding between each other during the cooling process. These peculiar linkages cannot be achieved making the thermal treatment separately on the H-HA and L-HA fractions and mixing the two after the cooling process. In addition, the thermal cycle can drive to hybrid cooperative complexes formation only if the molecular weights of hyaluronan are in a certain range and a certain ratio and, as described in the patent, are dissolved together before the thermal treatment.

The novel hybrid cooperative complexes formulations can be defined “physical gels”, in which interactions between long and short HA chains were made, without changing the disaccharides units structure and without introducing other “chemical compounds”.

Hybrid cooperative complexes showed more stability than the linear H-HA, even if they don't have chemical modifications or crosslinking, and indeed BTH hydrolysis was slower: after ten days of *in vitro* incubation, 35% of the initial high molecular weight fraction was still present in the samples, whereas, already at one day of incubation, the residual percentage of H-HA was below 10% (Fig 2B). Hybrid cooperative complexes seem to protect the high molecular weight hyaluronan from enzymatic degradation, and this fact is expected to give the product an *in vivo* longer persistence. It can be expected that the H-HA typical rheological properties, as well as the biological action (e.g. receptor interaction/biochemical cascade), were more persistent in this new preparation than linear HA formulations, and therefore HA hybrid cooperative complexes can represent a new and potential valuable alternative to dermal fillers commonly used in aesthetic medicine.

The possible alternative to hybrid cooperative complexes for prolonging performances without chemical modifications could be the injection of higher H-HA amount (> 16–20 mg/mL), but this choice is hampered by a very high viscosity and extrusion force needed. On the contrary, one of the advantages of the NAHYCO^™^ technology is the reduced viscosity of about seven folds compared to H-HA (Fig 1), and this characteristic makes possible to increase the injectable HA concentration (up to 64 mg/2ml) (with a needle of 29G). The injectable dermal formulations viscosity is indeed known to be a parameter critical for its biological and mechanical properties. However, beside the mechanical effects (e.g. scaffolding, plumping, hydration/swelling), the biological activity of these new formulations is a key point to be investigated. In this study, we used human keratinocyte/fibroblast co-cultures models to mimic better the *in vivo* condition and attempt to elucidate the biochemical/biological mechanism of action [32]. The possibility of comparing *in vitro* these hyaluronans, under standardized conditions not easy to reproduce *in vivo*, may give the chance to elucidate the mechanisms of action and suggest the basis for clinical practice. The biochemical pathways involved in the mechanism of action of HA may be very complicated for the interactions of diverse biomolecules, and in this study, because of their pivotal role in skin remodeling, we have chosen collagens and elastin as biomarkers [33, 34].

It is widely described that one of the factors affecting cutaneous aging is the depletion of endogenous HA in the skin extracellular matrix [35], and in the monocultures experiments performed in this study, after incubation with hybrid cooperative complexes, mRNA gene expression collagens and elastin showed a different modulation according to cell type. For example, type I and III collagens were expressed in keratinocytes cultures early (4 hours), compared to fibroblasts, where the expression levels of collagens were significantly increased after 24 hours instead. In addition, analysis of collagen types showed a marked expression of type IV and VII collagen in keratinocytes. These data made us hypothesize that, in different time intervals, fibroblasts and keratinocytes increase different ECM proteins synthesis (e.g., collagen I and III *vs*. Col IV and VII). Also, the cross-talk between keratinocytes and fibroblasts, which are distributed differently throughout the skin layers, could stimulate the synthesis and the rearrangement of the extracellular matrix [36]. In addition, in order to better evaluate and predict the efficacy of H/LHA hybrid complexes a full thickness skin model was used. Injected samples of this 3D skin model injected with H/LHA hybrid complexes prompted the early up-regulation of COLI and COL IV that suggest a matrix remodeling and a dermal regeneration [37]; while, the increase in COLI, COLIII, COL VII and ELS expression observed 7 days after the injection (Fig 10B) suggested that activation of the matrix remodeling was then starting to occur toward optimal skin tissue structure and homeostasis. Moreover elastin was followed by immunofluorescence analyses, green signal intensity is powered by hybrid complexes injection after 7 days of incubation, and also a better structure throughout the section and thickness of the epidermal outer layer was evidenced.

The data presented here indicate that this novel hybrid cooperative complex HA-based formulations clearly enhance collagen expression and elastin more than linear HA, contributing to refine cell morphology and potentially improving skin functions, elasticity and global tissue homeostasis. Quite notably, data about collagens and elastin expression were supported by data of protein synthesis: the immunofluorescence images (on monolayers) showed a higher increase of collagen I, III and elastin when cells were in presence of hybrid cooperative complexes than other HA formulations.

These data support the notion that the injection of HA-based formulations in the dermis could counteract physiological aging signs, leading to a sound biorevitalization effect.

## Conclusions

In this study, the multi-faceted interaction between keratinocytes and dermal fibroblasts in presence of the novel hybrid cooperative complexes HA formulation has been evaluated. The *in vitro* model employed has made possible the functional interaction between the two cell types, involving the synthesis and assembly of the skin ECM proteins. Also a full thickness skin model was used toward better resembling in vivo effect. The results showed a notably different biological response, regarding collagen and elastin expression and synthesis, of HA hybrid cooperative complexes respect to native HA formulations, with a potential for better global biorevitalization performance. A key feature of the hybrid cooperative complexes was the prolonged stability to the enzymatic attack, despite the absence of chemical cross linking. These findings could overall corroborate the *in vivo* clinical data obtained on the HA hybrid cooperative complex [38].

## Abbreviations

HA: Hyaluronic Acid
ECM: Extracellular Matrix
BTH: Bovine Testicular Hyaluronidase
H-HA: high molecular weight HA
L-HA: low molecular weight HA
H/L- HA: high/low HA hybrid cooperative complexes
qRT-PCR: quantitative real-time PCR
CD44: Cluster of Differentiation 44
TLR-4: Toll-like receptor 4
RHAMM: Hyaluronan-mediated motility receptor
ROS: Reactive oxygen species
SEC-TDA: Size Exclusion Chromatography-Triple Detector Array
PBS: phosphate buffered solution
HaCat: human dermal keratinocytes
HDF: human dermal fibroblasts
DMEM: Dulbecco’s Modified Eagle Medium
FBS: Fetal Bovine Serum
COLIA1: type I collagen
COLIIIA1: type III collagen
COLIVA1: type IV collagen
COLVIIA1: type VII collagen
ELS: elastin
HPRT: hypoxanthine guanine phosphoribosyl transferase
NAHYCO: NA (sodium) Hybrid Complex
