# Expression of Concern: Hyaluronan Hybrid Cooperative Complexes as a Novel Frontier for Cellular Bioprocesses Re-Activation

**DOI:** 10.1371/journal.pone.0302213

**Published:** 2024-04-10

**Authors:** The PLOS ONE Editors

After this article [1] was published, concerns were raised about Fig 9. Specifically, the CTR panels appear to partially overlap with the L-HA panels despite representing different experimental conditions.

The corresponding author stated this was caused by an error in the preparation of the figures and provided a corrected panel and the underlying data, included with this notice. The *PLOS ONE* Editors are satisfied that this concern has been resolved in full and that the conclusions of the article are not affected.

In reviewing this matter, it was noted that the underlying primary data were not provided with the published article, contrary to the Data Availability statement. The authors stated that the underlying quantitative data for Figs 5 and 10 were no longer available, but provided underlying data for all remaining figures. As such this article currently does not comply with the *PLOS ONE* Data Availability policy that was in place at the time of publication. The available data are provided with this notice.

In light of the above concern pertaining to data availability, the *PLOS ONE* Editors issue this Expression of Concern.

**Fig 9:**
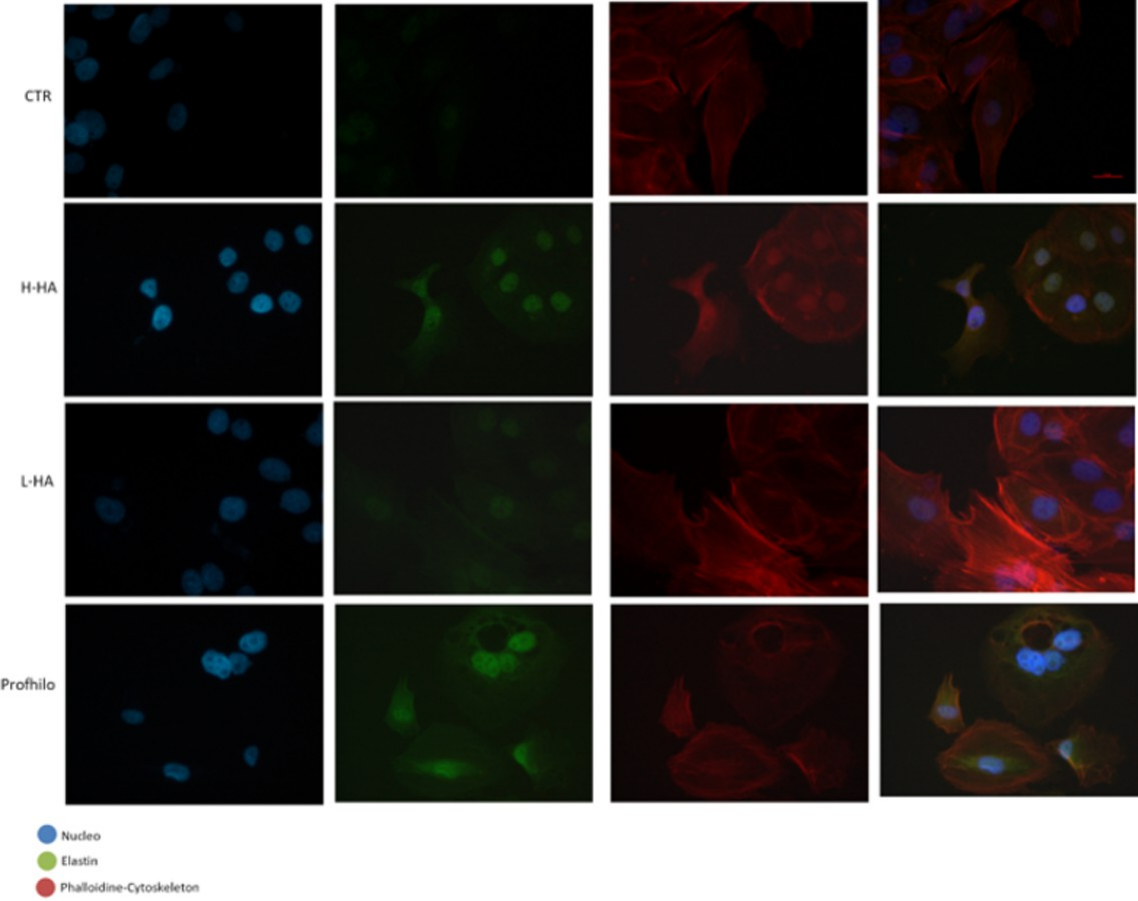
Keratinocytes-fibroblasts immunofluorescence pictures relative to ELS expression in presence of H-HA, L-HA and H/L-HA complexes 0.16% (w/w) at 7 days. Blue: Nuclei (Hoechst), Green: ELS, red: Cytoskeleton (Phalloidin).

## Supporting information

S1 File Underlying data for Figs 1–4 and 6. (ZIP)

S2 File Underlying data for Figs 7–9 and 11. (ZIP)
