# Supplementary material for: Expression of Concern: Hyaluronan Hybrid Cooperative Complexes as a Novel Frontier for Cellular Bioprocesses Re-Activation
Source: PLoS One. 2024 Apr 10;19(4):e0302213. doi: 10.1371/journal.pone.0302213 (PMC11006135; doi:10.1371/journal.pone.0302213)
Supplement: S1 File — (ZIP) [file pone.0302213.s001.zip › Copia di Copia di fig 3-4_response.pdf]

HPRT				
CTR	30,08291	29,01992	4h	29,55142
H-HA 1400 kDa	29,21938	28,86614		29,04276
H-HA 100 kDa	27,74918	27,78993		27,76955
H-HA/L-HA complex 0,16%	27,31171	27,12295		27,21733
CTR	26,93951	26,7065	24h	26,823
H-HA 1400 kDa	23,4108	23,05706		23,23393
H-HA 100 kDa	24,85209	24,39054		24,62132
H-HA/L-HA complex 0,16%	29,15874	29,42196		29,29035
CTR	22,78087	22,54338	4h	22,66213
H-HA 1400 kDa	21,88466	21,61823		21,75145
H-HA 100 kDa	20,92036	20,93109		20,92572
H-HA/L-HA complex 0,16%	22,54434	22,08828		22,31631

	Elastin			DCt	DCt
CTR	38,1475	38,1475	4h	8,5961	8,5961
H-HA 1400 kDa	36,9453	36,9322		7,9025	7,8894
L-HA 100 kDa	36,9453	36,8745		9,1757	9,1050
H-HA/L-HA complex 0,16%	35,7263	35,4983		8,5090	8,2810
CTR	37,7906	36,9322	24h	10,9676	10,1092
H-HA 1400 kDa	36,6405	36,0000		13,4066	12,7661
H-HA 100 kDa	35,3916	35,0146		10,7703	10,3933
H-HA/L-HA complex 0,16%	36,2166	35,4873		6,9263	6,1969
CTR	38,0000	37,8754	4h	15,3379	15,2133
H-HA 1400 kDa	36,0000	36,6747		14,2486	14,9233
H-HA 100 kDa	37,0000	37,0000		16,0743	16,0743
H-HA/L-HA complex 0,16%	35,6025	35,3240		13,2862	13,0077

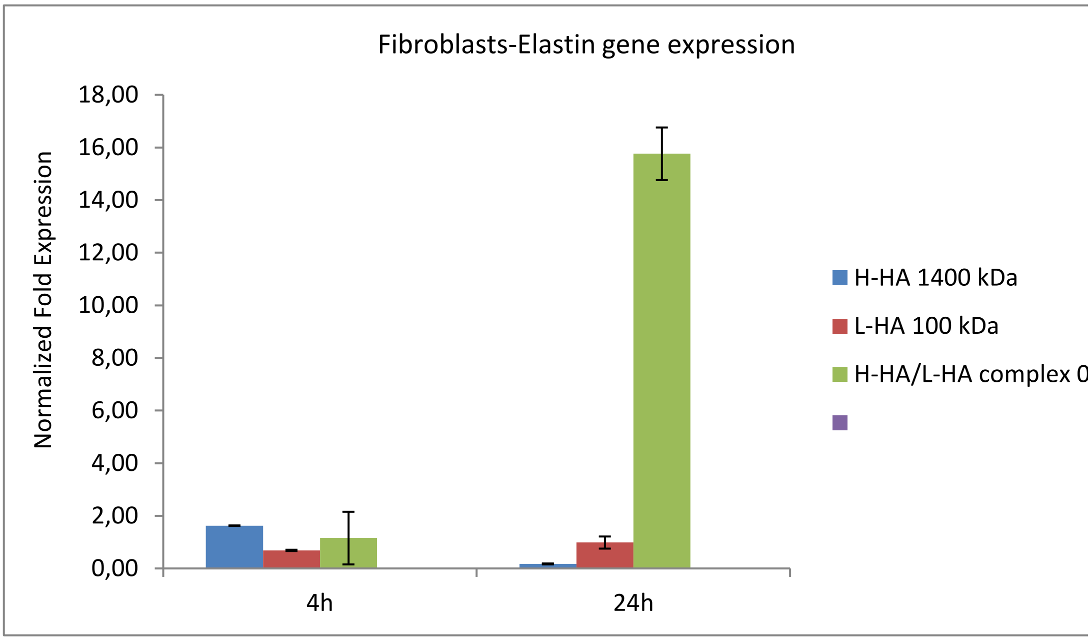

Fibroblasts-Elastin gene expression
Normalized Fold Expression
18,00
16,00
14,00
12,00
10,00
8,00
6,00
4,00
2,00
0,00
4h
24h
H-HA 1400 kDa
L-HA 100 kDa
H-HA/L-HA complex 0

0,751651
0,249778
0,028813
0,133475
0,164763
0,25013
0,326365
0,186125
0,167933
0,1884
0,007592
0,322484

DDCt	DDCt		
0,0000	0,0000	1,0000	1,0000
-0,6936	-0,7067	1,6173	1,6321
0,5796	0,5089	0,6691	0,7028
-0,0871	-0,3151	1,0623	1,2441
0,0000	0,0000	1,0000	1,0000
2,4390	2,6569	0,1844	0,1586
-0,1973	0,2841	1,1465	0,8213
-4,0413	-3,9123	16,4647	15,0561
0,0000	0,0000	1,0000	1,0000
-1,0893	-0,2900	2,1277	1,2226
0,7364	0,8610	0,6002	0,5506
-2,0517	-2,2055	4,1459	4,6125

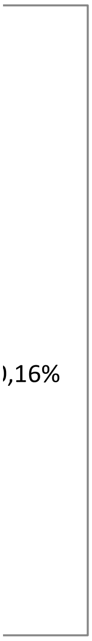
),16%

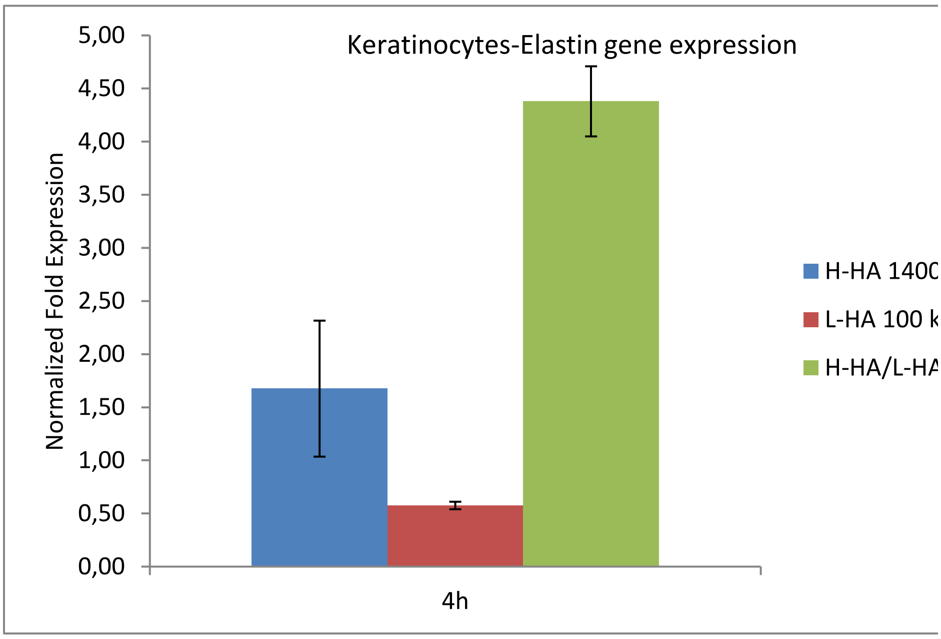
Keratinocytes-Elastin gene expression
Normalized Fold Expression
5,00
4,50
4,00
3,50
3,00
2,50
2,00
1,50
1,00
0,50
0,00
4h
H-HA 140C
L-HA 100 k
H-HA/L-HA

1,0000	0,0000
1,6247	0,0104
0,6860	0,0238
1,1532	0,1286
1,0000	0,0000
0,1715	0,0183
0,9839	0,2300
15,7604	0,9960
1,0000	0,0000
1,6752	0,6400
0,5754	0,0351
4,3792	0,3299

) kDa

kDa

A complex 0,16%
