# Supplementary material for: Expression of Concern: Hyaluronan Hybrid Cooperative Complexes as a Novel Frontier for Cellular Bioprocesses Re-Activation
Source: PLoS One. 2024 Apr 10;19(4):e0302213. doi: 10.1371/journal.pone.0302213 (PMC11006135; doi:10.1371/journal.pone.0302213)
Supplement: S1 File — (ZIP) [file pone.0302213.s001.zip › Copia di excel figure 2.a and 2.b_originalData.pdf]

Figure 2a

time (h)	H/L-HA normalized sample fraction (Mw higher than 1MDa)				
	repl 1	repl 2	repl 3	mean	SD
0	100	100	100	100	0
0,5	57,5	61	64	60,83333	3,253204
1	49	42	54,2	48,4	6,122091
1,5	37	41	42,5	40,16667	2,84312
2	36,1	35,6	36,84211	36,1807	0,624973
2,5	36	35,5	36,84211	36,11404	0,678281
3	32,8	29,5	38,7	33,66667	4,66083
6	27,5	29,2	32,3	29,66667	2,43379
24	32,8	39,3	31,5	34,53333	4,178915

#VALORE!

time (h)	H-HA normalized sample fraction (Mw higher than 1MDa)				
	repl 1	repl 2	repl 3	mean	SD
0	100	100	100	100	0
0,5	64,3	43,9	57,1	55,1	10,34601
1	33,7	33,4	34,4	33,83333	0,51316
1,5	23,5	17	25	21,83333	4,25245
2	18,5	11	16,8	15,43333	3,932345
2,5	14	9,6	13	12,2	2,306513
3	8	8	8,4	8,133333	0,23094
6	7,636364	5,9	7,3	6,945455	0,920878
24	5,2	4	3,4	4,2	0,916515

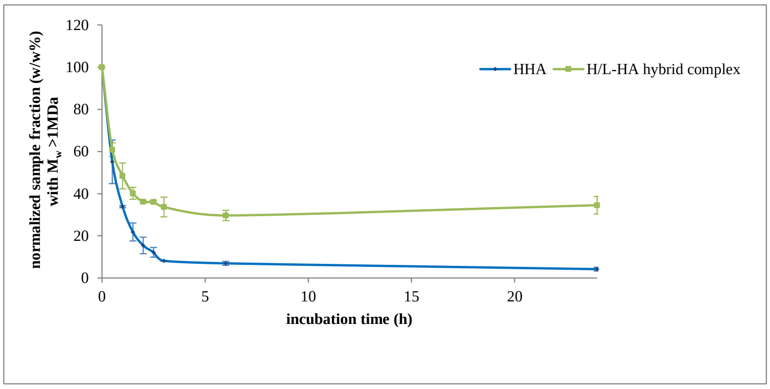

time (days)	H-HA normalized sample fraction (Mw higher than 1MDa)				
	repl 1	repl 2	repl 3	mean	SD
0	100	100	100	100	
1	4,9	4,4	3,2	4,166667	0,873689
2	4,9	4,5	3	4,133333	1,001665
3	3,2	5	4,3	4,166667	0,907377
4	4,15	4,05	4,1	4,1	0,05
7	2,2	2,2	4,7	3,033333	1,443376
10	2	2,040816	2,040816	2,027211	0,023565

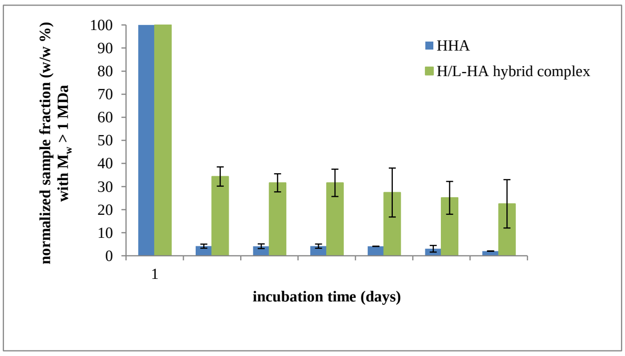

time (days)	H/L-HA normalized sample fraction (Mw higher than 1MDa)				
	repl 1	repl 2	repl 3	mean	SD
0	100	100	100	100	
1	39	33	31	34,33333	4,163332
2	27,5	32	35,3	31,6	3,915354
3	26	31	37,8	31,6	5,922837
4	36,6	15,8	29,8	27,4	10,60566
7	30,3	17	28	25,1	7,108446
10	32,6	11,7	23,2	22,5	10,46757
