# Supplementary material for: Expression of Concern: Hyaluronan Hybrid Cooperative Complexes as a Novel Frontier for Cellular Bioprocesses Re-Activation
Source: PLoS One. 2024 Apr 10;19(4):e0302213. doi: 10.1371/journal.pone.0302213 (PMC11006135; doi:10.1371/journal.pone.0302213)

HPRT					
CTR	30,08291	29,01992	4	29,55142	0,751651
H-HA 1400 kDa	29,21938	28,86614		29,04276	0,249778
H-HA 100 kDa	27,74918	27,78993		27,76955	0,028813
H-HA/L-HA complex 0,16%	27,31171	27,12295		27,21733	0,133475
CTR	26,93951	26,7065	24	26,823	0,164763
H-HA 1400 kDa	23,4108	23,05706		23,23393	0,25013
H-HA 100 kDa	24,85209	24,39054		24,62132	0,326365
H-HA/L-HA complex 0,16%	29,15874	29,42196		29,29035	0,186125
CTR	22,78087	22,54338	4	22,66213	0,167933
H-HA 1400 kDa	21,88466	21,61823		21,75145	0,1884
H-HA 100 kDa	20,92036	20,93109		20,92572	0,007592
H-HA/L-HA complex 0,16%	22,54434	22,08828		22,31631	0,322484
CTR	23,38505	23,21603	24	23,30054	0,119519
H-HA 1400 kDa	22,96168	22,23245		22,59706	0,515641
H-HA 100 kDa	22,61488	22,47154		22,54321	0,101356
H-HA/L-HA complex 0,16%	21,68659	21,80852		21,74756	0,086216

	TypeI collagen			DCt	DCt	DDCt	DDCt				
CTR	20,92181	20,42602	4h	-8,62961	-9,1254	0	0	1	1	1	0
H-HA 1400 kDa	18,89159	18,7374		-10,1512	-10,3054	-1,52157	-1,17996	2,871026	2,265706	2,568366	0,428026
L-HA 100 kDa	19,6776	19,4265		-8,0920	-8,3430	0,5376	0,7824	0,6889	0,5814	0,6352	0,0760
H-HA/L-HA complex 0,16%	19,0689	18,9456		-8,1484	-8,2717	0,4812	0,8537	0,7164	0,5534	0,6349	0,1153
CTR	19,1017	18,9046	24h	-7,7213	-7,9184	0,0000	0,0000	1,0000	1,0000	1,0000	0,0000
H-HA 1400 kDa	17,5613	17,5753		-5,6726	-5,6586	2,0487	2,2598	0,2417	0,2088	0,2253	0,0233
H-HA 100 kDa	17,2156	17,1515		-7,4058	-7,4698	0,3155	0,4486	0,8036	0,7328	0,7682	0,0501
H-HA/L-HA complex 0,16%	18,6056	18,6600		-10,6848	-10,6303	-2,9635	-2,7119	7,8003	6,5517	7,1760	0,8829
CTR	30,1566	29,9434	4,0000	7,4945	7,2813	0,0000	0,0000	1,0000	1,0000	1,0000	0,0000
H-HA 1400 kDa	30,0801	29,9898		8,3286	8,2384	0,8342	0,9571	0,5609	0,5151	0,5380	0,0324
H-HA 100 kDa	27,6853	27,8686		6,7596	6,9429	-0,7349	-0,3383	1,6643	1,2643	1,4643	0,2828
H-HA/L-HA complex 0,16%	28,7694	28,6461		6,4531	6,3298	-1,0413	-0,9514	2,0581	1,9338	1,9959	0,0879
CTR	26,9916	26,9826	24,0000	3,6910	3,6821	0,0000	0,0000	1,0000	1,0000	1,0000	0,0000
H-HA 1400 kDa	26,2342	26,0327		3,6372	3,4356	-0,0539	-0,2464	1,0380	1,1863	1,1122	0,1048
H-HA 100 kDa	25,4884	25,6161		2,9452	3,0729	-0,7459	-0,6091	1,6770	1,5253	1,6012	0,1072
H-HA/L-HA complex 0,16%	24,9076	24,8152		3,1600	3,0676	-0,5310	-0,6144	1,4450	1,5309	1,4880	0,0608

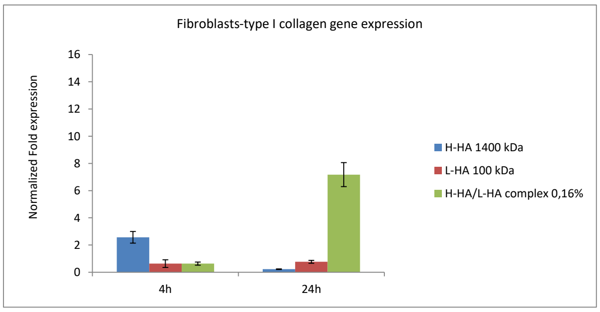

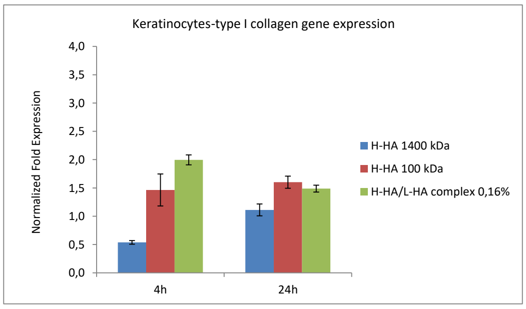

Supplement: S1 File — (ZIP) [file pone.0302213.s001.zip › fig 3-4_response_25_3_24_colI.pdf]
