# Supplementary material for: Expression of Concern: Hyaluronan Hybrid Cooperative Complexes as a Novel Frontier for Cellular Bioprocesses Re-Activation
Source: PLoS One. 2024 Apr 10;19(4):e0302213. doi: 10.1371/journal.pone.0302213 (PMC11006135; doi:10.1371/journal.pone.0302213)

HPRT					
CTR	30,0829	29,0199	4,0000	29,5514	0,7517
H-HA 1400 kDa	29,2194	28,8661		29,0428	0,2498
H-HA 100 kDa	27,7492	27,7899		27,7696	0,0288
H-HA/L-HA complex 0,16%	27,3117	27,1229		27,2173	0,1335
CTR	26,9395	26,7065	24,0000	26,8230	0,1648
H-HA 1400 kDa	23,4108	23,0571		23,2339	0,2501
H-HA 100 kDa	24,8521	24,3905		24,6213	0,3264
H-HA/L-HA complex 0,16%	28,8736	28,4154		28,6445	0,3240
CTR	22,6509	22,5434	4,0000	22,5971	0,0760
H-HA 1400 kDa	21,8847	21,6182		21,7514	0,1884
L-HA 100 kDa	20,9204	20,9311		20,9257	0,0076
H-HA/L-HA complex 0,16%	22,4443	22,4134		22,4289	0,0219
CTR	23,3851	23,2160	24,0000	23,3005	0,1195
H-HA 1400 kDa	22,9617	22,2324		22,5971	0,5156
L-HA 100 kDa	22,6149	22,4715		22,5432	0,1014
H-HA/L-HA complex 0,16%	21,6866	21,8085		21,7476	0,0862

	TypeIII collagen			DCt	DCt	DDCt	DDCt				
CTR	28,5916	28,1633	4h	-0,9599	-1,3881	0,0000	0,0000	1,0000	1,0000	1,0000	0,0000
H-HA 1400 kDa	28,2094	27,9695		-0,8333	-1,0733	0,1265	0,3148	0,9160	0,8040	0,8600	0,0793
L-HA 100 kDa	27,7258	27,4603		-0,0438	-0,3092	0,9161	1,0788	0,5299	0,4734	0,5017	0,0400
H-HA/L-HA complex 0,16%	26,4037	25,9217		-0,8136	-1,2956	0,1463	0,0925	0,9036	0,9379	0,9208	0,0243
CTR	26,3094	26,3940	24h	-0,5136	-0,4290	0,0000	0,0000	1,0000	1,0000	1,0000	0,0000
H-HA 1400 kDa	23,3761	23,1761		0,1421	-0,0578	0,6558	0,3711	0,6347	0,7732	0,7040	0,0979
L-HA 100 kDa	23,9370	23,5223		-0,6844	-1,0991	-0,1707	-0,6701	1,1256	1,5912	1,3584	0,3292
H-HA/L-HA complex 0,16%	24,6102	24,5734		-4,0343	-4,0711	-3,5207	-3,6422	11,4769	12,4855	11,9812	0,7132
CTR	35,4631	35,9236	4,0000	12,8660	13,3265	0,0000	0,0000	1,0000	1,0000	1,0000	0,0000
H-HA 1400 kDa	34,8520	34,9230		13,1006	13,1716	0,2346	-0,1549	0,8499	1,1134	0,9817	0,1863
L-HA 100 kDa	34,1710	35,0204		13,2453	14,0947	0,3793	0,7682	0,7688	0,5872	0,6780	0,1285
H-HA/L-HA complex 0,16%	34,6910	34,8372		12,2621	12,4083	-0,6039	-0,9182	1,5198	1,8897	1,7047	0,2616
CTR	31,6857	31,5215	24,0000	8,3852	8,2210	0,0000	0,0000	1,0000	1,0000	1,0000	0,0000
H-HA 1400 kDa	30,4138	30,4678		7,8167	7,8708	-0,5685	-0,3502	1,4830	1,2748	1,3789	0,1472
L-HA 100 kDa	30,4516	30,4544		7,9083	7,9112	-0,4769	-0,3098	1,3917	1,2395	1,3156	0,1076
H-HA/L-HA complex 0,16%	29,0818	28,6809		7,3342	6,9334	-1,0510	-1,2876	2,0719	2,4413	2,2566	0,2612

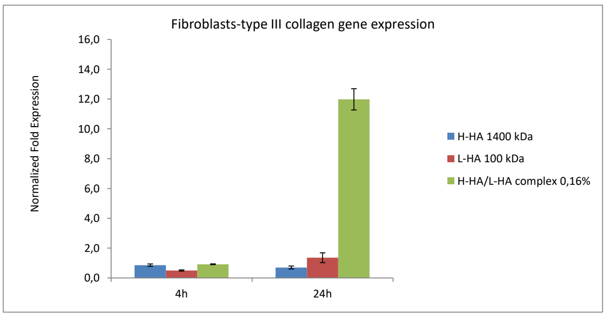

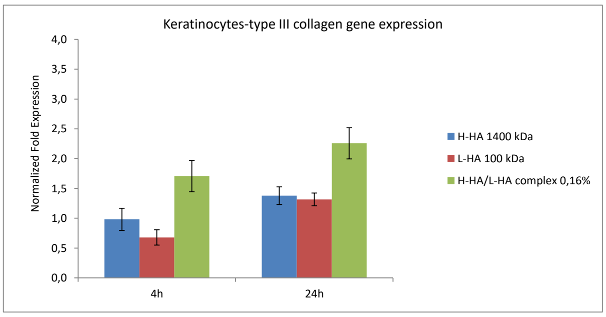

Supplement: S1 File — (ZIP) [file pone.0302213.s001.zip › fig 3-4_response_25_3_24_colIII.pdf]
