# Supplementary material for: Expression of Concern: Hyaluronan Hybrid Cooperative Complexes as a Novel Frontier for Cellular Bioprocesses Re-Activation
Source: PLoS One. 2024 Apr 10;19(4):e0302213. doi: 10.1371/journal.pone.0302213 (PMC11006135; doi:10.1371/journal.pone.0302213)

HPRT

| | | | | | |
|---|---|---|---|---|---|
| CTR | 22,7809 | 22,5434 | 4,0000 | 22,6621 | 0,1679 |
| H-HA 1400 kDa | 21,8847 | 21,6182 | | 21,7514 | 0,1884 |
| H-HA 100 kDa | 20,9204 | 20,9311 | | 20,9257 | 0,0076 |
| H-HA/L-HA complex 0,16% | 22,5443 | 22,0883 | | 22,3163 | 0,3225 |

| | TypeVII collagen | | | DCt | DCt | DDCt | DDCt | | | | |
|---|---|---|---|---|---|---|---|---|---|---|---|
| | | | | | | | | | | MEDIA | DEV.ST |
| CTR | 32,3941 | 32,2040 | 4,0000 | 9,7319 | 9,5419 | 0,0000 | 0,0000 | 1,0000 | 1,0000 | 1,0000 | 0,0000 |
| H-HA 1400 kDa | 31,2009 | 31,7667 | | 9,4495 | 10,0153 | -0,2825 | 0,4734 | 1,2163 | 0,7203 | 0,9683 | 0,3507 |
| L-HA 100 kDa | 31,1556 | 31,8301 | | 10,2298 | 10,9044 | 0,4979 | 1,3625 | 0,7081 | 0,3889 | 0,5485 | 0,2257 |
| H-HA/L-HA complex 0,16% | 30,0029 | 30,0522 | | 7,6866 | 7,7359 | -2,0453 | -1,8060 | 4,1276 | 3,4968 | 3,8122 | 0,4461 |

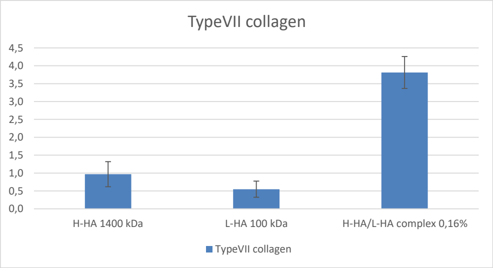

Supplement: S1 File — (ZIP) [file pone.0302213.s001.zip › fig 3-4_response_25_3_24_colVII.pdf]
