# Supplementary material for: Expression of Concern: Hyaluronan Hybrid Cooperative Complexes as a Novel Frontier for Cellular Bioprocesses Re-Activation
Source: PLoS One. 2024 Apr 10;19(4):e0302213. doi: 10.1371/journal.pone.0302213 (PMC11006135; doi:10.1371/journal.pone.0302213)

HPRT					
CTR	30,08291	29,01992	4h	29,55142	0,751651
H-HA 1400 kDa	29,21938	28,86614		29,04276	0,249778
H-HA 100 kDa	27,74918	27,78993		27,76955	0,028813
H-HA/L-HA complex 0,16%	27,31171	27,12295		27,21733	0,133475
CTR	26,93951	26,7065	24h	26,823	0,164763
H-HA 1400 kDa	23,4108	23,05706		23,23393	0,25013
H-HA 100 kDa	24,85209	24,39054		24,62132	0,326365
H-HA/L-HA complex 0,16%	29,15874	29,42196		29,29035	0,186125
CTR	22,78087	22,54338	4h	22,66213	0,167933
H-HA 1400 kDa	21,88466	21,61823		21,75145	0,1884
H-HA 100 kDa	20,92036	20,93109		20,92572	0,007592
H-HA/L-HA complex 0,16%	22,54434	22,08828		22,31631	0,322484

	Elastin			DCt	DCt	DDCt	DDCt				
CTR	38,1475	38,1475	4h	8,5961	8,5961	0,0000	0,0000	1,0000	1,0000	1,0000	0,0000
H-HA 1400 kDa	36,9453	36,9322		7,9025	7,8894	-0,6936	-0,7067	1,6173	1,6321	1,6247	0,0104
L-HA 100 kDa	36,9453	36,8745		9,1757	9,1050	0,5796	0,5089	0,6691	0,7028	0,6860	0,0238
H-HA/L-HA complex 0,16%	35,7263	35,4983		8,5090	8,2810	-0,0871	-0,3151	1,0623	1,2441	1,1532	0,1286
CTR	37,7906	36,9322	24h	10,9676	10,1092	0,0000	0,0000	1,0000	1,0000	1,0000	0,0000
H-HA 1400 kDa	36,6405	36,0000		13,4066	12,7661	2,4390	2,6569	0,1844	0,1586	0,1715	0,0183
H-HA 100 kDa	35,3916	35,0146		10,7703	10,3933	-0,1973	0,2841	1,1465	0,8213	0,9839	0,2300
H-HA/L-HA complex 0,16%	36,8166	35,7267		7,5263	6,4363	-3,4413	-3,6729	10,8626	12,7539	11,8083	1,3373
CTR	38,0000	37,8754	4h	15,3379	15,2133	0,0000	0,0000	1,0000	1,0000	1,0000	0,0000
H-HA 1400 kDa	36,0000	36,6747		14,2486	14,9233	-1,0893	-0,2900	2,1277	1,2226	1,6752	0,6400
H-HA 100 kDa	37,0000	37,0000		16,0743	16,0743	0,7364	0,8610	0,6002	0,5506	0,5754	0,0351
H-HA/L-HA complex 0,16%	35,6025	35,3240		13,2862	13,0077	-2,0517	-2,2055	4,1459	4,6125	4,3792	0,3299

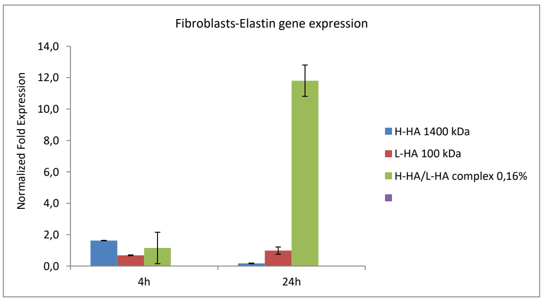

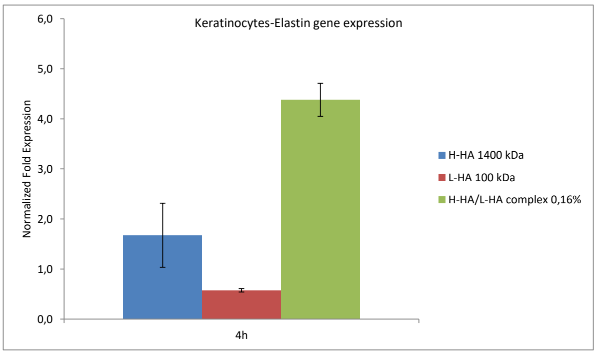

Supplement: S1 File — (ZIP) [file pone.0302213.s001.zip › fig 3-4_response_25_3_24_el.pdf]
