# Supplementary material for: Expression of Concern: Hyaluronan Hybrid Cooperative Complexes as a Novel Frontier for Cellular Bioprocesses Re-Activation
Source: PLoS One. 2024 Apr 10;19(4):e0302213. doi: 10.1371/journal.pone.0302213 (PMC11006135; doi:10.1371/journal.pone.0302213)
Supplement: S1 File — (ZIP) [file pone.0302213.s001.zip › figura 1_rawdata_rheology25_3_24.pdf]

shear rate (1/s)	H+L-HA pre thermal treatment	HHA pre-thermal treatment	H+L-HA post thermal treatment	HHA post thermal treatment	H/L-HA post thermal treatment
0,0101	112	74,8	28,7	23	
0,0131	119	74,7	28,7	23	
0,017	119	74,5	28,7	22,9	5,49
0,0219	118	74,3	28,6	22,8	5,48
0,0284	118	74,1	28,6	22,8	5,49
0,0367	117	73,6	28,6	22,7	5,49
0,0475	115	72,9	28,5	22,7	5,5
0,0614	113	72	28,5	22,6	5,5
0,0795	110	70,8	28,4	22,5	5,5
0,103	107	69,1	28	22,4	5,5
0,133	103	66,9	27,9	22,2	5,5
0,172	98,3	64,3	27,7	22	5,49
0,222	93,4	61,4	27,3	21,8	5,48
0,288	87,9	58,2	26,9	21,4	5,45
0,372	82,2	54,8	26,3	21	5,48
0,482	76,2	51,4	25,5	20,5	5,45
0,623	70	47,9	24,7	19,9	5,41
0,806	63,8	44,3	23,6	19,1	5,38
1,04	57,6	40,7	22,5	18,3	5,34
1,35	51,5	37,2	21,2	17,3	5,29
1,74	45,6	33,6	19,8	16,3	5,21
2,26	40	30,2	18,4	15,2	5,1
2,92	34,8	26,8	16,9	14	4,98
3,77	30	23,5	15,4	12,9	4,82
4,88	25,6	20,5	14	11,7	4,64
6,32	21,7	17,6	12,5	10,5	4,44
8,17	18,3	15	11,1	9,35	4,21
10,6	15,4	12,7	9,79	8,25	3,95
13,7	12,8	10,7	8,56	7,21	3,69
17,7	10,7	8,86	7,42	6,24	3,41
22,9	8,87	7,73	6,4	5,35	3,13
29,6	7,34	6,03	5,48	4,56	2,84
38,3	6,06	4,96	4,68	3,85	2,56
49,5	5	4,07	3,97	3,23	2,29
64	4,12	3,33	3,35	2,69	2,04
82,8	3,39	2,73	2,82	2,23	1,8
107	2,77	1,84	2,37	1,84	1,58
139	2,25	1,8	1,99	1,51	1,38
179	1,83	1,45	1,66	1,24	1,19
232	1,5	1,14	1,39	1,01	1,03
300	1,23	0,922	1,16	0,82	0,89
