# Supplementary figures and images for: Expression of Concern: Hyaluronan Hybrid Cooperative Complexes as a Novel Frontier for Cellular Bioprocesses Re-Activation
Source: PLoS One. 2024 Apr 10;19(4):e0302213. doi: 10.1371/journal.pone.0302213 (PMC11006135; doi:10.1371/journal.pone.0302213)

|                         |         |         |        |         |        |
|-------------------------|---------|---------|--------|---------|--------|
| CTR                     | 22,7809 | 22,5434 | 4,0000 | 22,6621 | 0,1679 |
| H-HA 1400 kDa           | 21,8847 | 21,6182 |        | 21,7514 | 0,1884 |
| H-HA 100 kDa            | 20,9204 | 20,9311 |        | 20,9257 | 0,0076 |
| H-HA/L-HA complex 0,16% | 22,5443 | 22,0883 |        | 22,3163 | 0,3225 |

|                         |         |         |        |        |        |         |         |        |        |        |        |
|-------------------------|---------|---------|--------|--------|--------|---------|---------|--------|--------|--------|--------|
| CTR                     | 25,6737 | 25,6279 | 4,0000 | 3,0116 | 2,9658 | 0,0000  | 0,0000  | 1,0000 | 1,0000 | 1,0000 | 0,0000 |
| H-HA 1400 kDa           | 25,9195 | 25,8392 |        | 4,1681 | 4,0877 | 1,1565  | 1,1219  | 0,4486 | 0,4595 | 0,5440 | 0,0077 |
| H-HA 100 kDa            | 24,8256 | 24,7581 |        | 3,8999 | 3,8324 | 0,8884  | 0,8666  | 0,5402 | 0,5484 | 0,5443 | 0,0058 |
| H-HA/L-HA complex 0,16% | 24,8256 | 24,7581 |        | 2,5093 | 2,4418 | -0,5022 | -0,5240 | 1,4164 | 1,4379 | 1,4272 | 0,0152 |

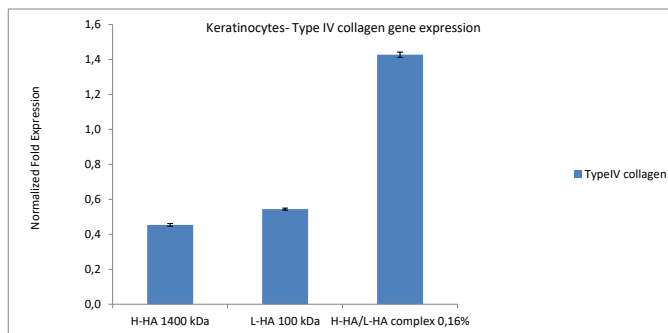

Supplement: S1 File — (ZIP) [file pone.0302213.s001.zip › fig 3-4_response_25_3_24_colIV.pdf]

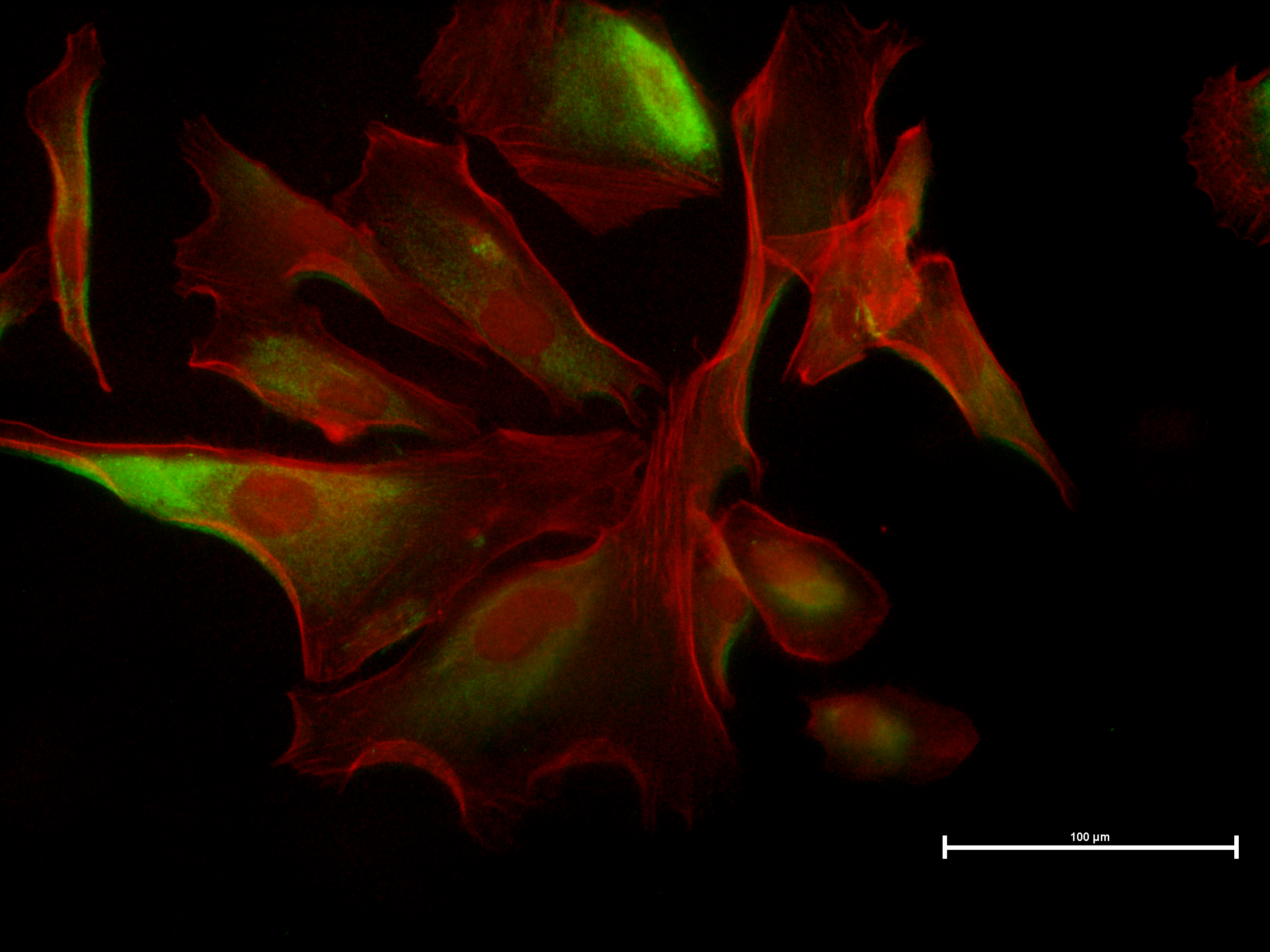

Supplement: S1 File — (ZIP) [file pone.0302213.s001.zip › FIGURA6/CTR.tif]

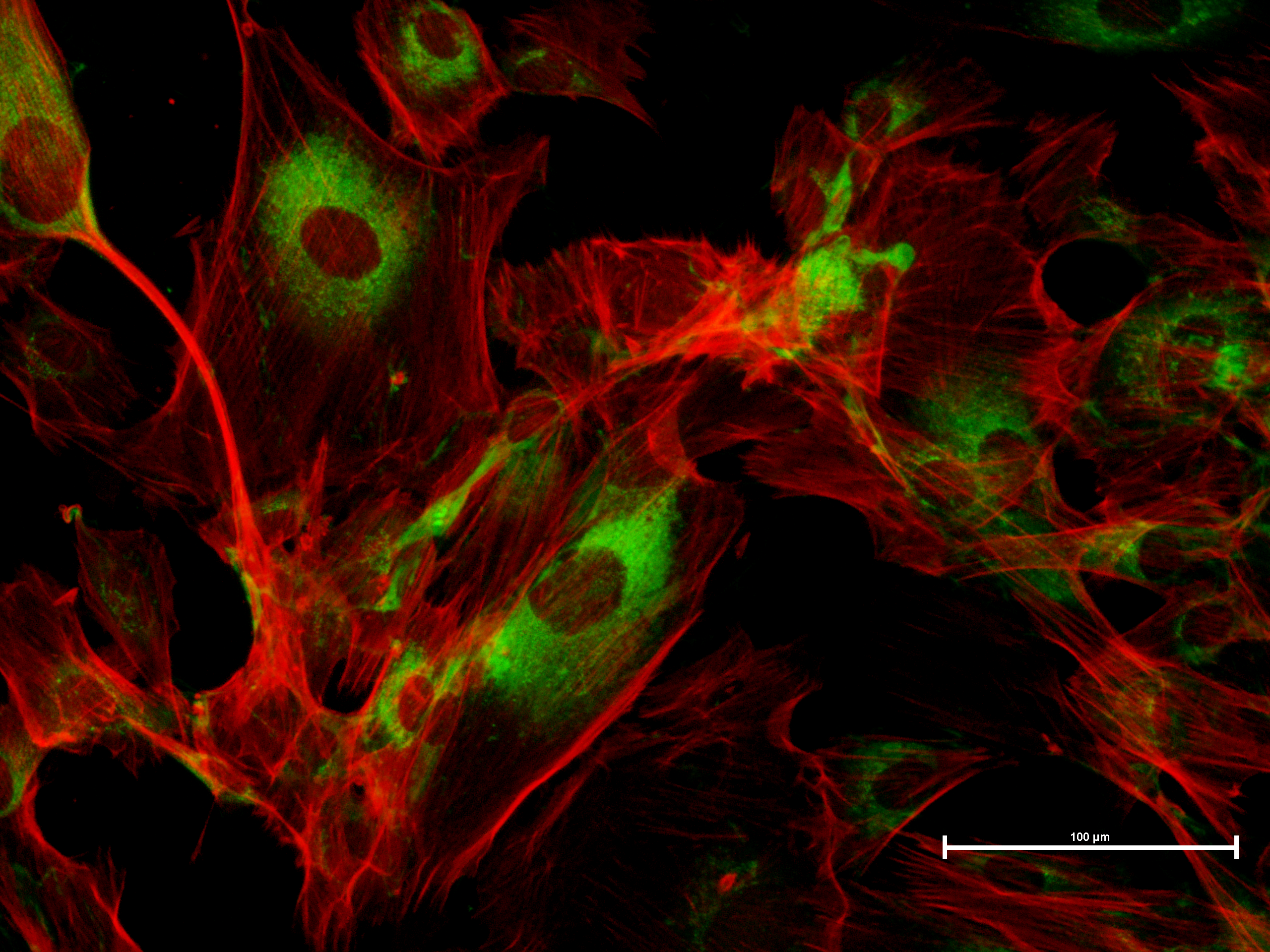

Supplement: S1 File — (ZIP) [file pone.0302213.s001.zip › FIGURA6/HHA.tif]

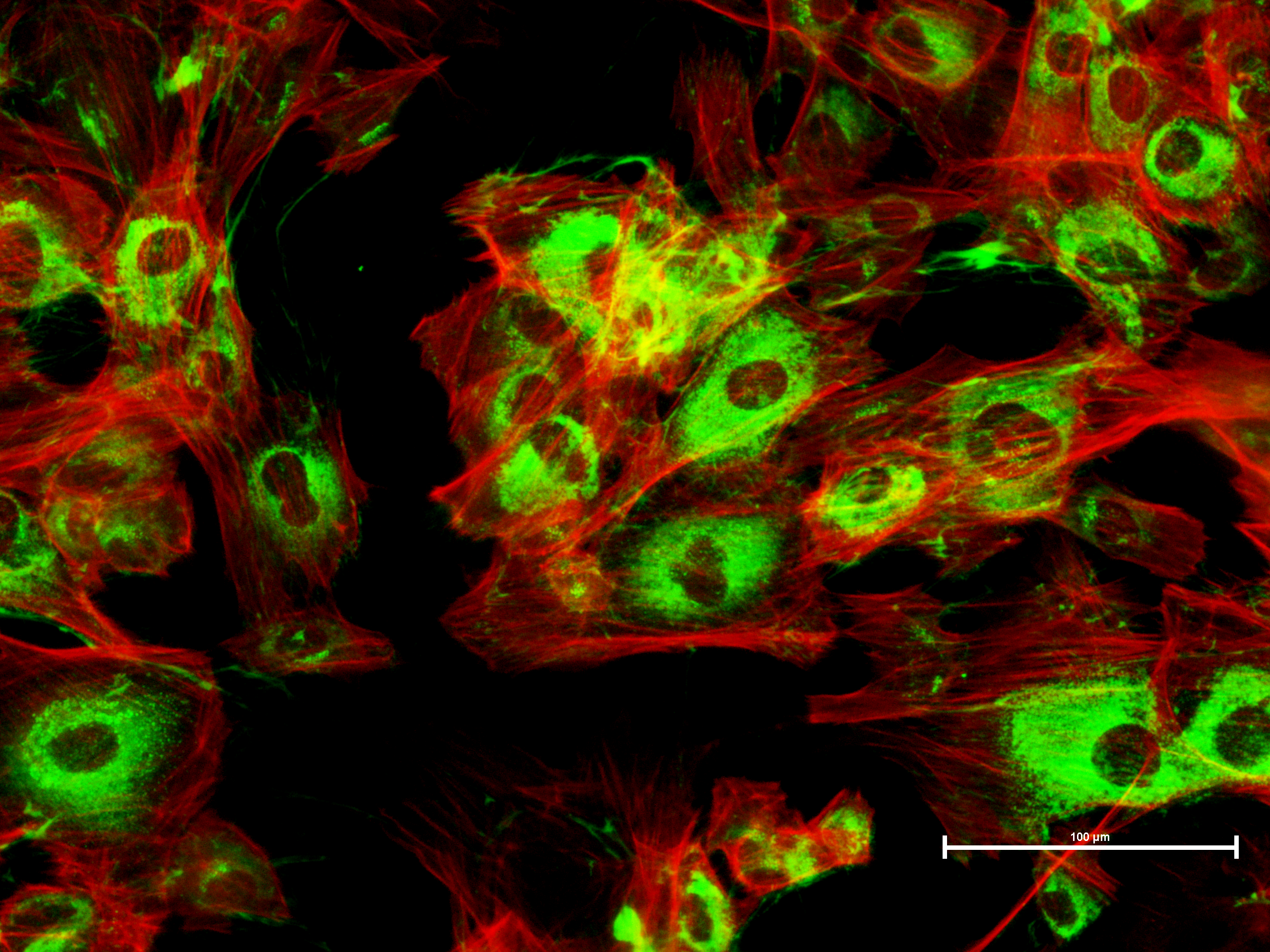

Supplement: S1 File — (ZIP) [file pone.0302213.s001.zip › FIGURA6/HL-HA.tif]

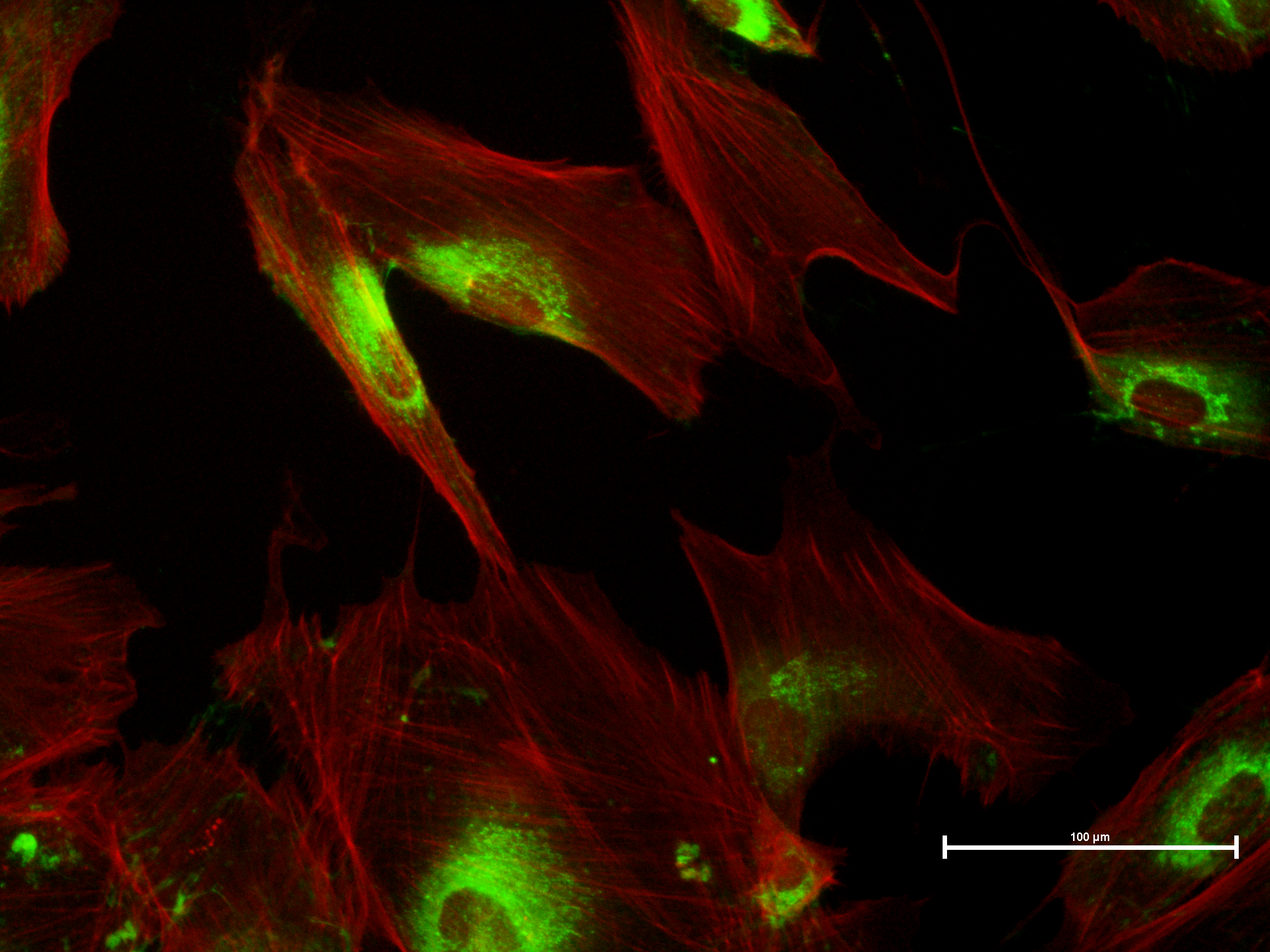

Supplement: S1 File — (ZIP) [file pone.0302213.s001.zip › FIGURA6/LHA.tif]

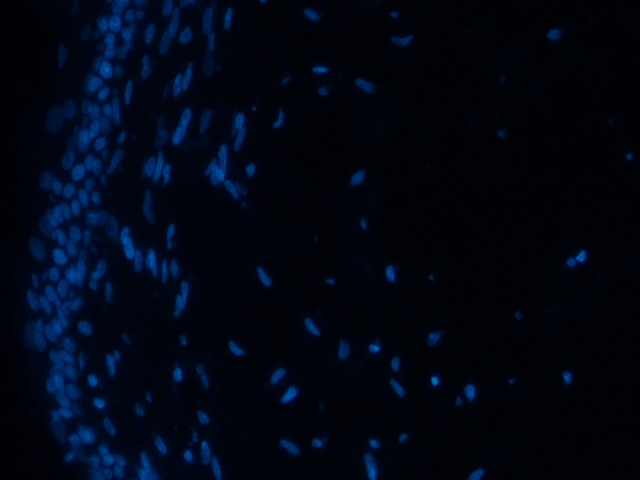

Supplement: S2 File — (ZIP) [file pone.0302213.s002.zip › FIGURA11/CTR/dapi_20x.tif]

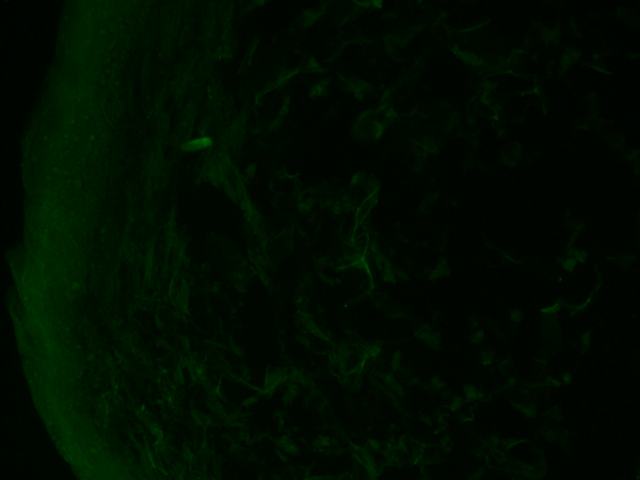

Supplement: S2 File — (ZIP) [file pone.0302213.s002.zip › FIGURA11/CTR/fitc_20x.tif]

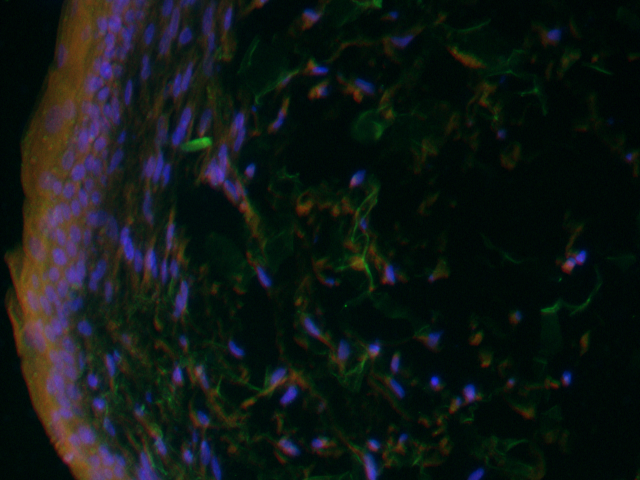

Supplement: S2 File — (ZIP) [file pone.0302213.s002.zip › FIGURA11/CTR/Miscelato_t.tif]

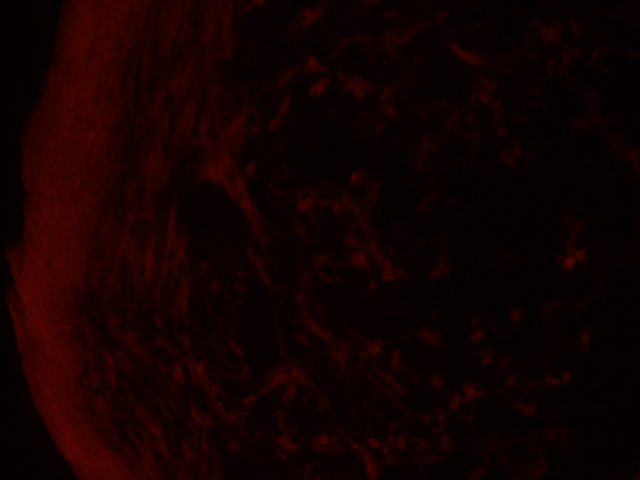

Supplement: S2 File — (ZIP) [file pone.0302213.s002.zip › FIGURA11/CTR/tritc_20x_2.tif]

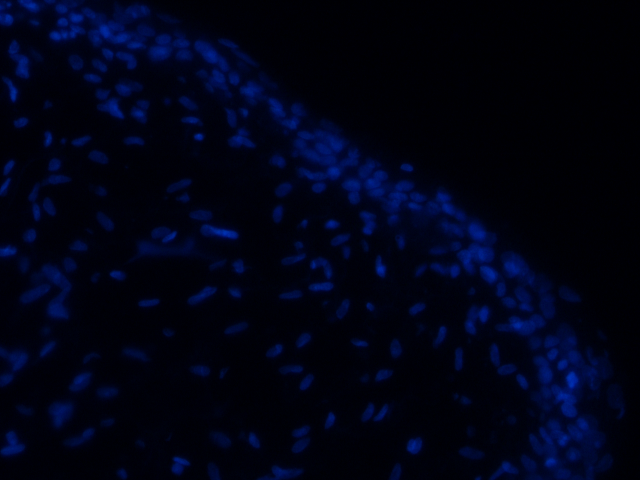

Supplement: S2 File — (ZIP) [file pone.0302213.s002.zip › FIGURA11/H L-HA/DAPI_TESS.tif]

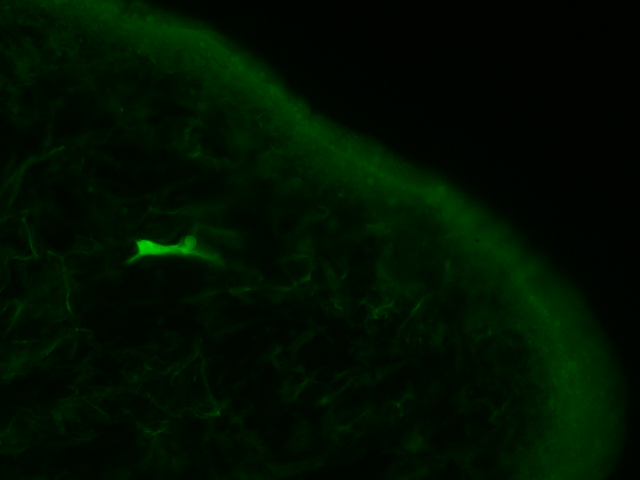

Supplement: S2 File — (ZIP) [file pone.0302213.s002.zip › FIGURA11/H L-HA/FITC_TESS.tif]

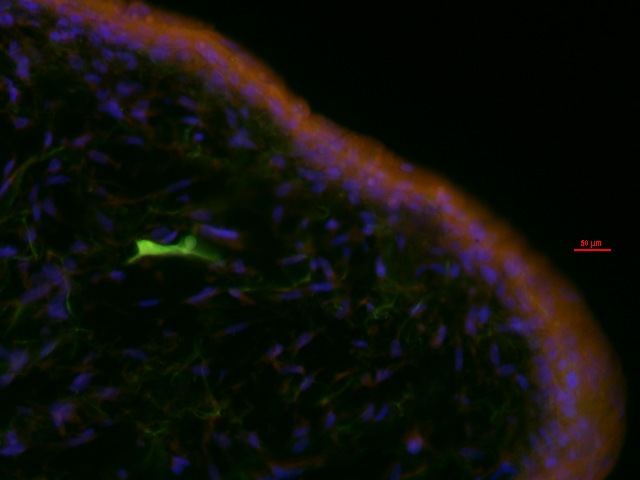

Supplement: S2 File — (ZIP) [file pone.0302213.s002.zip › FIGURA11/H L-HA/Miscelato_TESS.tif]

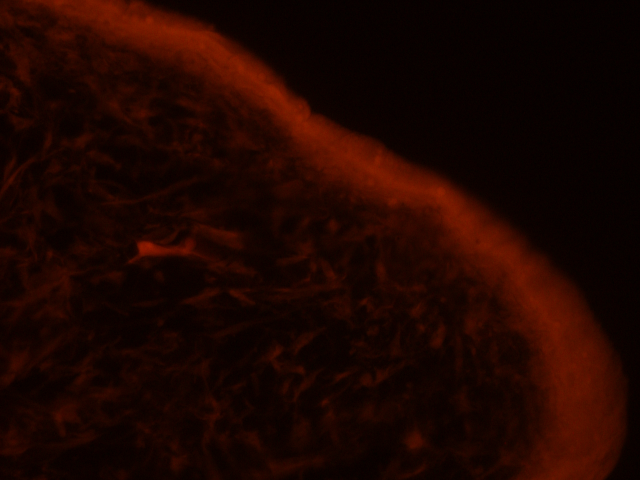

Supplement: S2 File — (ZIP) [file pone.0302213.s002.zip › FIGURA11/H L-HA/TESS_TRITC.tif]

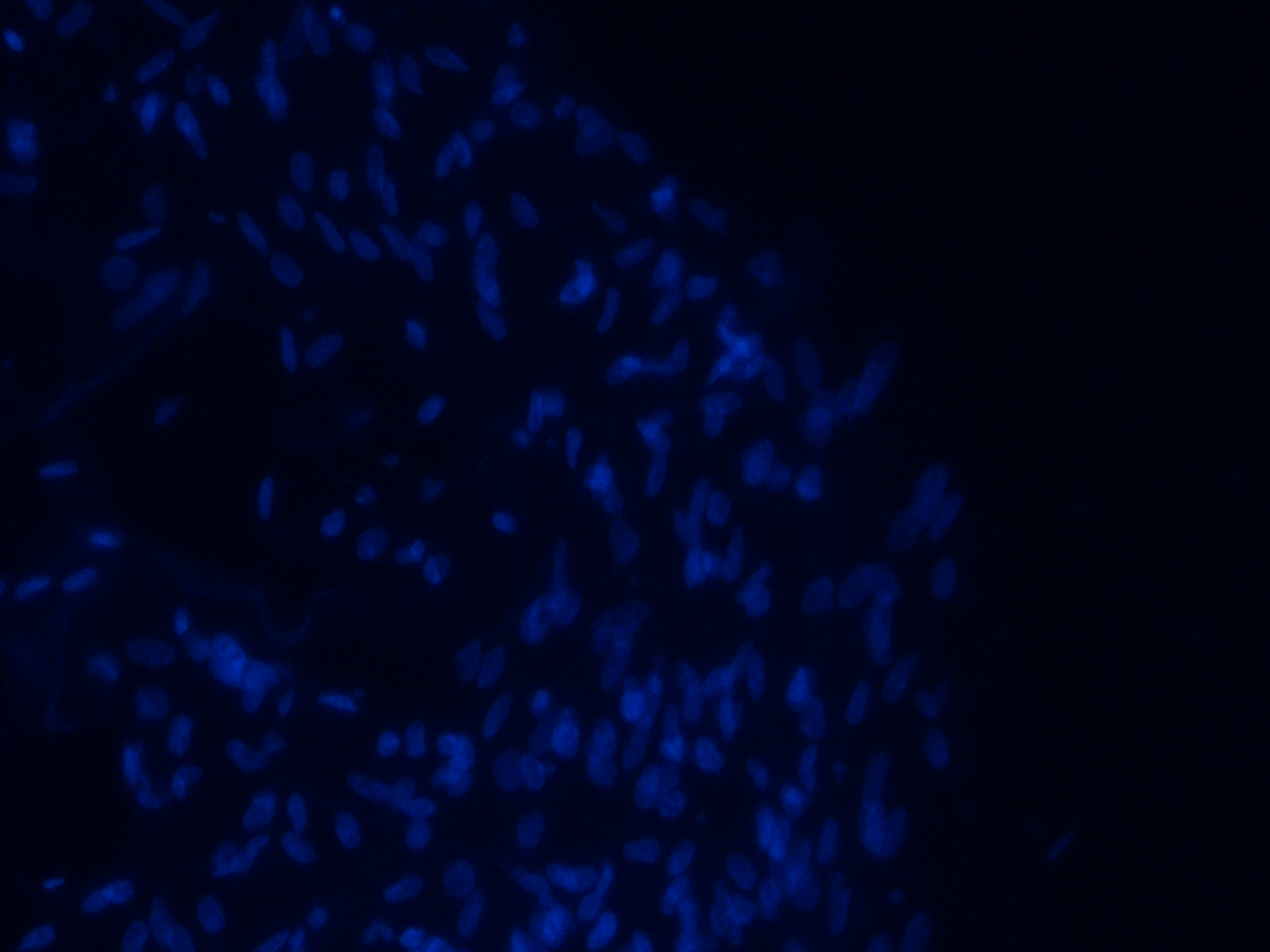

Supplement: S2 File — (ZIP) [file pone.0302213.s002.zip › FIGURA11/HHA/dapi_t_20x.tif]

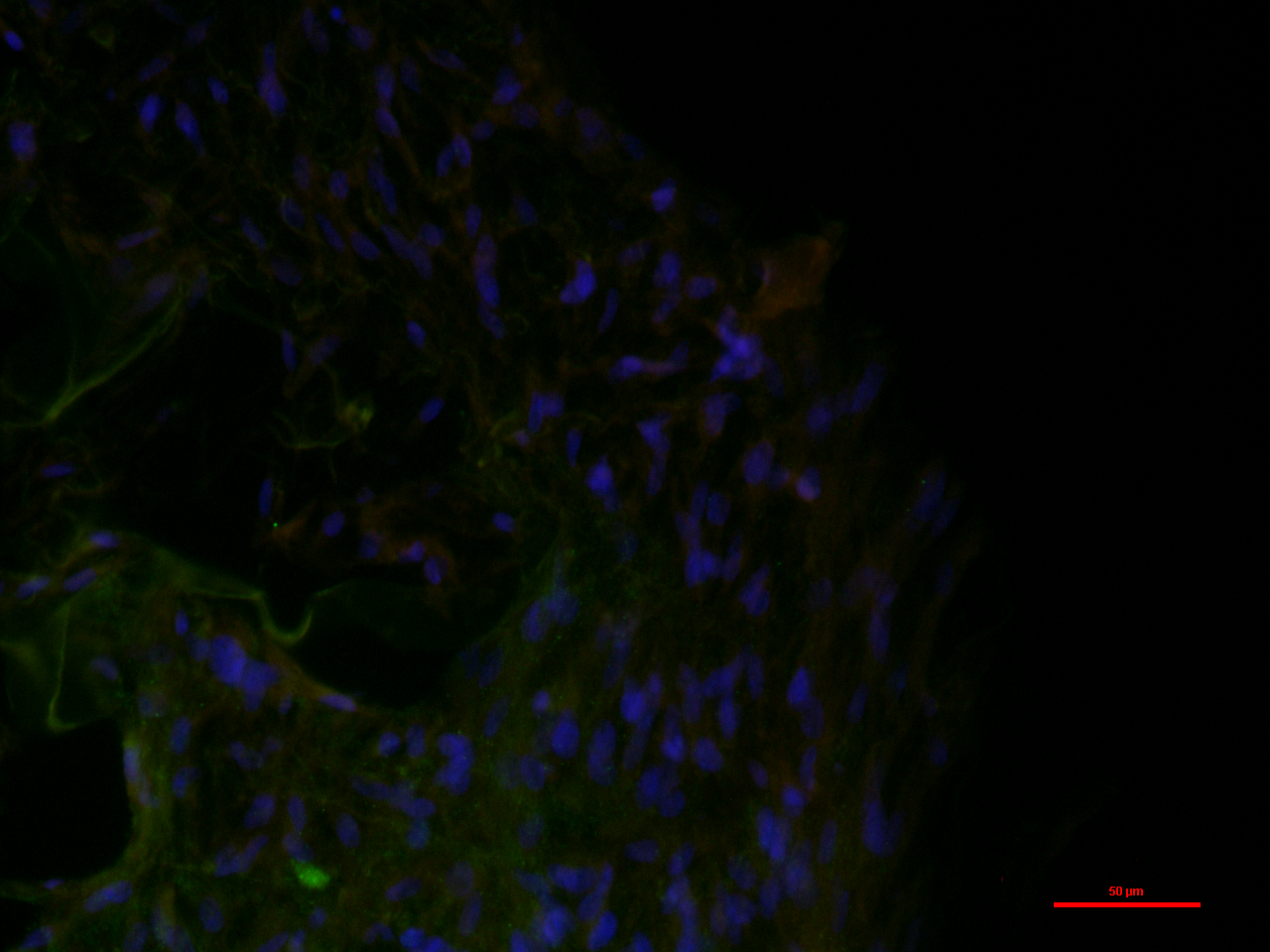

Supplement: S2 File — (ZIP) [file pone.0302213.s002.zip › FIGURA11/HHA/Miscelato 1.tif]

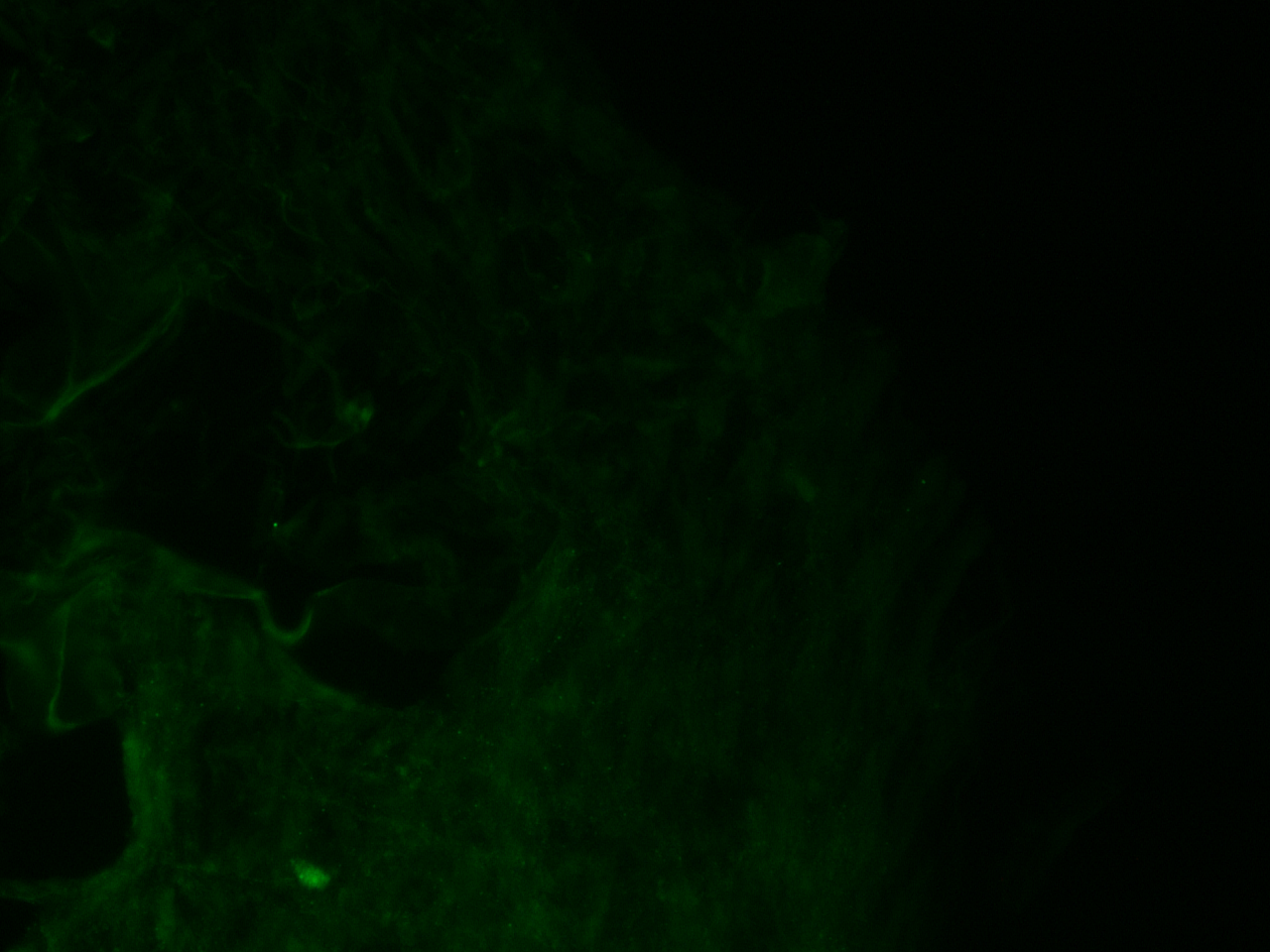

Supplement: S2 File — (ZIP) [file pone.0302213.s002.zip › FIGURA11/HHA/tess_20x.tif]

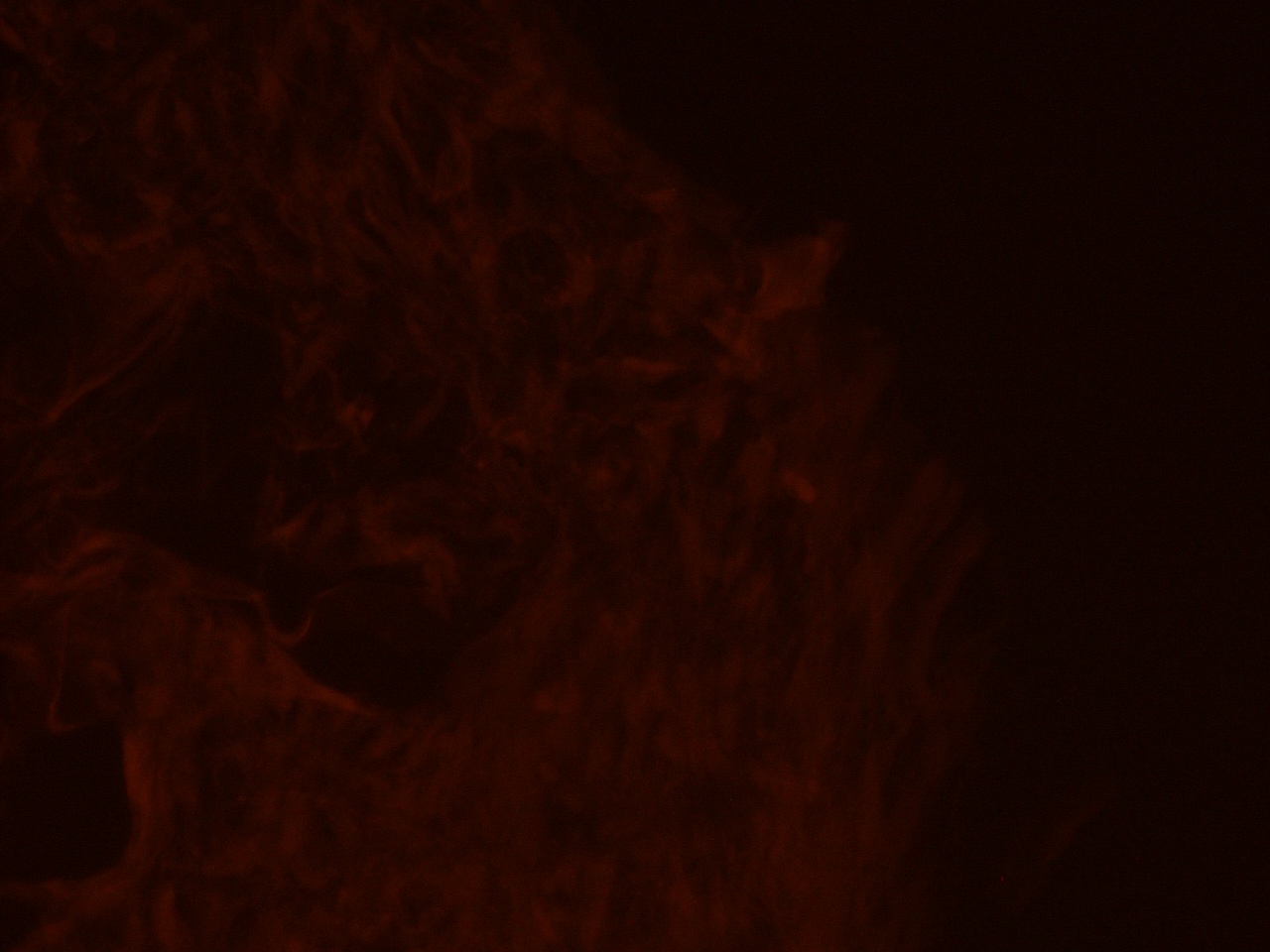

Supplement: S2 File — (ZIP) [file pone.0302213.s002.zip › FIGURA11/HHA/tessuto_20x.tif]

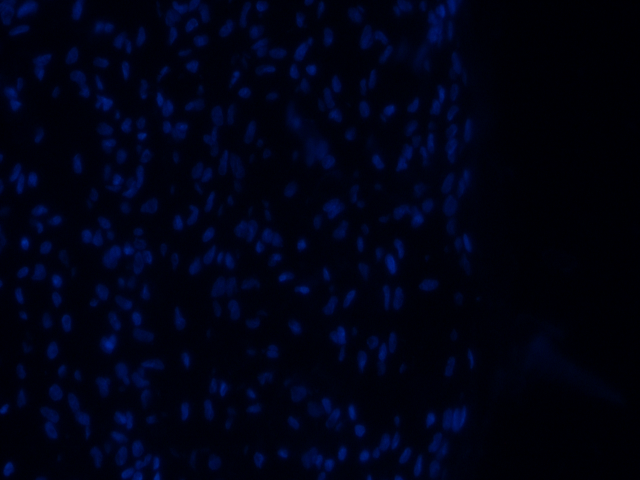

Supplement: S2 File — (ZIP) [file pone.0302213.s002.zip › FIGURA11/LHA/DAPI_TESS_1.tif]

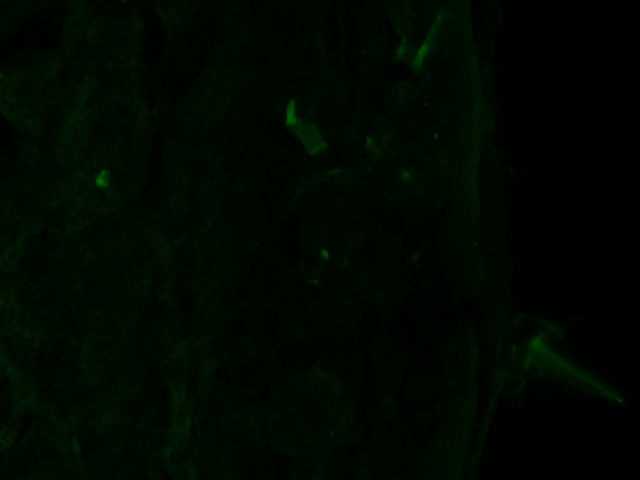

Supplement: S2 File — (ZIP) [file pone.0302213.s002.zip › FIGURA11/LHA/FITC_TESS_1.tif]

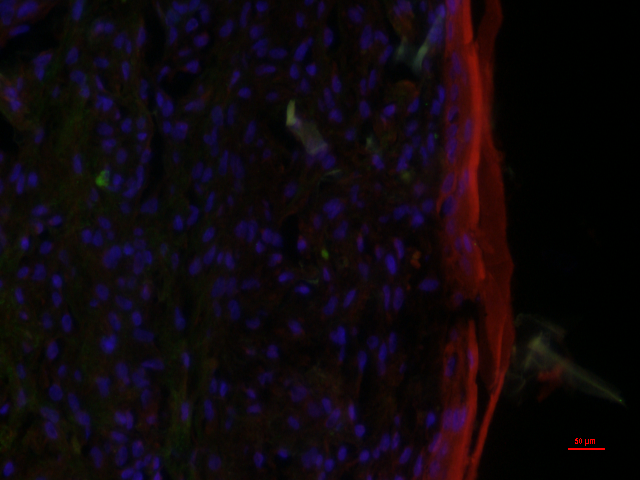

Supplement: S2 File — (ZIP) [file pone.0302213.s002.zip › FIGURA11/LHA/Miscelato_TESS_1.tif]

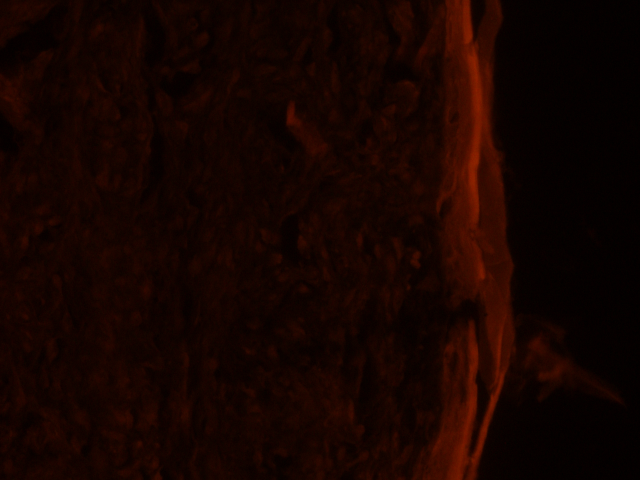

Supplement: S2 File — (ZIP) [file pone.0302213.s002.zip › FIGURA11/LHA/TRITC_TESS_1.tif]

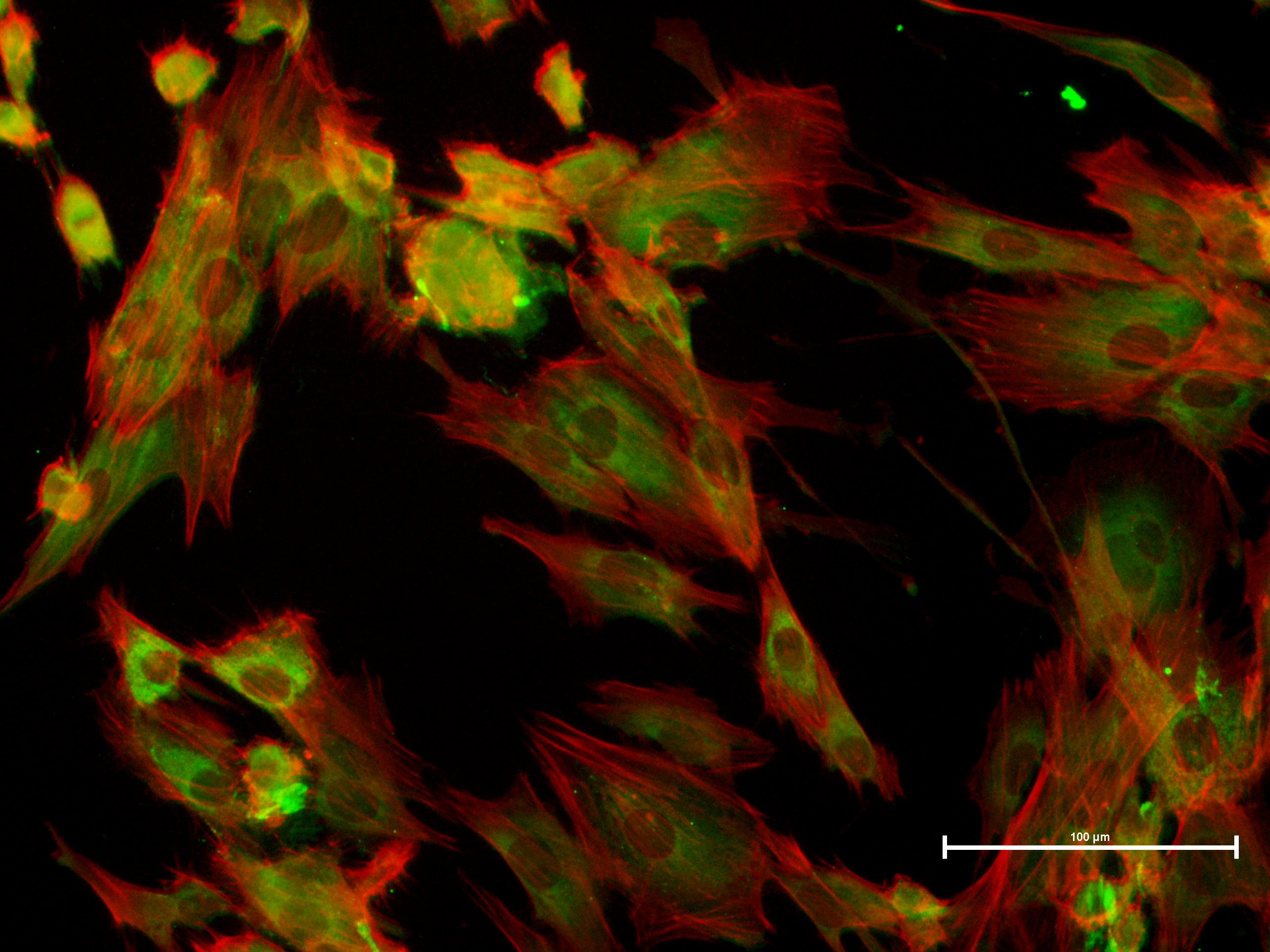

Supplement: S2 File — (ZIP) [file pone.0302213.s002.zip › FIGURA7/CTR.tif]

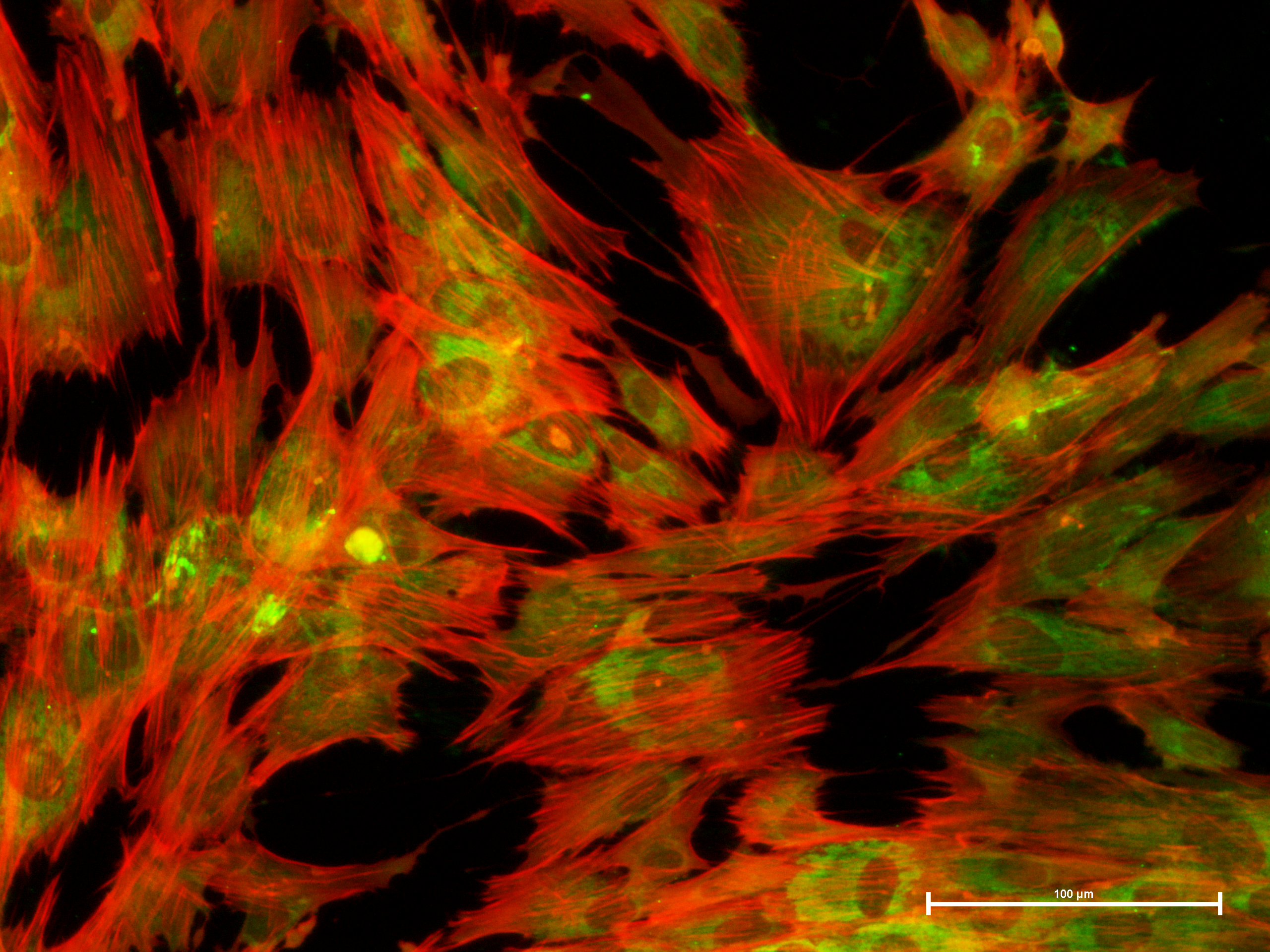

Supplement: S2 File — (ZIP) [file pone.0302213.s002.zip › FIGURA7/H L-HA.tif]

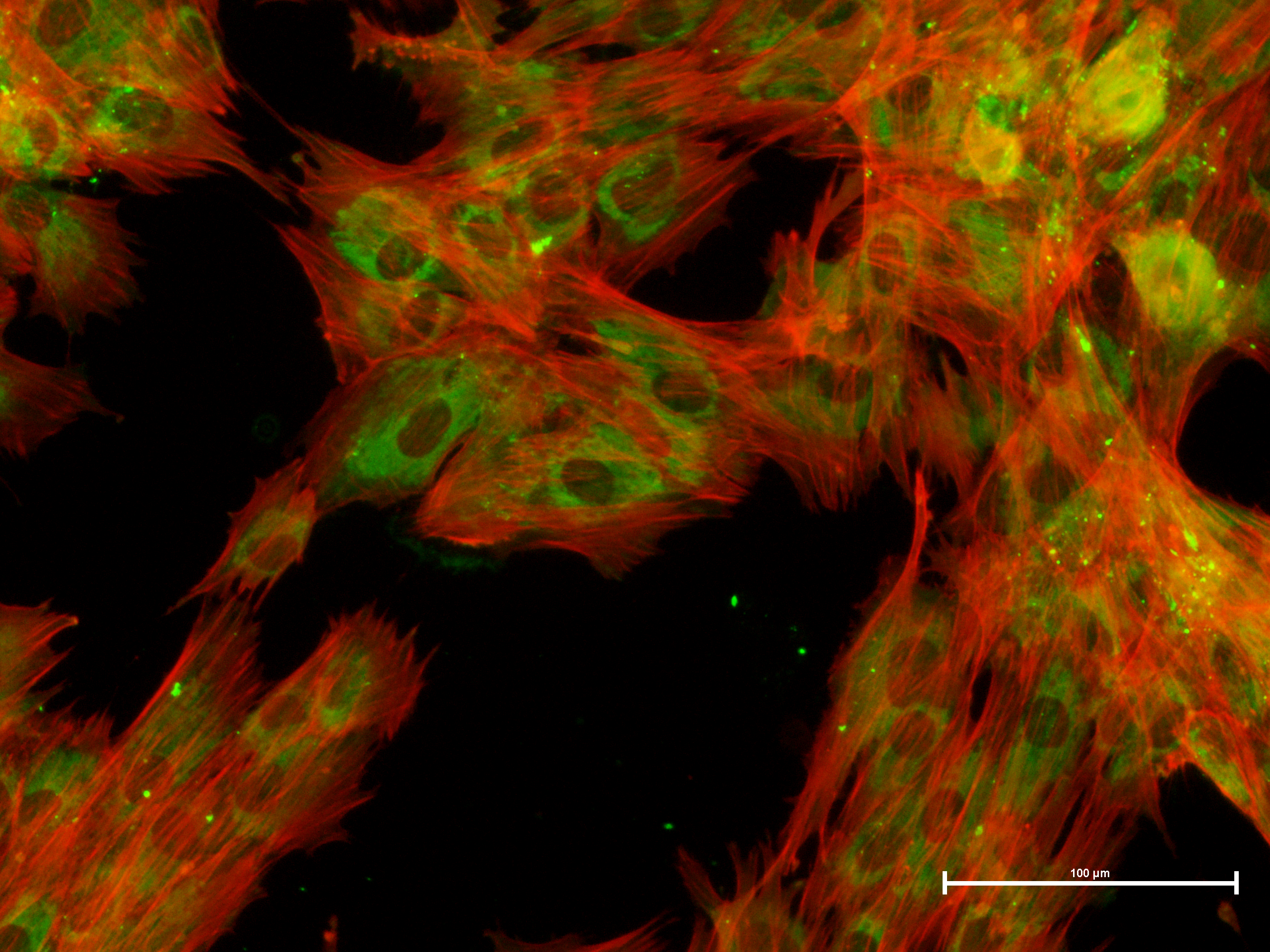

Supplement: S2 File — (ZIP) [file pone.0302213.s002.zip › FIGURA7/HHA.tif]

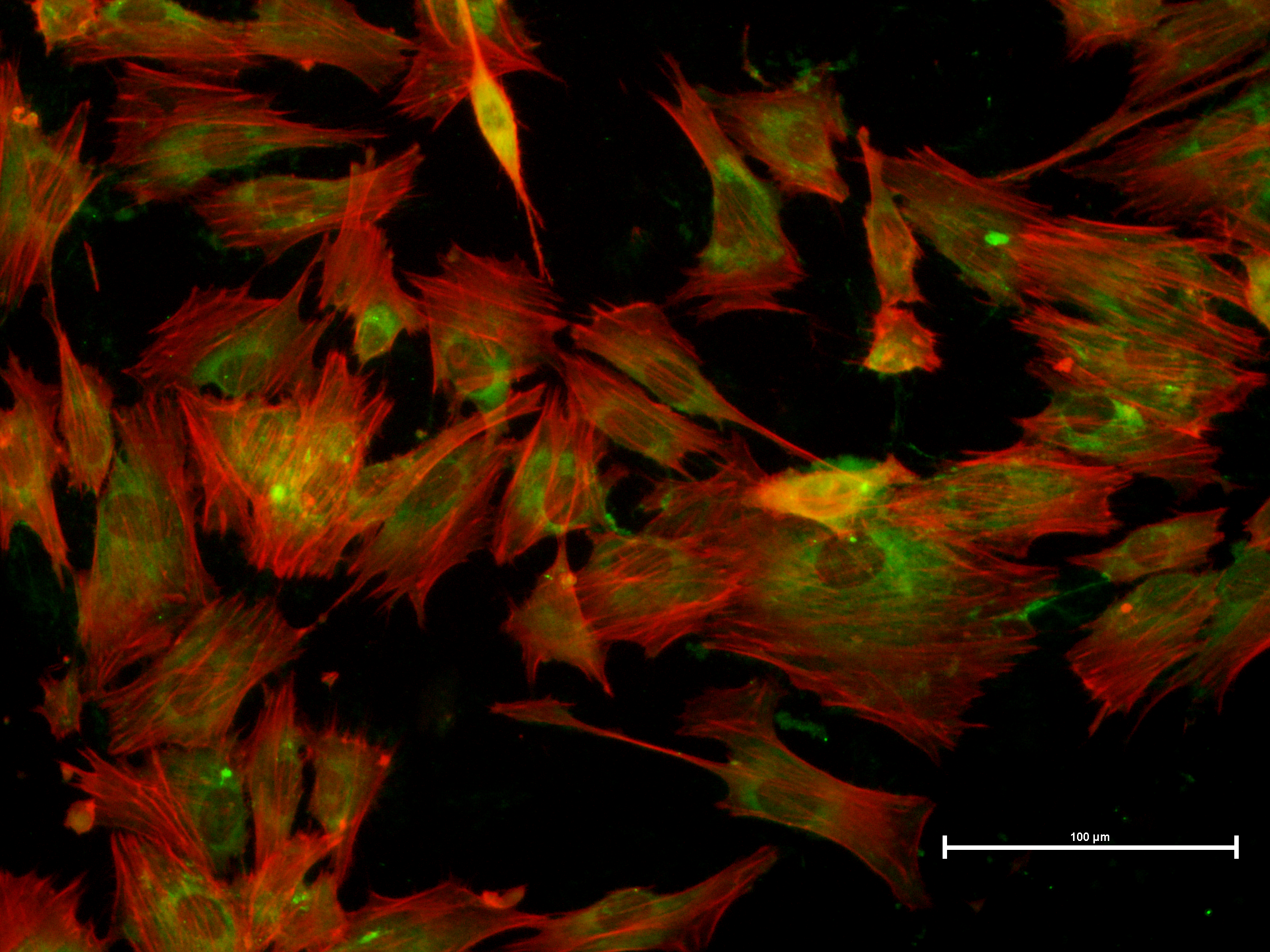

Supplement: S2 File — (ZIP) [file pone.0302213.s002.zip › FIGURA7/LHA.tif]

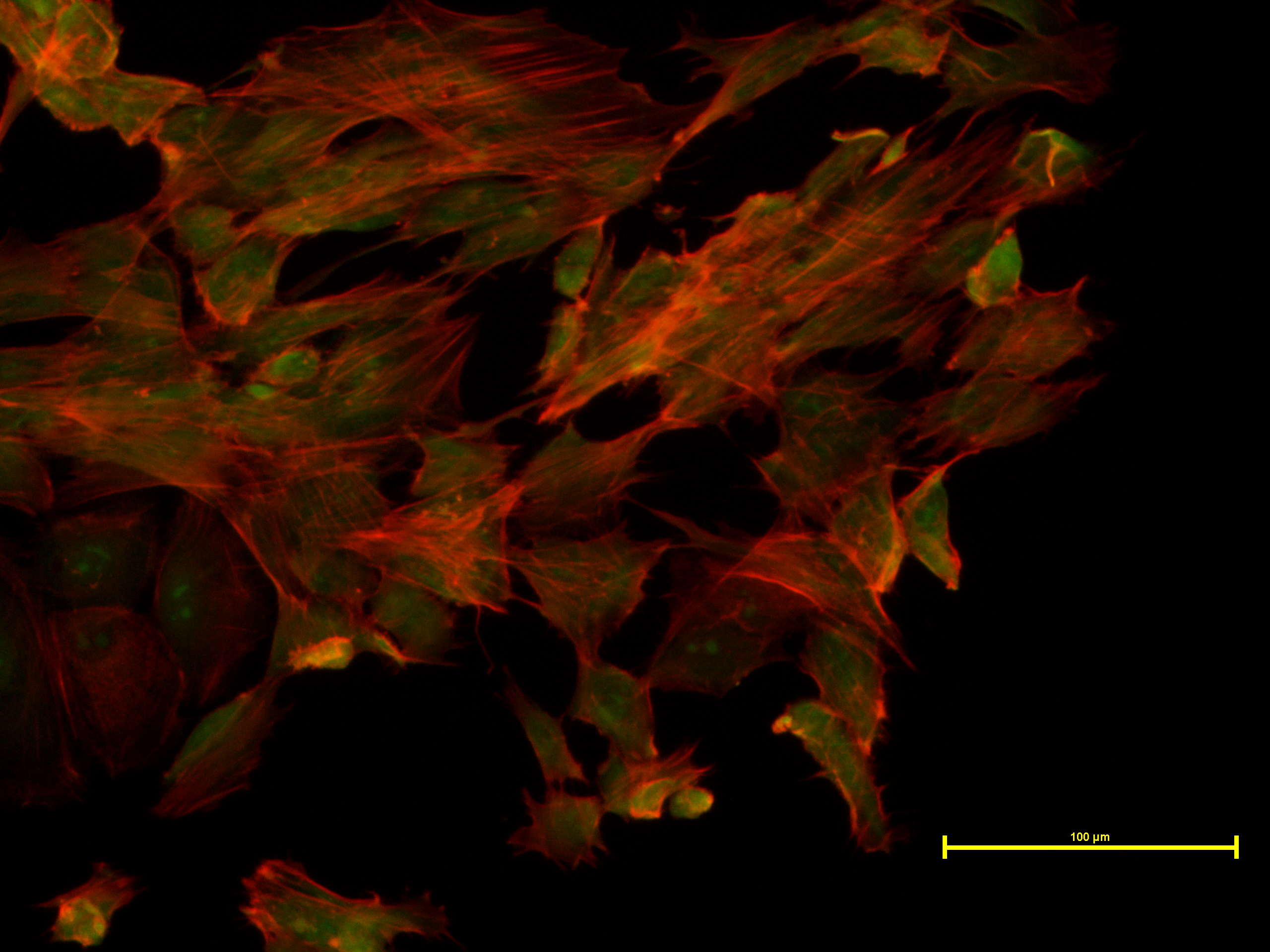

Supplement: S2 File — (ZIP) [file pone.0302213.s002.zip › FIGURA8/CTR.tif]

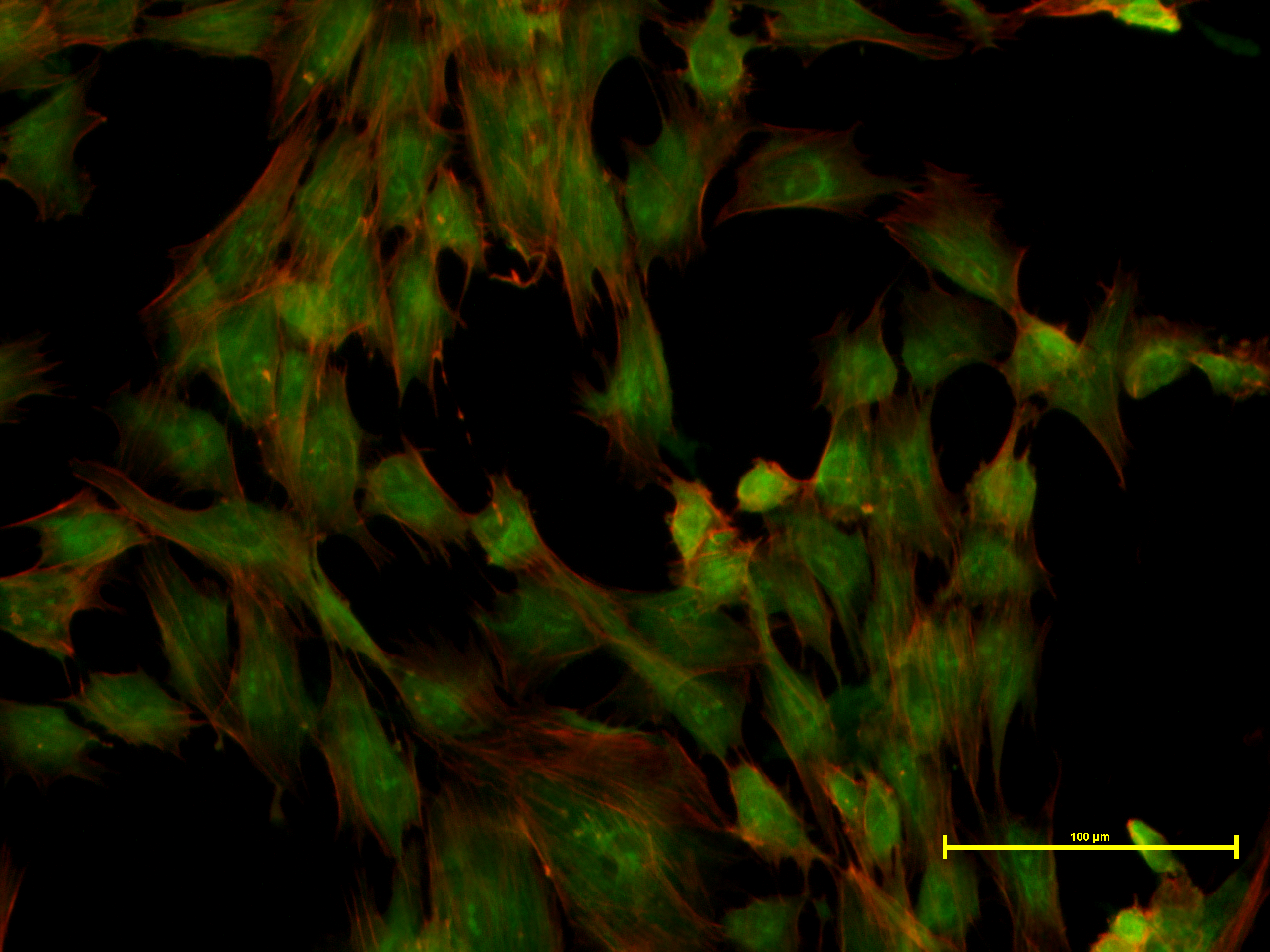

Supplement: S2 File — (ZIP) [file pone.0302213.s002.zip › FIGURA8/H L-HA.tif]

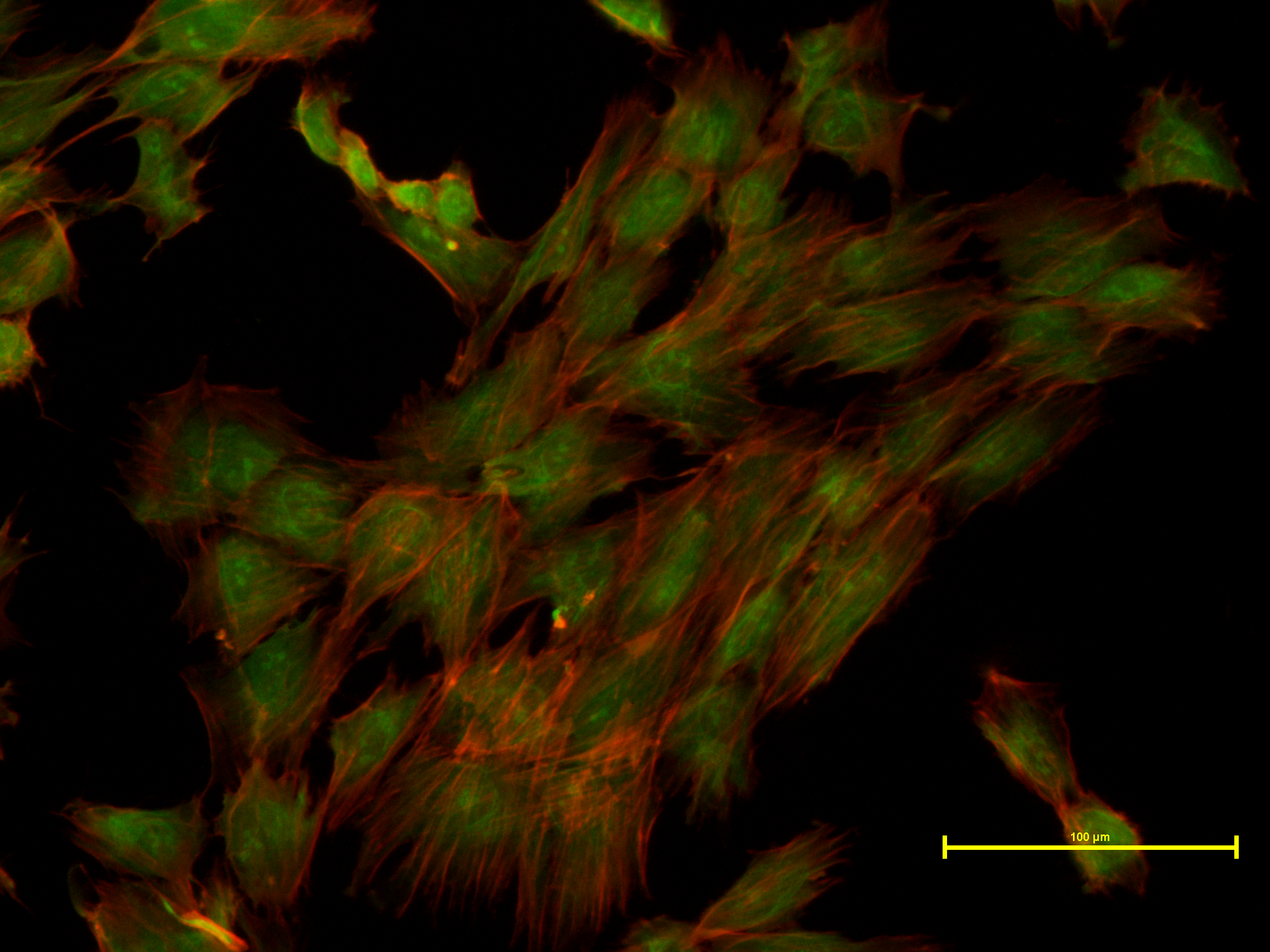

Supplement: S2 File — (ZIP) [file pone.0302213.s002.zip › FIGURA8/HHA.tif]

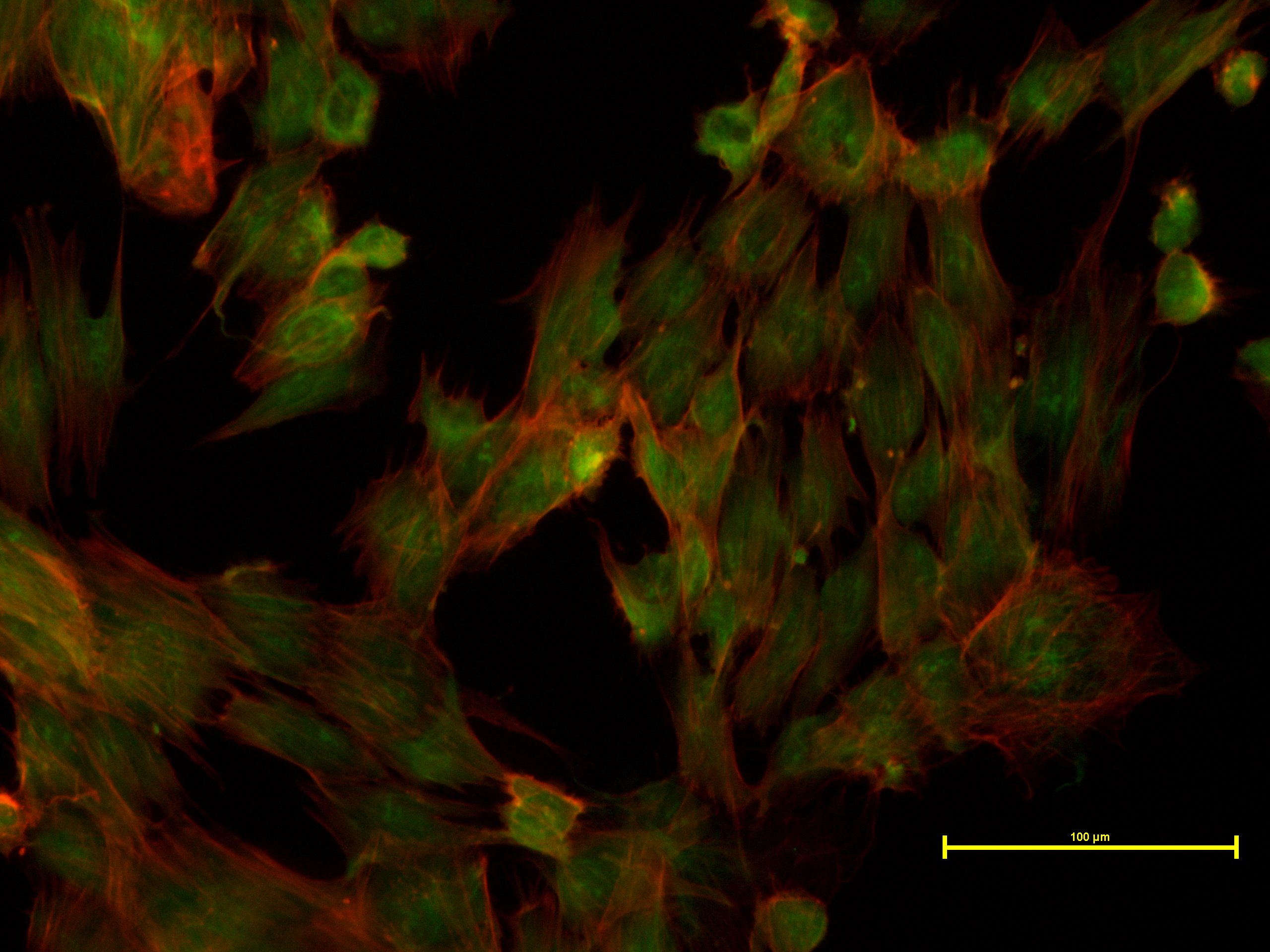

Supplement: S2 File — (ZIP) [file pone.0302213.s002.zip › FIGURA8/LHA.tif]

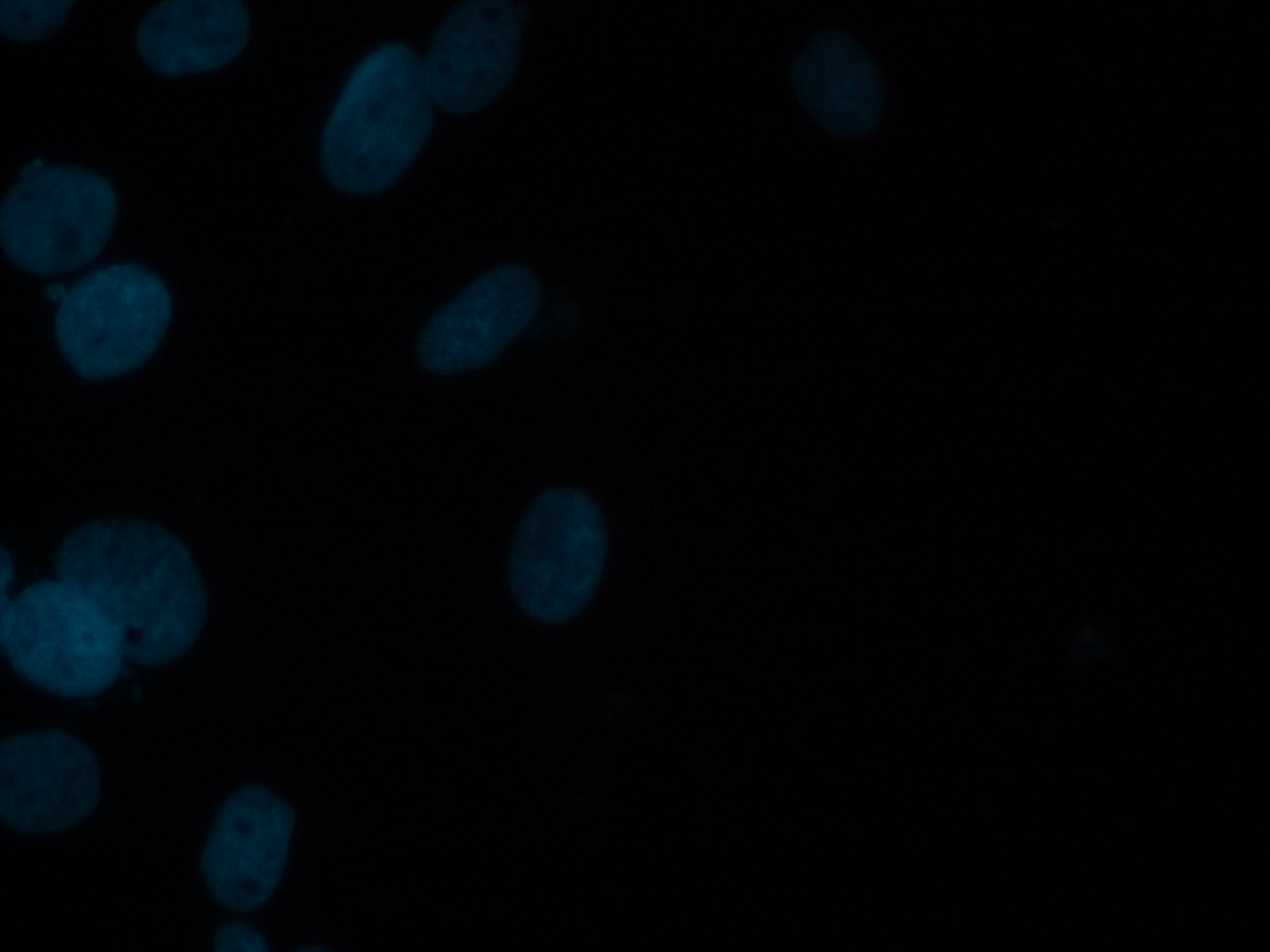

Supplement: S2 File — (ZIP) [file pone.0302213.s002.zip › FIGURA9/ctr/dapi_40x.tif]

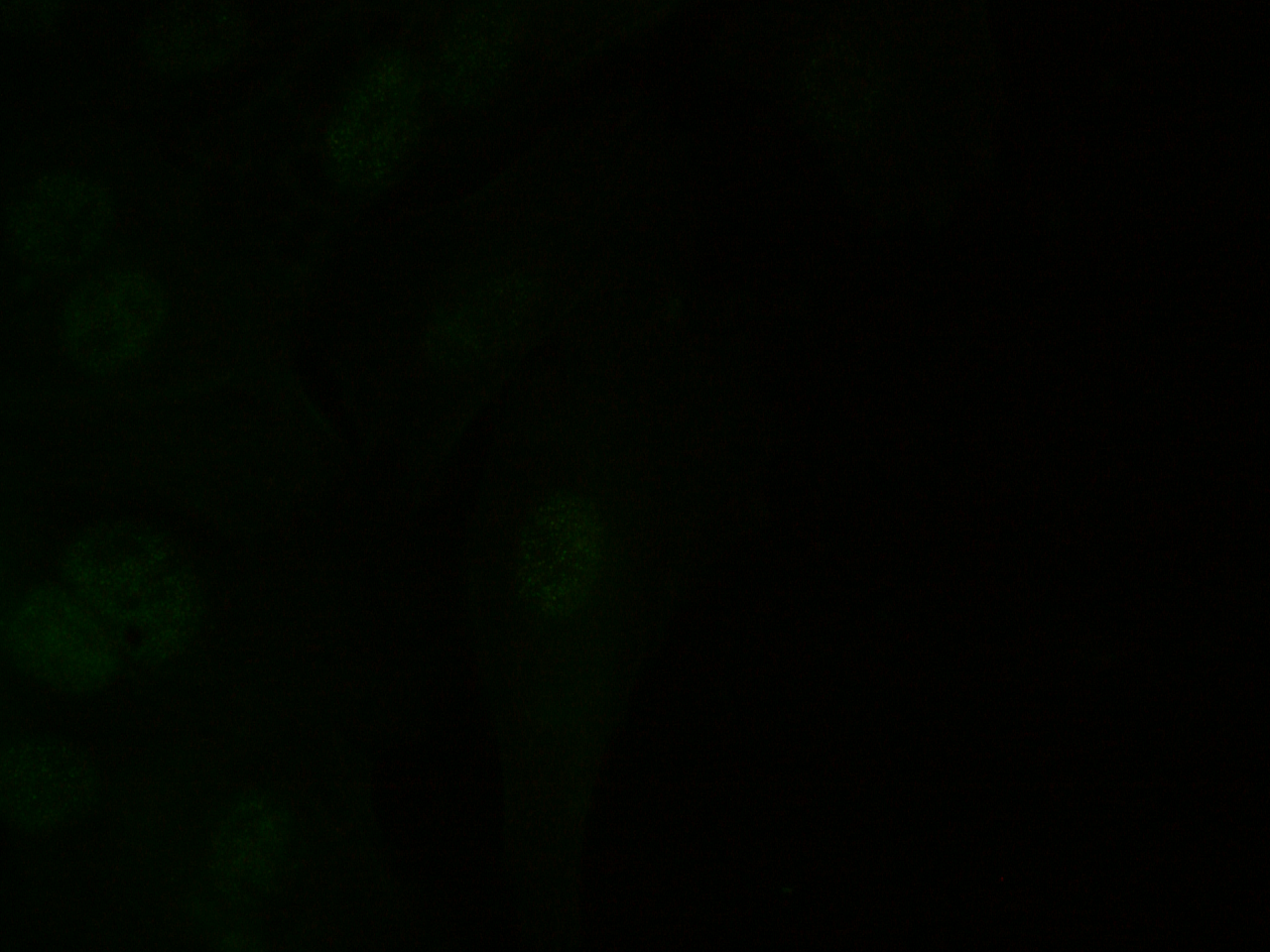

Supplement: S2 File — (ZIP) [file pone.0302213.s002.zip › FIGURA9/ctr/els_40x.tif]

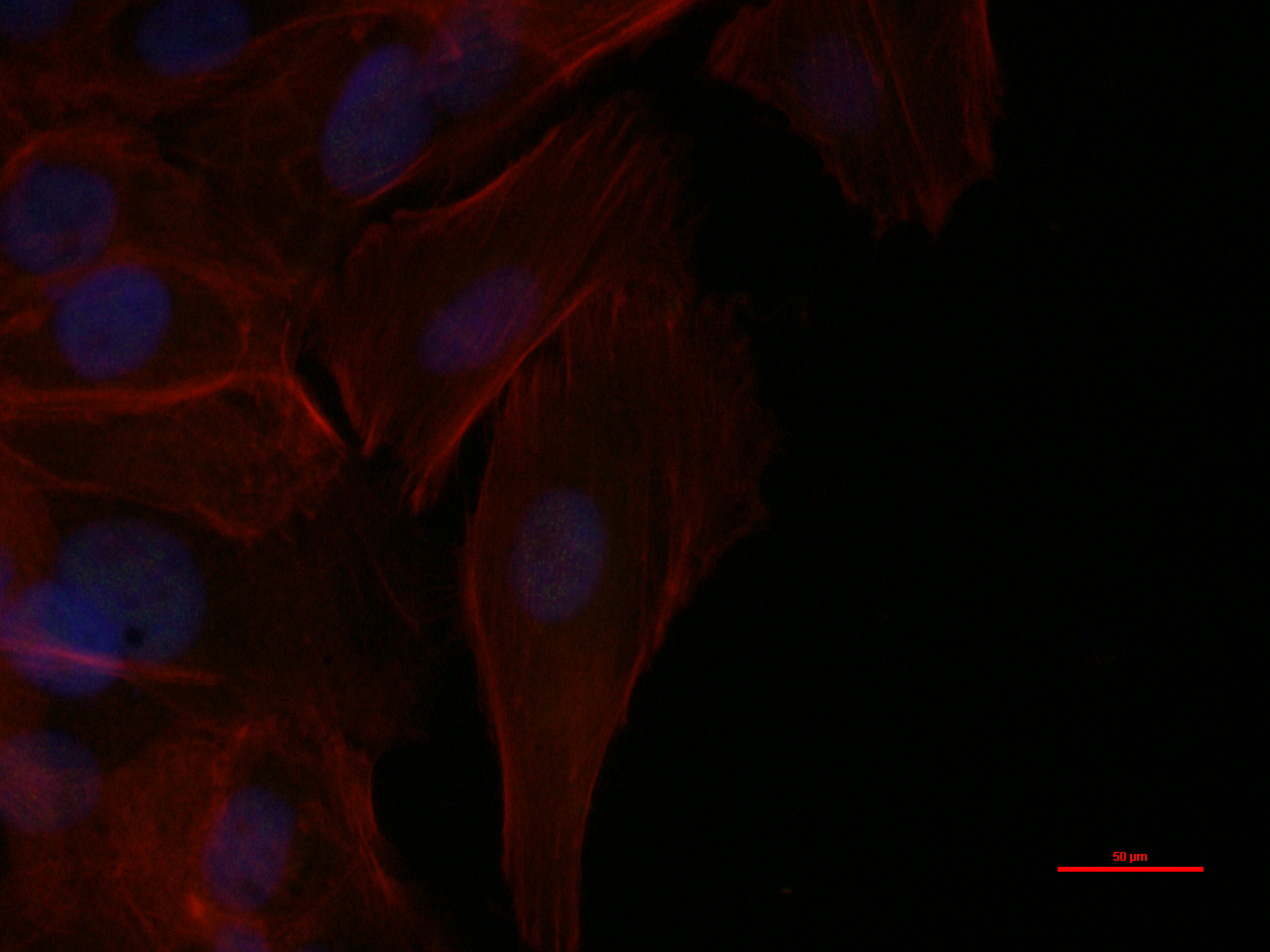

Supplement: S2 File — (ZIP) [file pone.0302213.s002.zip › FIGURA9/ctr/Miscelato_40x.tif]

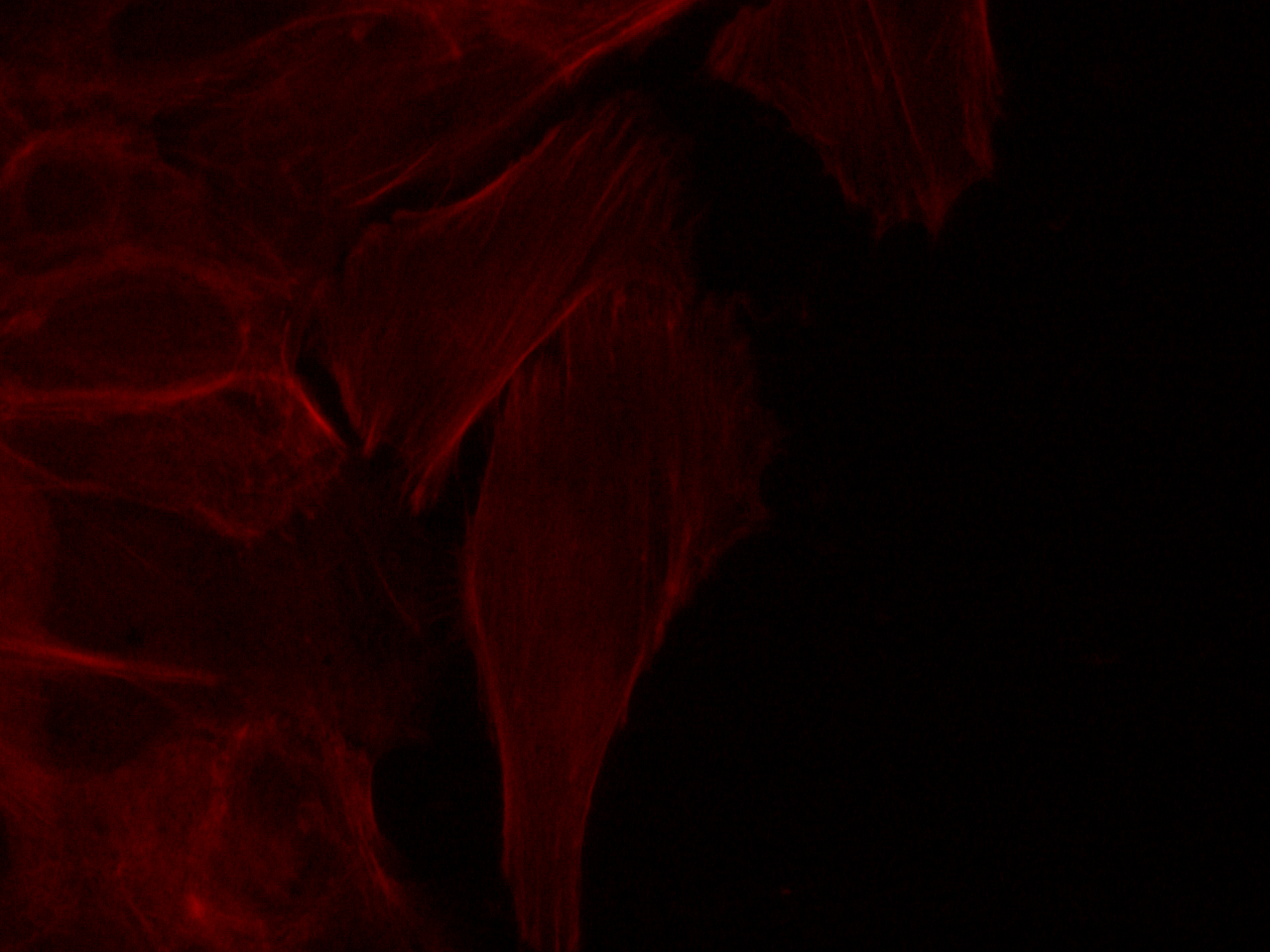

Supplement: S2 File — (ZIP) [file pone.0302213.s002.zip › FIGURA9/ctr/tritc_40x.tif]

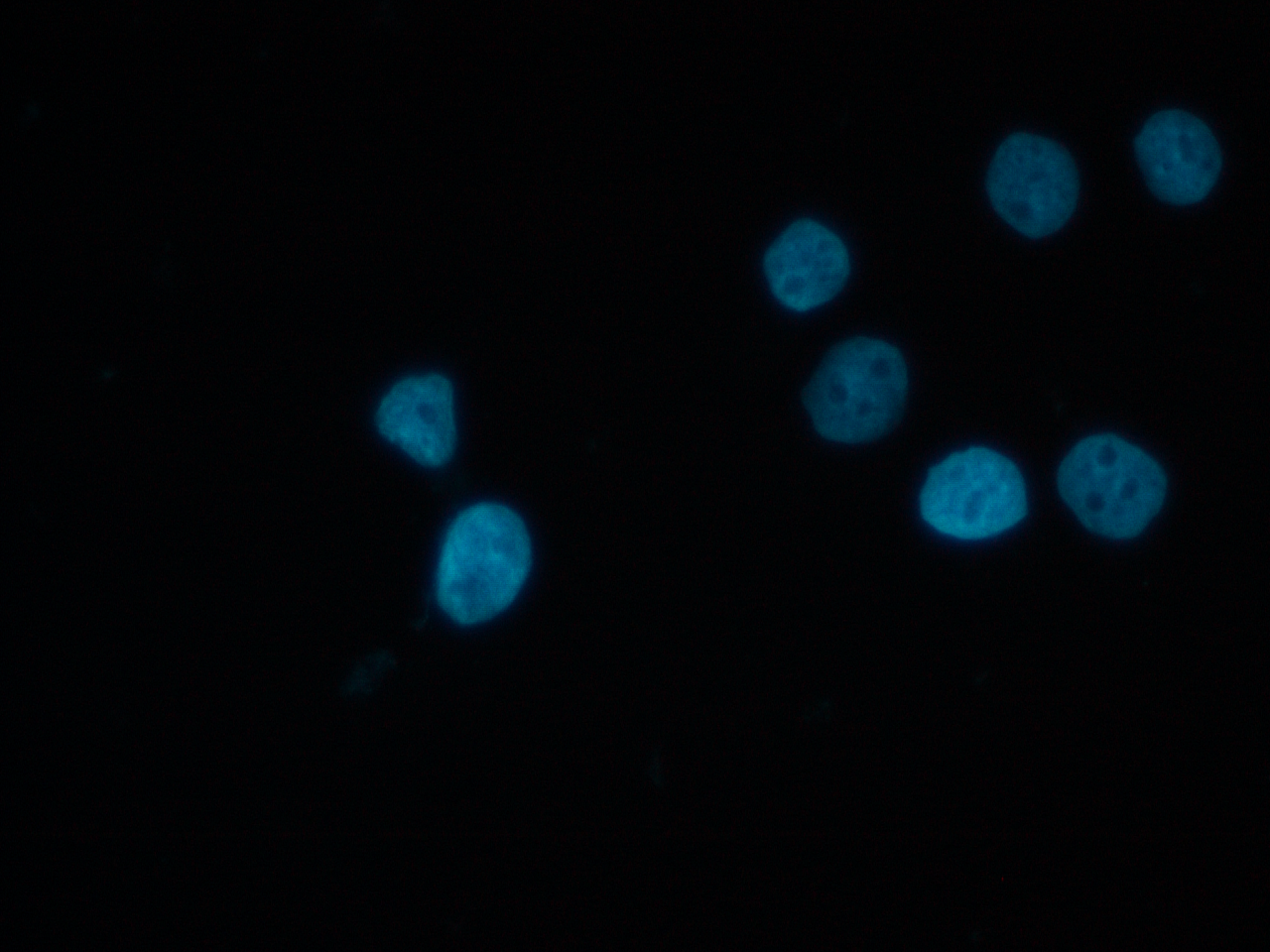

Supplement: S2 File — (ZIP) [file pone.0302213.s002.zip › FIGURA9/HHA/dapi.tif]

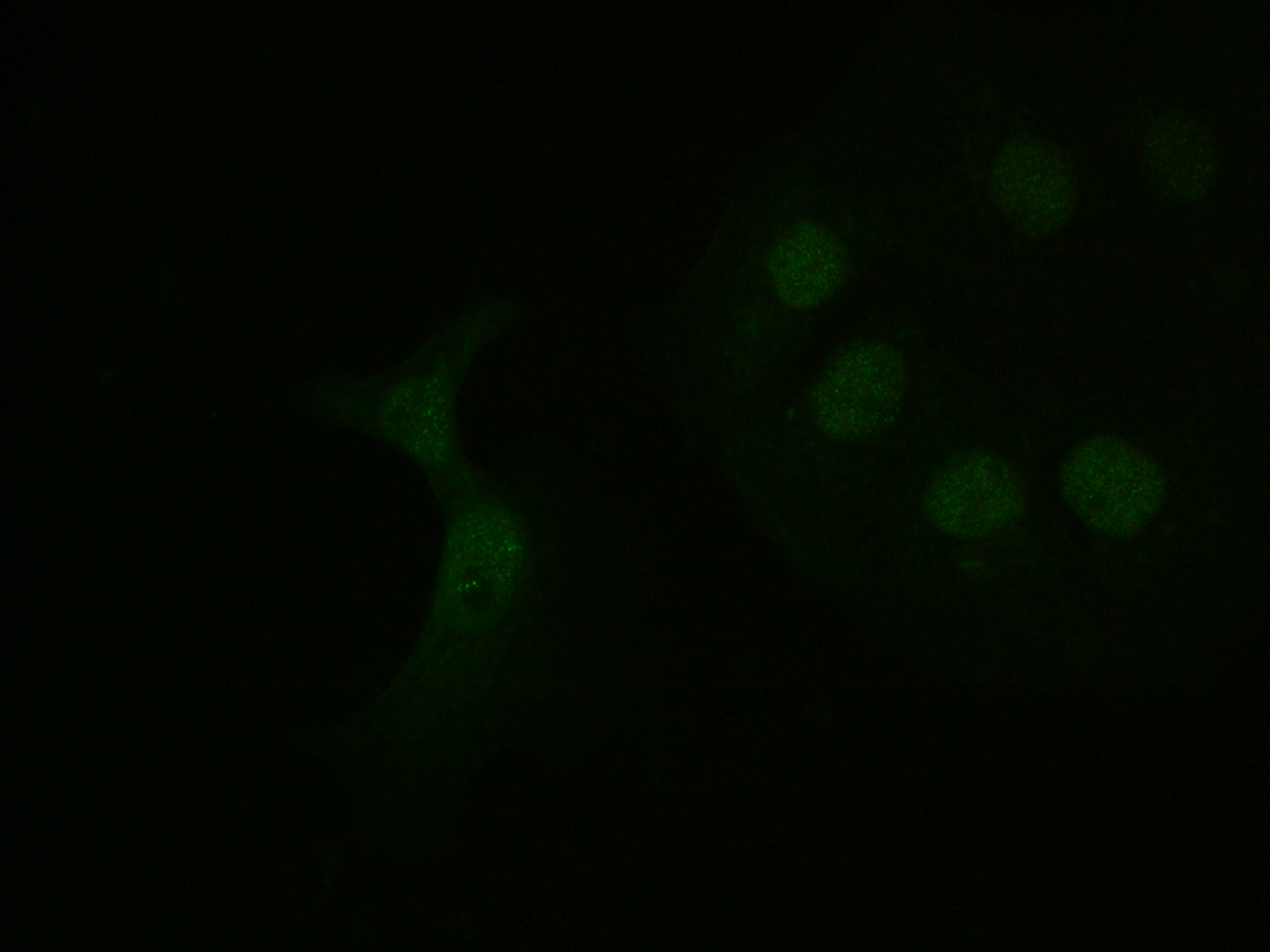

Supplement: S2 File — (ZIP) [file pone.0302213.s002.zip › FIGURA9/HHA/els.tif]

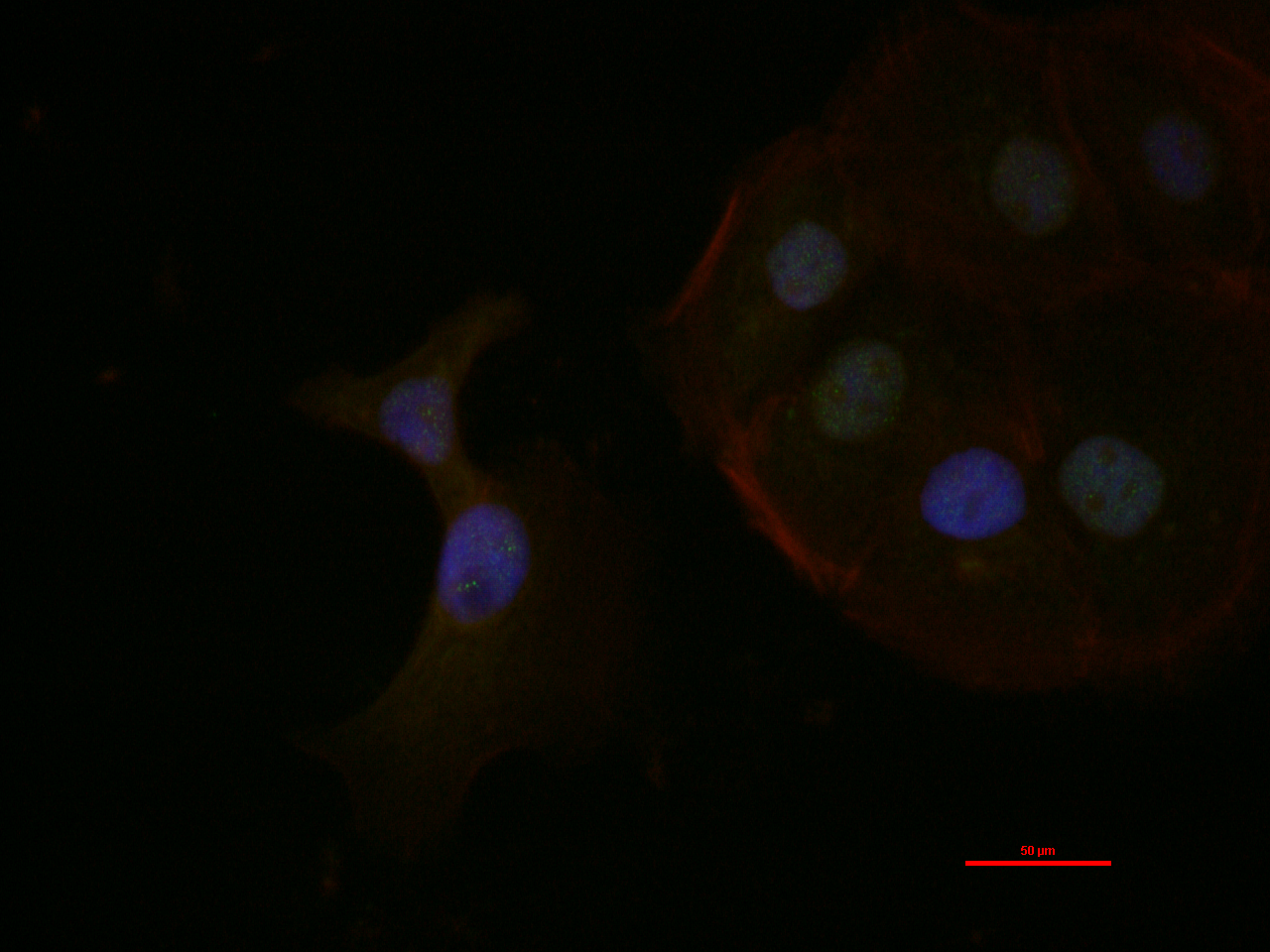

Supplement: S2 File — (ZIP) [file pone.0302213.s002.zip › FIGURA9/HHA/miscelato_40x.tif]

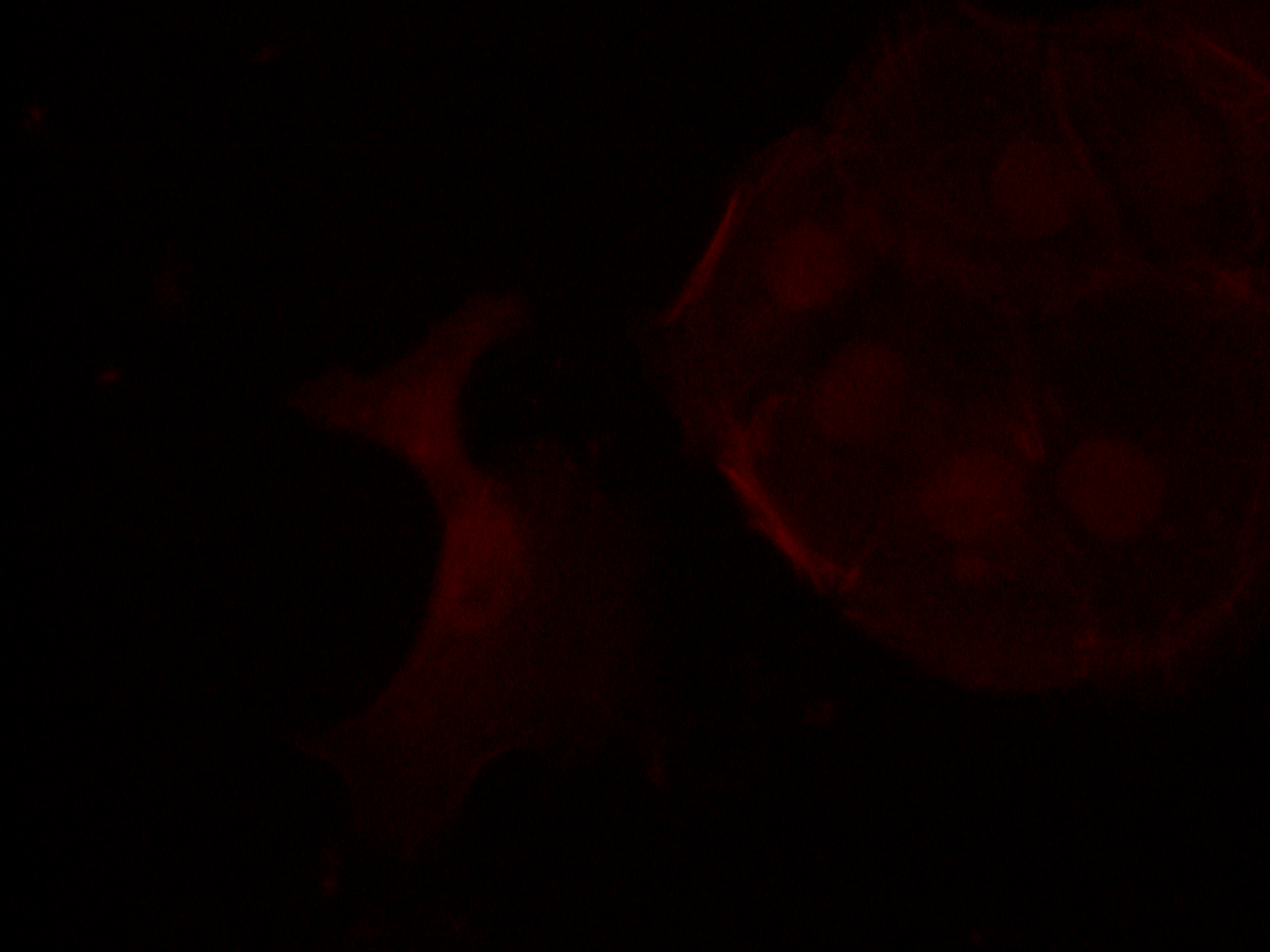

Supplement: S2 File — (ZIP) [file pone.0302213.s002.zip › FIGURA9/HHA/tritc.tif]

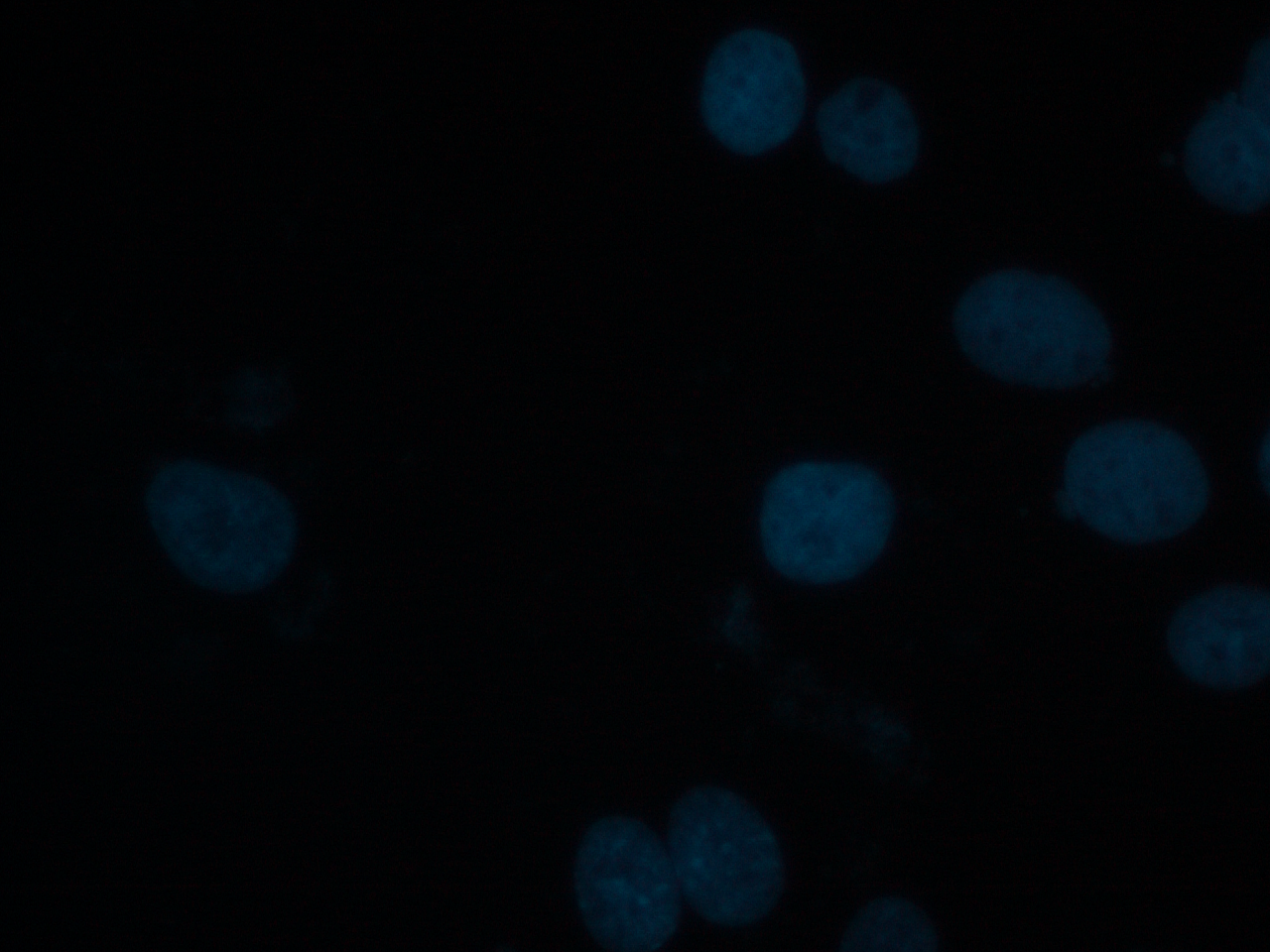

Supplement: S2 File — (ZIP) [file pone.0302213.s002.zip › FIGURA9/LHA/dapi_2_40x.tif]

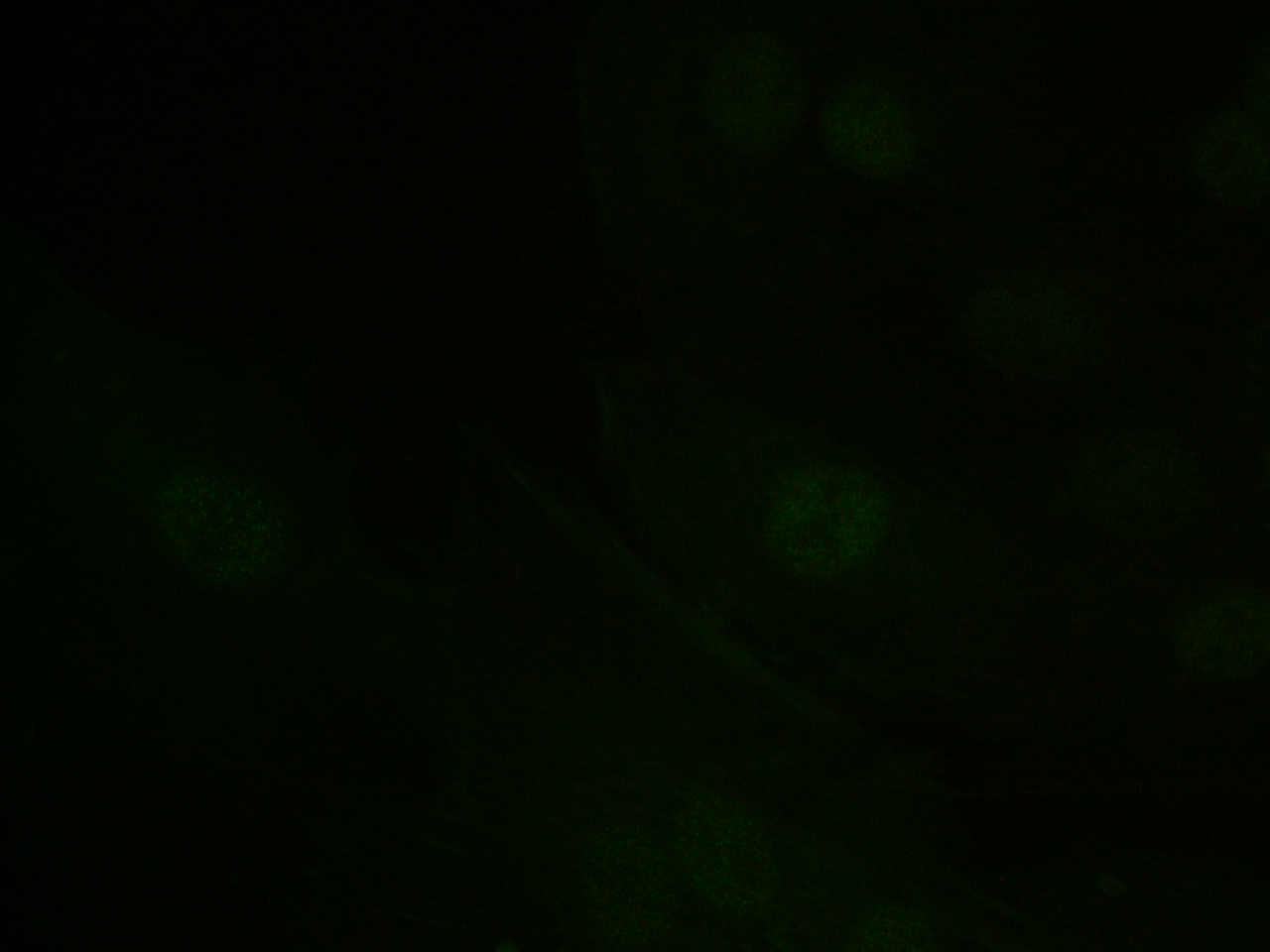

Supplement: S2 File — (ZIP) [file pone.0302213.s002.zip › FIGURA9/LHA/els_2_40x.tif]

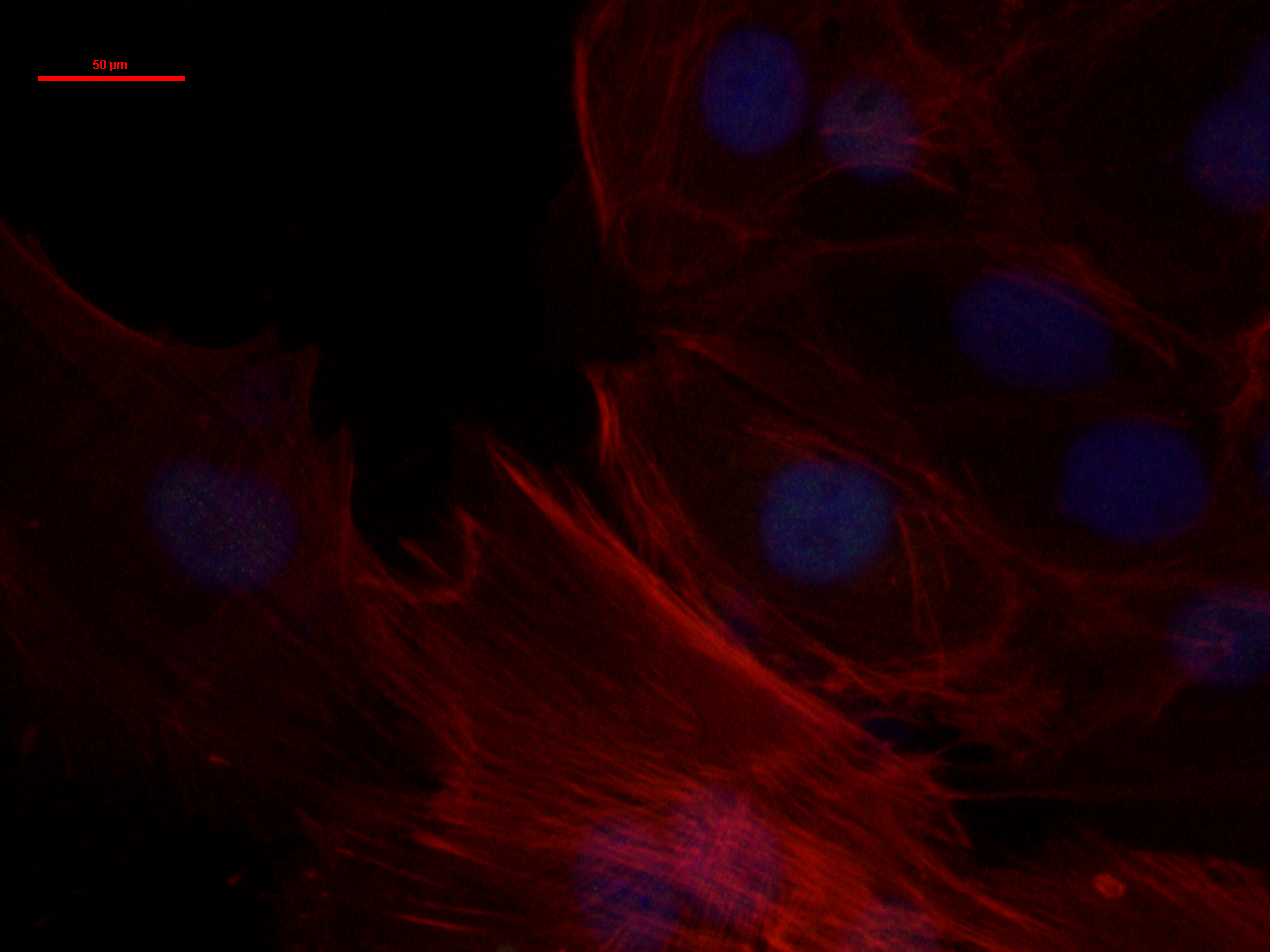

Supplement: S2 File — (ZIP) [file pone.0302213.s002.zip › FIGURA9/LHA/Miscelato_2_40x.tif]

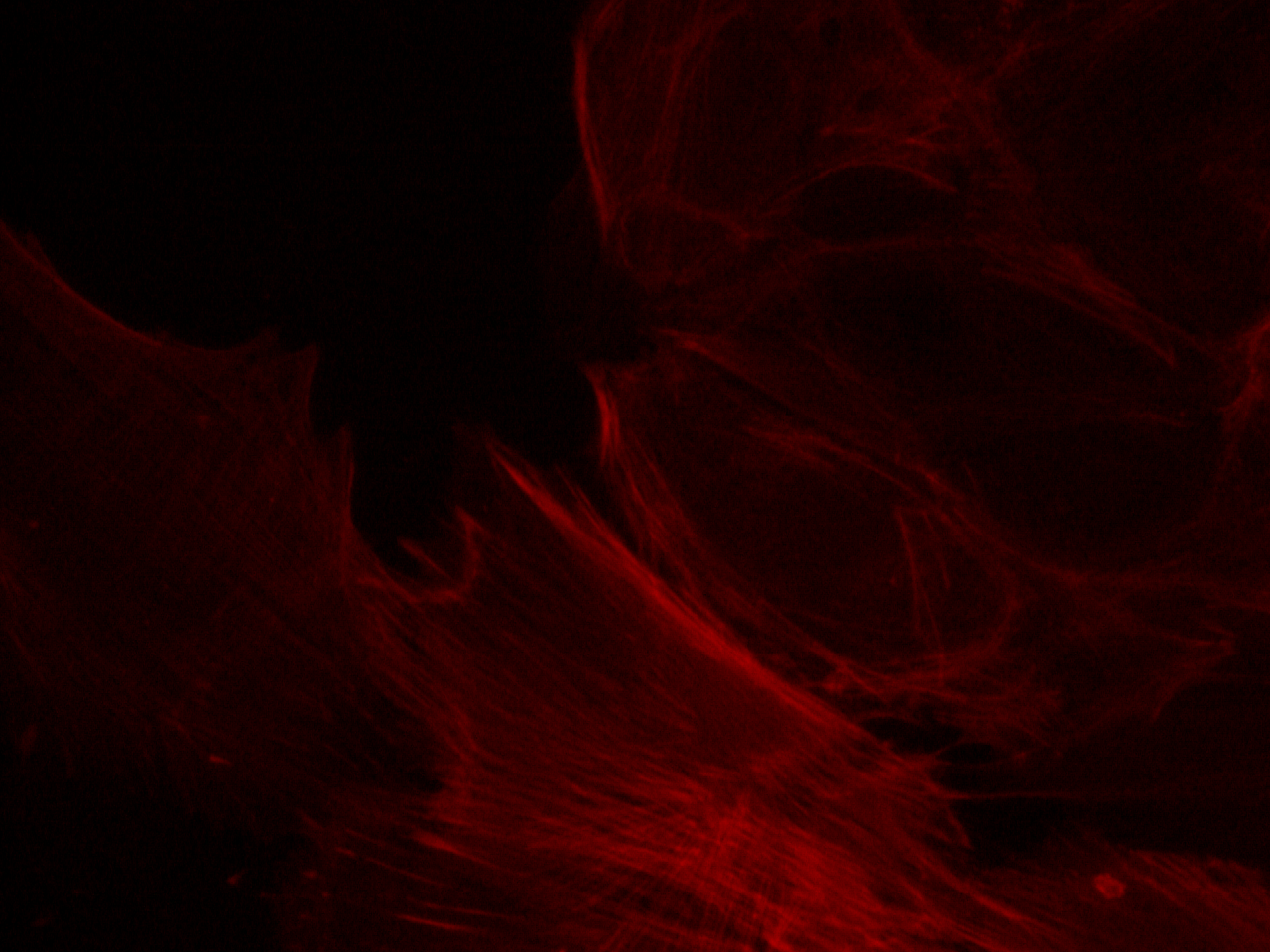

Supplement: S2 File — (ZIP) [file pone.0302213.s002.zip › FIGURA9/LHA/tritc_2_40x.tif]

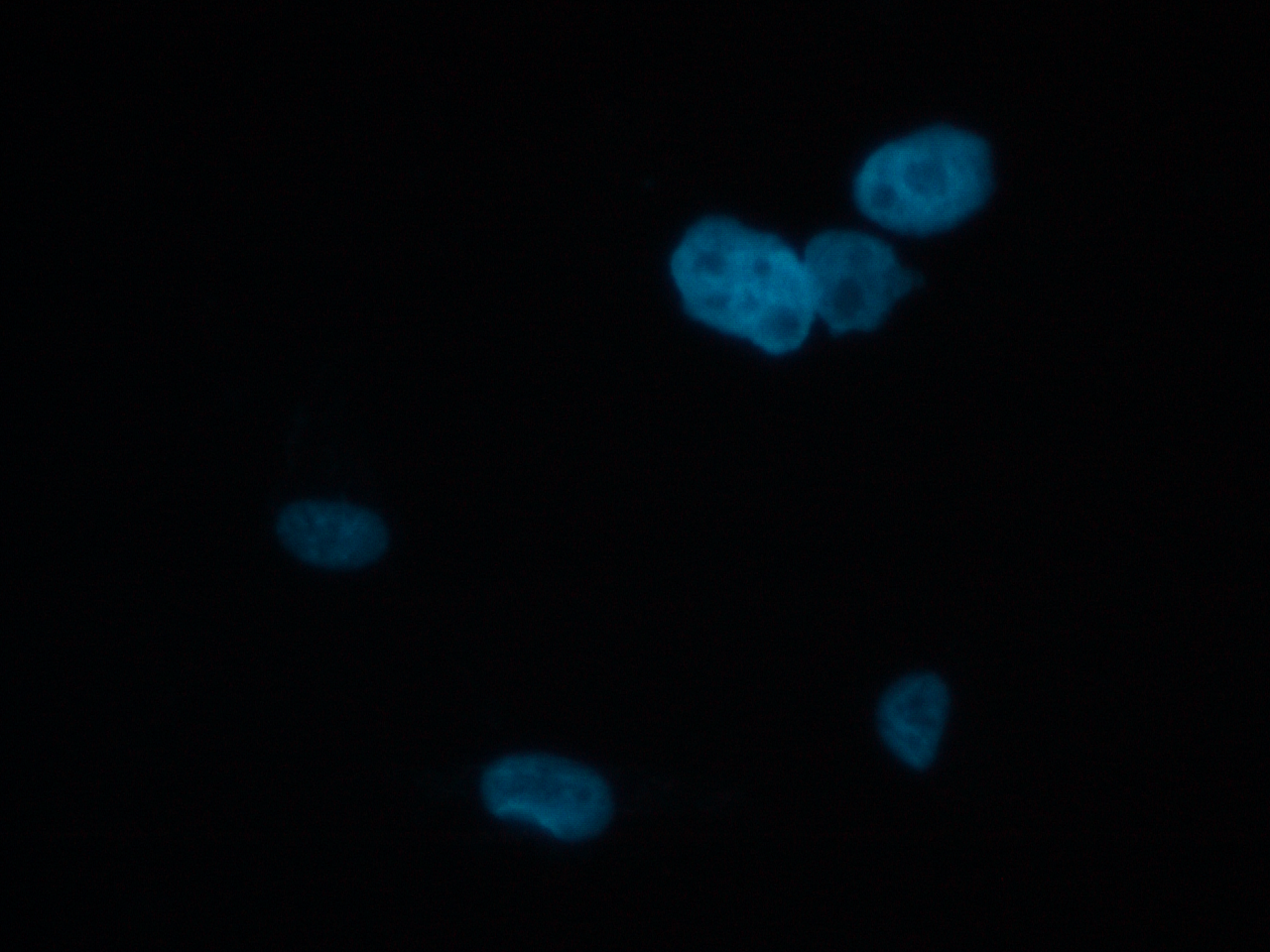

Supplement: S2 File — (ZIP) [file pone.0302213.s002.zip › FIGURA9/PROFHILO/dapi_40x.tif]

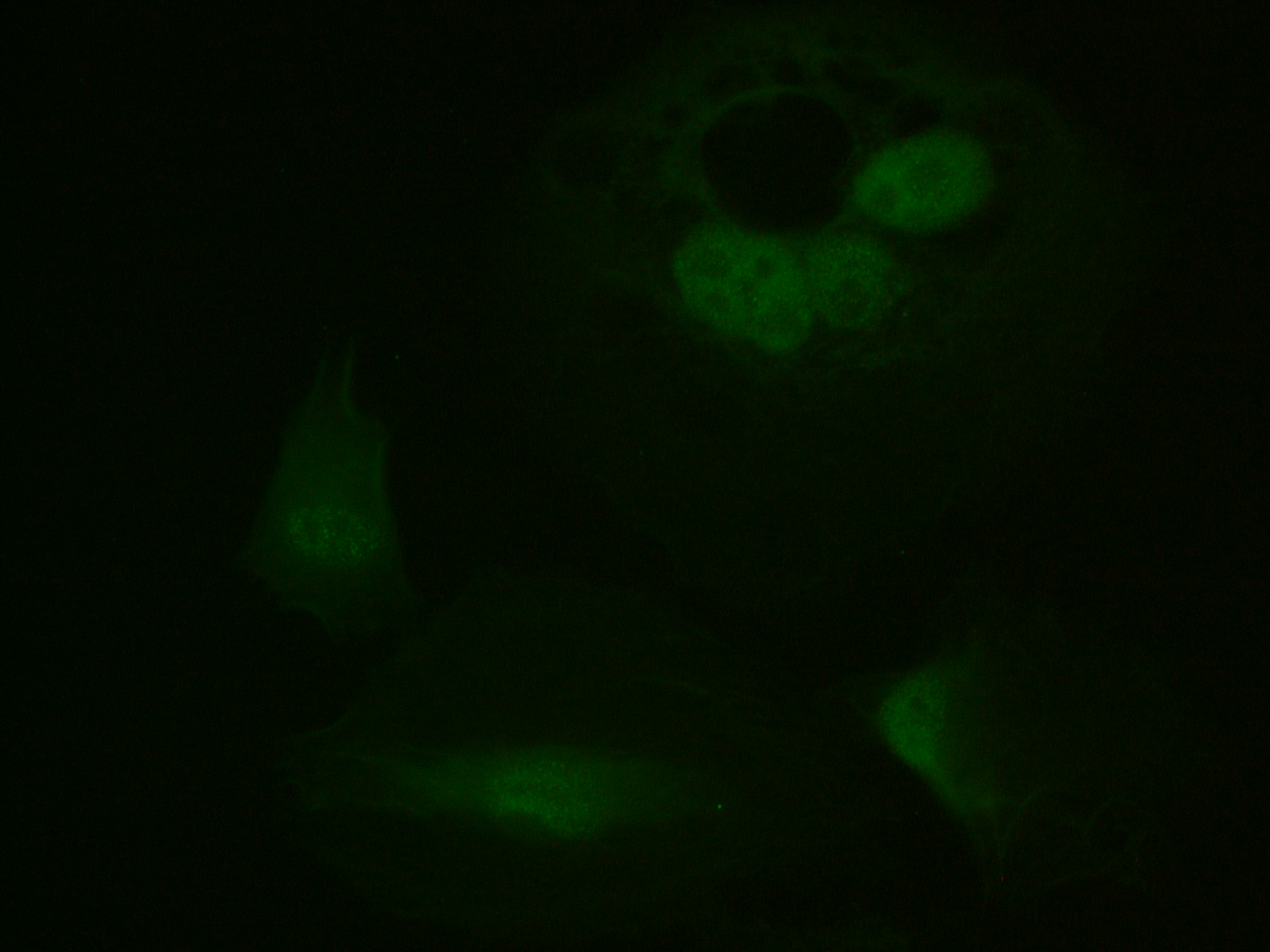

Supplement: S2 File — (ZIP) [file pone.0302213.s002.zip › FIGURA9/PROFHILO/els_40x.tif]

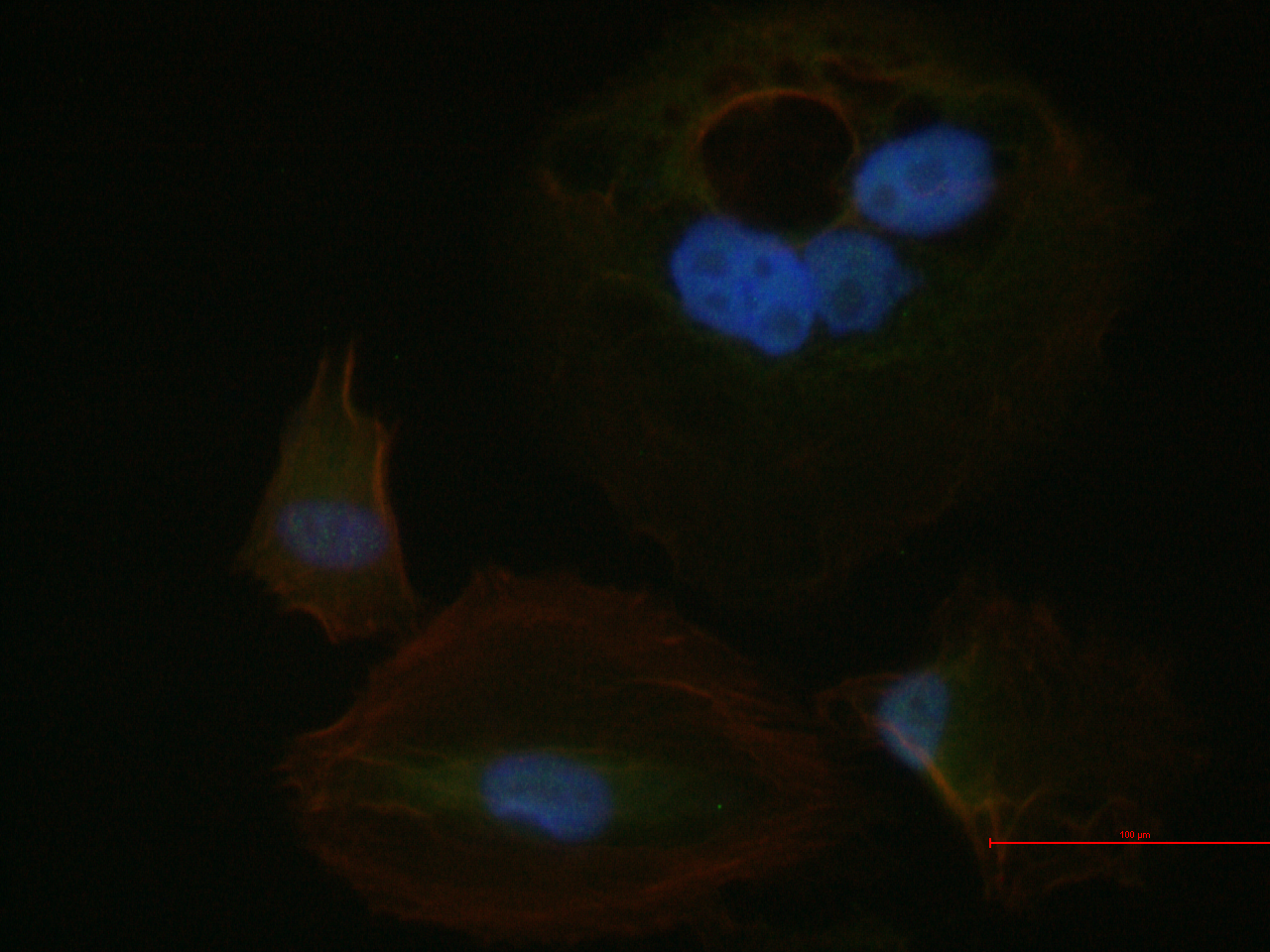

Supplement: S2 File — (ZIP) [file pone.0302213.s002.zip › FIGURA9/PROFHILO/Miscelato40x.tif]

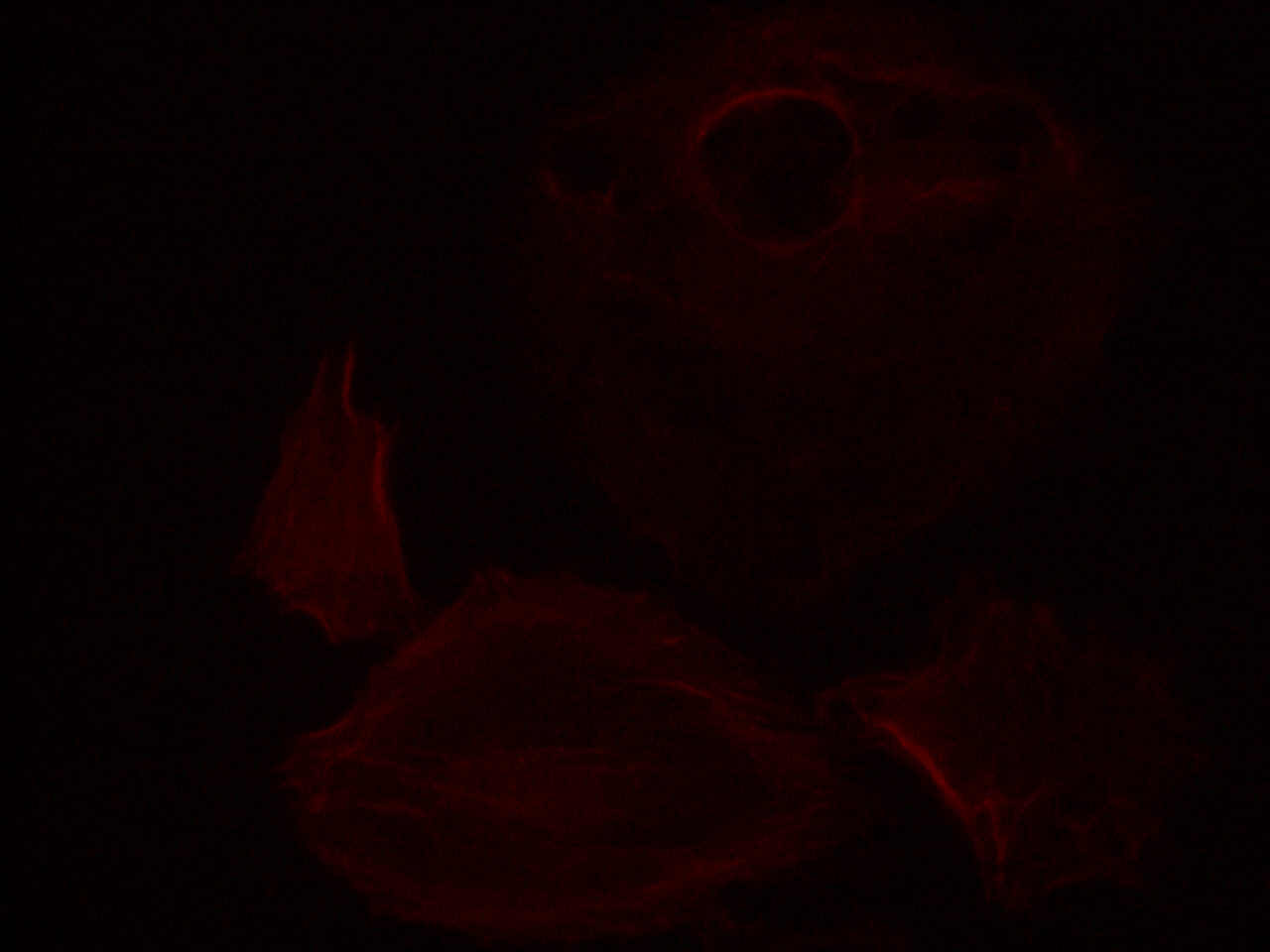

Supplement: S2 File — (ZIP) [file pone.0302213.s002.zip › FIGURA9/PROFHILO/tritc_40x.tif]
